# Optimized isolation of 7,7′-biphyscion starting from *Cortinarius rubrophyllus*, a chemically unexplored fungal species rich in photosensitizers

**DOI:** 10.1007/s43630-021-00159-y

**Published:** 2021-12-31

**Authors:** Fabian Hammerle, Lisa-Maria Steger, Xuequan Zhou, Sylvestre Bonnet, Lesley Huymann, Ursula Peintner, Bianka Siewert

**Affiliations:** 1 Pharmacology and Pharmacognosy, Center for Molecular Biosciences Innsbruck, University of Innsbruck, Innrain 80/82, 6020 Innsbruck, Austria; 2 Gorlaeus Laboratories, Leiden Institute of Chemistry, Leiden University, P.O Box 9502, 2300 RA Leiden, The Netherlands; 3 University of Innsbruck, Microbiology, Technikerstraße 25, 6020 Innsbruck, Austria

## Abstract

Mushrooms such as the dermocyboid *Cortinarius rubrophyllus* are characterized by strikingly colorful fruiting bodies. The molecular dyes responsible for such colors recently experienced a comeback as photoactive compounds with remarkable photophysical and photobiological properties. One of them—7,7′-biphyscion—is a dimeric anthraquinone that showed promising anticancer effects in the low nanomolar range under blue-light irradiation. Compared to acidic anthraquinones, 7,7′-biphyscion was more efficiently taken up by cells and induced apoptosis after photoactivation. However, seasonal collection of mushrooms producing this compound, low extraction yields, and tricky fungal identification hamper further developments to the clinics. To bypass these limitations, we demonstrate here an alternative approach utilizing a precursor of 7,7′-biphyscion, i.e., the pre-anthraquinone flavomannin-6,6′-dimethyl ether, which is abundant in many species of the subgenus *Dermocybe.* Controlled oxidation of the crude extract significantly increased the yield of 7,7′-biphyscion by 100%, which eased the isolation process. We also present the mycochemical and photobiological characterization of the yet chemically undescribed species, i.e. *C.* *rubrophyllus*. In total, eight pigments (**1**–**8**) were isolated, including two new glycosylated anthraquinones (**1** and **2**). Light-dependent generation of singlet oxygen was detected for the first time for emodin-1-*O*-β-d-glucopyranoside (**3**) [photophysical measurement: *Φ*_∆_ = 0.11 (CD_3_OD)]. Furthermore, emodin (**7**) was characterized as promising compound in the photocytotoxicity assay with EC_50_-values in the low micromolar range under irradiation against cells of the cancer cell lines AGS, A549, and T24.

**Graphical abstract:**

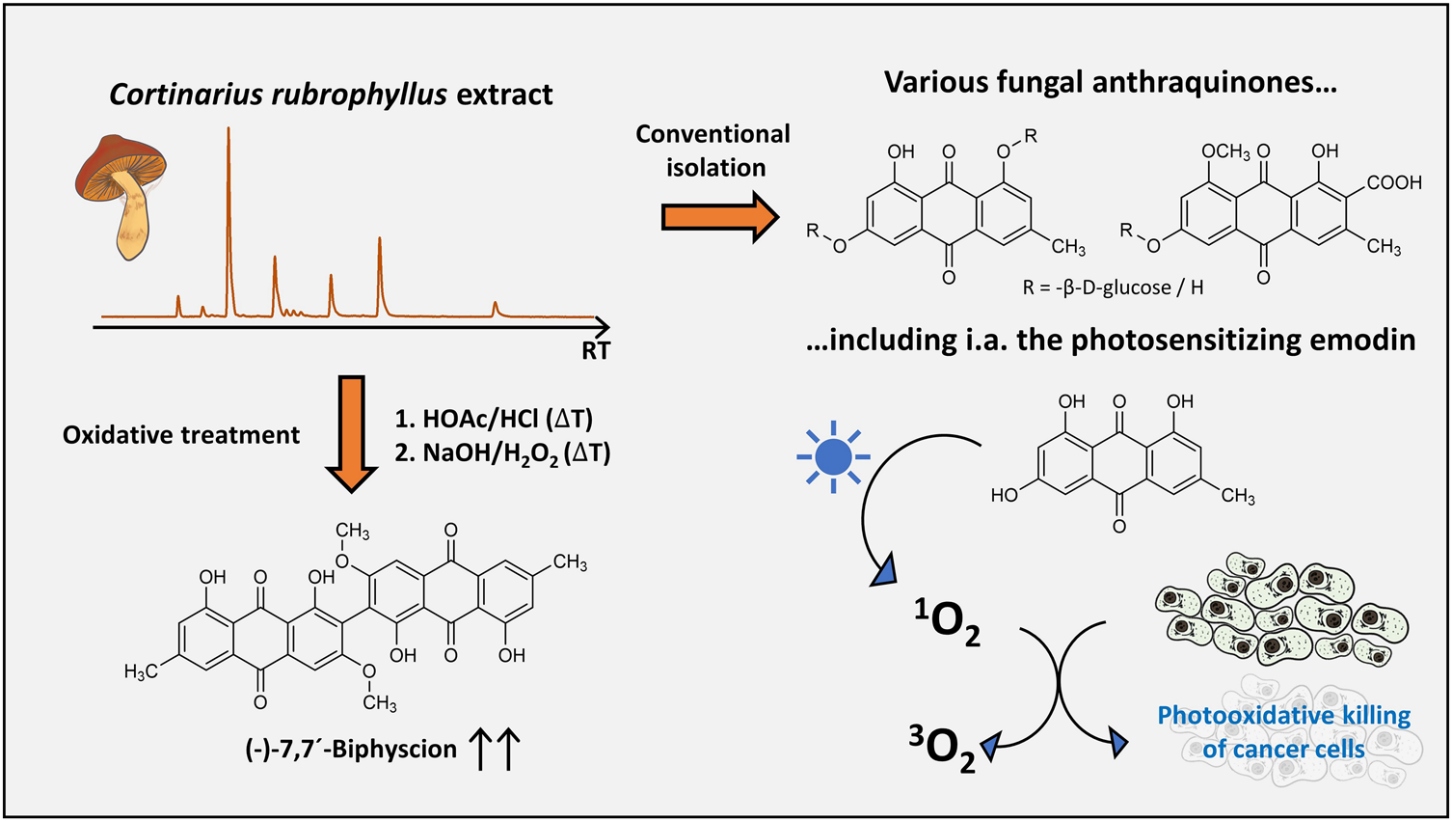

**Supplementary Information:**

The online version contains supplementary material available at 10.1007/s43630-021-00159-y.

## Introduction

Without ectomycorrhizal (ECM) fungi, such as the widespread and species-rich genus *Cortinarius* [1], terrestrial ecosystems as we know them would not be able to exist. ECM fungi, which form complex structures within the roots of their symbiotic partner plants (= Hartig net) as well as an external multi-layered hyphal structure (= mantle/sheath) [2], are the backbone of carbon and nutrient cycles in forests, as they are directly involved in the mobilization, absorption, and translocation of soil nutrients and water [3]. Some species, e.g., *Tuber* *borchii* and *Lactarius* *bicolor*, are even able to control root morphology via the formation of 2-(1*H*-indol-3-yl)-acetic acid and ethylene [4, 5]. However, there are species that have another interesting feature that is far more striking than networks hidden in the ground, as it is a real eye-catcher: brightly colored fruiting bodies.

In recent years, research revealed an unexpected effect associated with the bright colors of fungi: photoactivity [6]. The fruiting body extracts from *Cortinarius* mushrooms belonging to the classical subgenera *Dermocybe* and *Phlegmacium* showed promising blue and green light-induced cytotoxicity against various cancer cell lines in the low µg/mL-range [7]. The dimeric anthraquinone 7,7′-biphyscion, which was isolated from *Cortinarius uliginosus*, for example, exhibited an astounding anticancer activity after blue-light irradiation with an EC_50_-value as low as 64 nM against A549 lung cancer cells (*λ*_irr_ = 468 nm, H (radiant exposure) = 9.3 J cm^−2^) and was able to induce dose-dependent apoptosis [8]. Furthermore, the acetone extract of *Cortinarius xanthophyllus* was also able to completely inhibit the growth of the gram-positive bacterium *Staphylococcus aureus* (*c* = 7.5 µg/mL, *λ*_irr_ = 478 nm, *H* = 30 J cm^−2^) and of the yeast *Candida albicans* (*c* = 75 µg/mL, *λ*_irr_ = 478 nm, *H* = 30 J cm^−2^) in combination with visible light [9]. Due to this wide variety of promising bioactivities, it can be assumed that a huge photopharmacological potential lies dormant in ECM fungi waiting to be explored. Humans could also benefit from these compounds, just like the mushrooms and their symbiotic partners in the forest do.

Nevertheless, the ectomycorrhizal lifestyle is unfavorable for upscaling processes, as it denies a convenient biotechnical production and requires season-dependent field trips instead. Furthermore, the exact identification of these colorful fungi (i.e., dermocyboid Cortinarii) is intricate and demands expert knowledge. Thus, an alternative approach to conveniently obtain the promising photosensitizer 7,7′-biphyscion is needed.

Here, we present the optimized isolation of 7,7′-biphyscion starting from dermocyboid species containing the unstable biosynthetic precursor of 7,7′-biphyscion, i.e., flavomannin-6,6′-di-*O*-methyl ether (FDM). In detail, (i) the strategy was established utilizing a species common in Tyrol (Austria), namely *Cortinarius rubrophyllus*. As the pigment profile of this fungus had not yet been investigated in detail, the unexplored pigments were isolated and characterized. (ii) The strategy was validated with another dermocyboid species, i.e., the yellow webcap *C. holoxanthus.* (iii) The strategy’s robustness was tested with a pooled assembly of collected dermocyboid Cortinarii.

## Materials and methods

### Chemicals

All solvents for the extraction procedures and thin-layer-chromatographic analyses were of analytical grade and sourced from VWR International (Vienna, Austria). Acetone was distilled prior to its use. Solvents used for HPLC experiments were purchased from Merck (Merck KGaA, Darmstadt, Germany). TLC plates (ALUGRAM^®^ Xtra SIL G/UV _254_ 20 × 20 cm) were obtained from Macherey–Nagel (Macherey–Nagel GmbH & Co. KG, Düren, Germany). Ultrapure water was produced by a Sartorius arium^®^ 611 UV purification system (Sartorius AG, Göttingen, Germany). NMR solvents were purchased from Euriso-top SAS (Saint-Aubin Cedex, France). The reagents 9,10-dimethylanthracene (product number: D0252), acid red 52 (sulforhodamine B, product number: A0600), and emodin (product number: E0500) were sourced from TCI Deutschland GmbH (Eschborn, Germany). Acridine orange (product number: 0249.1) and ethidium bromide (product number: 7870.1) were from Carl Roth GmbH & Co. KG (Karlsruhe, Germany). Expendable materials (e.g., flasks, etc.), media, and supplements (i.e. fetal bovine serum, penicillin/streptomycin, trypsin, phosphate-buffered saline) used for cell culture maintenance and the (photo)cytotoxicity experiments were purchased from Thermo Fischer Scientific (Waltham, Massachusetts, USA).

### General instrumentation

The weighing instruments Sartorius Cubis®-series (Sartorius AG, Göttingen, Germany) and Mettler Toledo AB54 (Mettler-Toledo GmbH, Gießen, Germany) were used for weighing samples. The ultrasonic baths Sonorex RK 52 (BANDELIN electronic GmbH & Co. KG, Berlin, Germany) and Sonorex RK 106 were employed for extract preparation. A Heidolph Laborota 4000 efficient rotary evaporator (Heidolph Instruments GmbH & Co. KG, Schwabach, Germany) coupled to a vacuubrand PC 101 NT (VACUUBRAND GmbH & Co. KG, Wertheim, Germany) vacuum pump was used for evaporation of solvents. The power adaptor Agilent E3611A DC Power Supply (Agilent Technologies, Inc., Santa Clara, United States) in combination with a LED-panel (*λ* = 468 ± 27 nm (20.6 mW cm^−2^) (University Leiden, published in Hopkins et al. [10]) was utilized. Absorption measurements were carried out with the Shimadzu UV-1800 spectrophotometer (Shimadzu Europa GmbH, Duisburg, Germany) or with the plate reader Tecan Spark^®^ 10 M (Tecan Group Ltd., Männedorf, Switzerland). The pH-meter Mettler Toledo SevenMulti (Mettler-Toledo GmbH, Vienna, Austria) was utilized for the adjustment of pH-values. Mixing of samples was done with the vortex mixer Vortex-Genie 2 (Scientific Industries, Inc., Bohemia, New York). Pipettes, as well as tips, were either from Eppendorf AG (Hamburg, Germany) or from STARLAB International GmbH (Hamburg, Germany). Reagent reservoirs were obtained from Thermo Fischer Scientific (Waltham, Massachusetts, USA). ^1^H and ^13^C NMR spectra were acquired using two spectrometers from Bruker, an Avance II 600 spectrometer operating at 600 MHz (^1^H) and 151 MHz (^13^C) at 300 K (chemical shifts in ppm, coupling constants *J* in Hz) and an Avance III HD spectrometer operating at 400 MHz (^1^H) (Bruker Corporation, Billerica, USA). IR spectra were recorded on an ALPHA FT-IR apparatus (Bruker, Ettlingen, Germany) equipped with a Platinum ATR module. Other specific instruments are listed in the respective chapters.

### High-performance liquid chromatography (HPLC)

HPLC–MS experiments were performed on the modular system Agilent Technologies 1260 Infinity II with a quaternary pump, vial sampler, column thermostat, diode-array detector, and mass spectrometer (Agilent Technologies, Inc., Santa Clara, USA) or on the Agilent Technologies 1260 HPLC system coupled to an amaZon iontrap mass spectrometer (Bruker, Bremen, Germany). A Synergi MAX-RP 80 Å column (150 × 4.60 mm, 4 micron) from Phenomenex (Aschaffenburg, Germany) was used as a stationary phase. The mobile phase comprised water (A) and acetonitrile with 0.1% formic acid (B). Elution was performed in gradient mode (0 min: 10% B, 3 min: 50% B, 5 min: 90% B, 7 min: 99% B, 11 min: 99% B, 11.1 min: 10% B, followed by 4 min of re-equilibration with 10% B). The DAD was set to a detection wavelength of *λ* = 430 nm, and flow rate, sample volume, and column temperature were adjusted to *Q* = 1.0 mL/min, *V* = 10 µL, and *T* = 40 °C, respectively. HPLC–DAD analyses were carried out on a Shimadzu LC-20AD XR system (Shimadzu Europa GmbH, Duisburg, Germany) or on an Agilent Technologies 1100 HPLC system, both equipped with a DAD detector, autosampler, and column thermostat. The chromatographic conditions were as described before. Extract components were identified using authentic reference samples [8] by evaluating compound-specific properties, such as retention time and mass (SI: Chapter 2.1.1).

### Fungal biomaterial and extract preparation

The fruiting bodies of *Cortinarius rubrophyllus* (Moënne-Locc.) Liimat., Niskanen, Ammirati & Dima were collected at different sites near Innsbruck (SI: Chapter 1, Table S1) in Tyrol, Austria, from August to September 2019. *Cortinarius holoxanthus* (M. M. Moser & I. Gruber) Nezdojm. was collected in September 2020 in Lans, Tyrol, Austria (GPS: 47° 14′18.4″ N/11° 25′54.5″ E) (SI: Chapter 1, Table S1). Biomaterial from other dermocyboid Cortinarii used for the pooled approach was collected in Tyrol, Austria, from August 2019 to September 2020 (SI: Chapter 1, Table S1). After collection, the material of the pooling experiment was immediately frozen. Taxonomic classification was performed by Lesley Huymann and Ursula Peintner using standard macroscopic and microscopic techniques as well as rDNA internal transcribed spacer (ITS) sequence analysis. An authenticated voucher specimen of all investigated Cortinarii is deposited in the mycological collection of the Tiroler Landesmuseen (IBF, official herbarium code). The material was shade-dried at room temperature after collection and subsequently stored in the dark until further analysis.

**Fig. 1:**
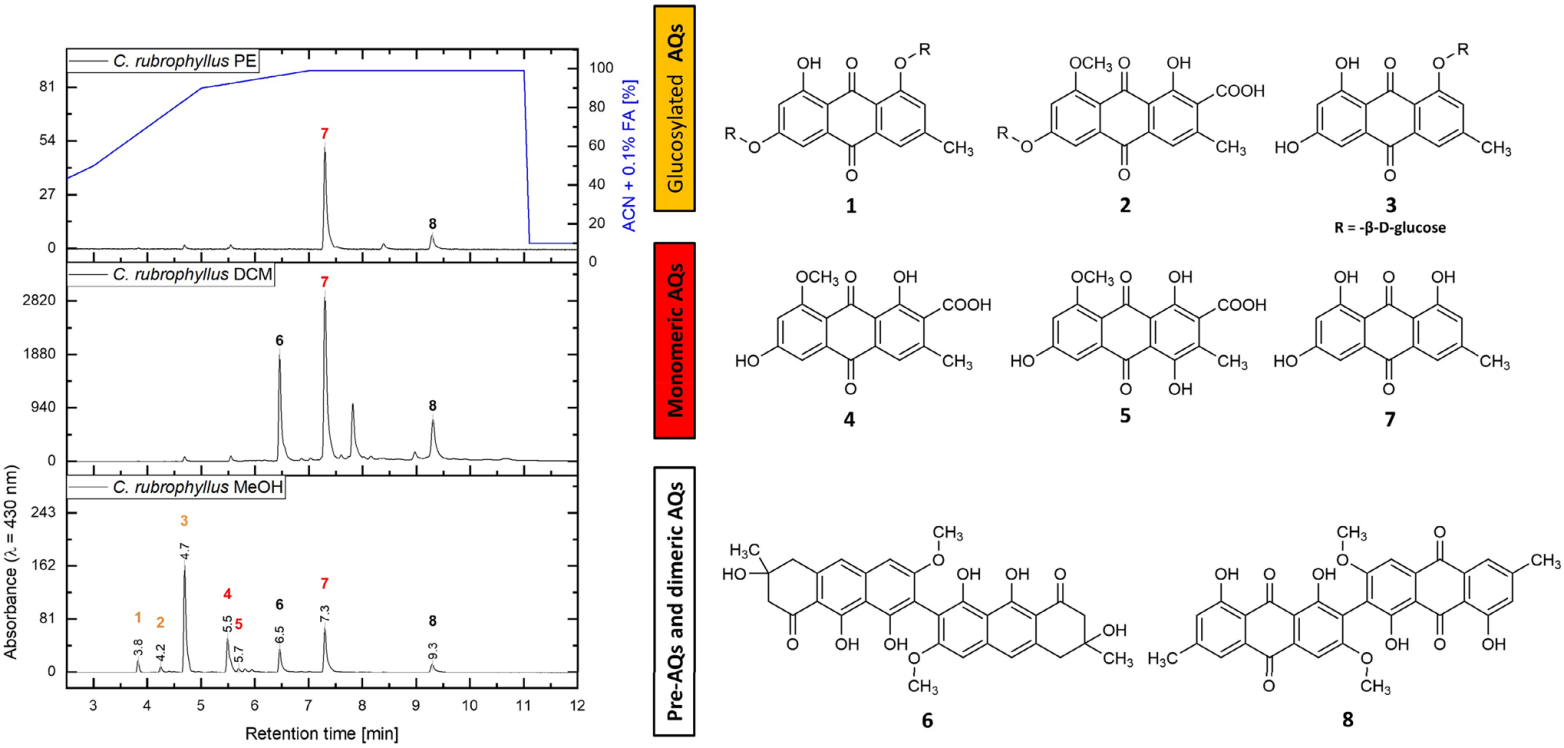
The chemical structures of the isolated compounds (**1–8**) are displayed on the right side (*R* = -β-d-glucose). Left, the chromatograms of the three sequential C. rubrophyllus extracts (recorded at *λ* = 430 nm) are depicted. The HPLC–DAD-MS analysis was conducted in gradient mode (A: H_2_O, B: ACN + 0.1% FA, blue line) with a Phenomenex Synergi MAX-RP 80 Å column (150 × 4.60 mm, 4 micron) column as stationary phase. Peaks corresponding to isolated and identified compounds were numbered and labelled (orange: glucosylated AQs, red: monomeric AQs, black: pre-AQs and dimeric AQs) with their respective retention times

Dried fruiting bodies (*m* = 42.0 g) of *C.* *rubrophyllus* were finely ground (mesh size 0.5 mm) and extracted successively with petroleum ether (*V* = 500 mL, *n* = 5), dichloromethane (*V* = 500 mL, *n* = 5), and methanol (*V* = 500 mL, *n* = 5) using an ultrasonic bath (10 min per extraction step) followed by filtration. Respective filtrates were combined, solvents were removed by vacuum rotary evaporation at 40 °C, and the combined extracts were kept in a desiccator. The extracts yielded *η* = 500.0 mg (1.19% w/w) of petroleum ether extract, *η* = 724.2 mg (1.72% w/w) of dichloromethane extract, and *η* = 9082.8 mg (21.63% w/w) of methanol extract (SI: Table S2 and Fig. 1). The methanolic extract was further separated via liquid–liquid fractionation. An aliquot (*m* = 2012.7 mg) was dissolved in water (*V* = 200 mL) and transferred to a separating funnel. The solution was partitioned with diethyl ether (*V* = 200 mL, *n* = 5) and ethyl acetate (*V* = 200 mL, *n* = 4). The fractions were evaporated to dryness under reduced pressure at 40 °C and stored in a desiccator. The yields of the liquid–liquid extraction were as follows: diethyl ether *η* = 749.9 mg (L1, 37.3% w/w), ethyl acetate *η* = 231.3 mg (L2, 8.5% w/w), and water *η* = 1031.5 mg (L3, 51.2% w/w).

### Isolation of secondary metabolites

The isolation of compounds **1–8** (Fig. 1, Table S3) from *C.* *rubrophyllus* is thoroughly discussed in the supplementary material (SI: Chapter 2.1.2). Briefly, compounds **1** (*η* = 2.2 mg, 0.11% w/w_MeOH_) and **2** (*η* = 1.5 mg, 0.07% w/w_MeOH_) were isolated from the water fraction (L3), which resulted from the liquid–liquid fractionation of the methanolic extract, utilizing size exclusion chromatography (Sephadex LH-20 column chromatography) and preparative thin-layer chromatography (HPLC analysis: see SI, Figure S1). Compound **3** (*η* = 12.0 mg, 0.59% w/w_MeOH_) as well as a mixture of compounds **4** and **5** (*η* = 4.4 mg, 0.22% w/w_MeOH_) were isolated from the ethyl acetate fraction (L2) employing acetylated polyamide column chromatography (SI: Figure S2 and Table S4). Compounds **6** (*η* = 7.4 mg, 0.37% w/w_MeOH_) and **7** (*η* = 2.6 mg, 0.13% w/w_MeOH_) were isolated from the dichloromethane extract by employing acetylated polyamide and Sephadex LH-20 column chromatography. Compound **8** (*η* = 2.4 mg, 0.12% w/w_MeOH_) was obtained from the dichloromethane extract via a newly developed method (see 2.9).

### GC–MS analysis of the *C. rubrophyllus* methanol extract

Structure elucidation of **1**, **2**, and **3** pointed towards β-glucose-substituted AQs. To determine the absolute configuration of the glucose residues, a GC–MS analysis of the hydrolyzed and subsequently derivatized extract was carried out. An aliquot of the crude methanolic extract of *C.* *rubrophyllus* (*m* = 10.1 mg) was dissolved in aqueous trifluoroacetic acid (TFA, *c* = 3 mol/L, *V* = 1 mL) and heated at 90 °C for 60 min. After cooling and adding 2 mL of water, the reaction mixture was extracted with ethyl acetate (*V* = 1 mL, *n* = 3). The aqueous phase was dried under an air stream at room temperature and kept in a desiccator. D-Glucose (*m* = 1 mg, *n* = 5.5 µmol), l-glucose (*m* = 1 mg, *n* = 5.5 µmol), and the dried hydrolysate were derivatized with l-cysteine methyl ester hydrochloride [1.5 mg (8.7 µmol) in 200 µL pyridine, *T* = 60 °C, *t* = 60 min], subsequently silylated with *N,O*-bis(trimethylsilyl)trifluoroacetamide and chlorotrimethylsilane (BSTFA:TMCS = 99:1, 200 µL, *T* = 60 °C, *t* = 60 min), and analyzed by GC–MS. GC–MS analysis was carried out on an Agilent 5975C Series GC/MSD System equipped with an Agilent 7693 autosampler and a Triple Axis-Detector (MS). An Agilent 19091S-433: 1813.75629 HP-5MS 5% Phenyl Methyl Silox (325 °C: 30 m × 250 µm × 0.25 µm) was used as a stationary phase and helium was chosen as carrier gas. The oven temperature was initially set to *T* = 120 °C. With a heating rate of 4 °C/min, the oven reached *T* = 270 °C. After two minutes, the oven was heated to 320 °C with 20 °C/min and kept constant for *t* = 5 min. The total run time, injection volume, split ratio, and flow rate were set to *t* = 52 min, *V* = 1 µL, 50:1, and *Q* = 0.75 mL/min, respectively.

### Photophysical characterization

The quantum yield of singlet oxygen generation was measured with a custom-built setup described elsewhere [11]. Optical fibers with a diameter of 600 µm from Avantes (Avantes BV, Apeldoorn, The Netherlands) were used to connect all optical parts. Samples were dissolved in deuterated methanol (*V* = 500 µL), transferred into a semi-micro cuvette from fireflysci (FireflySci, Inc., Staten Island/NY, USA) with *l* = 4 mm and *l* = 10 mm path lengths, and placed in a CUV-UV/VIS-TC temperature-controlled cuvette holder (Avantes). Samples were allowed to equilibrate at *T* = 20 °C. Emission spectroscopy was performed with a fiber-coupled laser (*λ* = 450 nm, LRD-0450, Laserglow), which was set to *P* = 50 mW or 15 mW at the cuvette (4 mm beam diameter, 0.4 W cm^−2^) at a 90° angle with respect to the spectrometer. The excitation power was measured using a S310C thermal sensor connected to a PM100USB power meter (Thorlabs Inc., Newton, USA). The emission spectra were recorded using a spectrometer for NIR emission, i.e., from *λ* = 300 to 1000 nm for the luminescence (Avantes 2048L StarLine spectrometer) and from *λ* = 1000 to 1700 nm for the phosphorescence of singlet oxygen (^1^Δ_g_) around *λ* = 1275 nm (Avantes NIR256-1.7TEC spectrometer, detector set to − 11 °C). The infrared emission spectrum was acquired within 9 s. UV–Vis absorption spectra before and after emission spectroscopy were measured using an Avalight-DHc halogen-deuterium lamp (Avantes) as light source (turned off during emission spectroscopy) and with an Avantes 2048L StarLine spectrometer as a detector, both connected to the cuvette holder at a 180° angle. Avasoft 8.5 software was used to record all spectra, and further processing was done with Microsoft Excel 2010 and Origin Pro 9.1 software. The quantum yield of singlet oxygen production was calculated using the relative method with [Ru(bpy)_3_]Cl_2_ (*Φ*_Δ_ = 0.73, *Φ*_P_ = 0.015 in CD_3_OD) [11–13] as standard according to following equation: *Φ*_sam_ = *Φ*_std_ × (*A*_std_450)/(*A*_sam_450) × *E*_sam_/*E*_std_. *Φ* represents the quantum yield, A450 is the absorbance at *λ* = 450 nm [always kept between 0.100 and 0.093 for (l = 4 mm path length)], *E* is the integrated emission peak of singlet oxygen at *λ* = 1270 nm, and sam and std denote the sample and standard, respectively.

### Cell culture maintenance and (photo)cytotoxicity assay

Cells of the adherent cancer cell lines A549 (non-small cell lung cancer, ATCC, Sigma-Aldrich), AGS (stomach cancer, CLS, Eppelheim), and T24 (urinary bladder carcinoma, CLS, Eppelheim) were cultivated in 75 cm^2^ Nunc EasYFlasks (product number: 51985042, 75 cm^2^) with Gibco™ MEM™-medium (product number: 42360081) supplemented with fetal calf serum (FCS, 10% v/v) and penicillin/streptomycin (P/S, 1% v/v). Cells were trypsinized every other day (confluency $\sim$ 80%) and used for 8–12 weeks. Freezing and de-freezing of cell cultures were done according to standard procedures. The (photo)cytotoxicity assay was performed as published elsewhere [10].

Briefly, cells (AGS: 2500 cells/well, T24 and A549: 2000 cells/well) were seeded in 96-well plates in Gibco™ Opti-MEM™ (OMEM, product number: 11058021) containing FCS (2.4% v/v) and P/S (1% v/v) at 37 °C in 5% CO_2_ atmosphere. Compounds **3**, **6**, and **7** were dissolved in DMSO (c_stock solution_ = 5 mM) and further diluted with OMEM. Treatment was conducted 24 h after the seeding step with six different working solutions per compound. The final concentrations tested were 25, 12.5, 5, 2.5, 1.25, and 0.25 µmol/L (max. concentration of DMSO = 0.5%). After an incubation period of 24 h, the medium was aspirated and replaced by fresh OMEM (+ 2.4% v/v FCS, + 1% P/S). Subsequently, the plates were irradiated for 7.5 min with blue light (*λ* = 468 nm ± 27 nm, *H* = 9.3 J cm^−2^). After the irradiation step, the plates were kept at 37 °C in a 5% CO_2_ atmosphere for another 48 h (total experiment time = 96 h). Then, the cells were fixed by careful addition of cold trichloroacetic acid (10% w/v in water, *V* = 100 µL/well) and stored in a refrigerator at *T* = 8 °C for at least 24 h. The fixed cell-monolayers were washed with slow running deionized tap-water and stained with sulforhodamine B (SRB) (acid red 52, 0.4% w/v SRB in 1% v/v acetic acid, *V* = 100 µL/well) for 30 min. Thereafter, the plates were washed again (*n* = 5, 1% v/v acetic acid) and dried at room temperature. Then, the dried dye was dissolved in tris(hydroxymethyl)aminomethane-solution (TRIS, 10 mM in water, *V* = 100 µL/well) and incubated for at least 20 min. Absorbance was measured at *λ* = 540 nm with a plate reader. EC_50_ values, including their confidence intervals (95%) were calculated with GraphPad Prism 5 employing the relative Hill-Slope equation with variable slope. As negative control served the illuminated, non-treated cells as well as the non-illuminated, non-treated cells. As positive control served berberine (see Table S6). The photoindex (P.I.), which expresses the ratio of cells killed in the absence of light to cells killed after irradiation, was calculated as EC_50|dark_ divided by EC_50|irradiated_.

#### Acridine orange/ethidium bromide (AO/EB)-assay

An AO/EB-assay was conducted as published elsewhere [14] to preliminarily investigate the mechanism of cell death induced by blue light-activation of emodin (**7**). T24 cancer cells were seeded in 6-well plates (100,000 cells/well) in OMEM containing FCS (2.4% v/v) and P/S (1% v/v) at 37 °C in 5% CO_2_ atmosphere. 24 h after seeding, the cells were treated with **7** (final concentration in the well: 5 µM) and incubated for another 24 h. Then, the treatment solution was aspirated, replaced with fresh supplemented OMEM, and the cells were irradiated with blue light (*λ* = 468 nm ± 27 nm, H = 9.3 J cm^−2^). After 24 additional hours of incubation, the supernatant of a single well was transferred to a 15 mL falcon tube, centrifuged, and the solution decanted. The cell pellet was suspended in the remaining drops of the medium in the falcon tube. The AO/EB-dye mix was prepared from the solutions of acridine orange (*c* = 100 µg/mL) and ethidium bromide (*c* = 100 µg/mL) in phosphate-buffered saline by mixing equal volumes of both solutions. The dye mix (V = 1 µL) was added to the cell suspension (*V* = 25 µL), gently mixed, and a part of the generated suspension (*V* = 10 µL) was placed onto a clean microscope slide. Analysis of membrane integrity was carried out by fluorescence microscopy, using blue light for excitation.

### In situ generation and isolation of 7,7′-biphyscion (8)

An aliquot of the dichloromethane extract (*m* = 50.3 mg) was dissolved in a mixture of glacial acetic acid (*V* = 5 mL) and concentrated hydrochloric acid (*V* = 0.2 mL) and refluxed (*T*_water bath_ = 100 °C) for 1 h. Subsequently, the solution was extracted with diethyl ether (*V* = 20 mL, n = 2) and dichloromethane (*V* = 15 mL, *n* = 2). Both organic phases were combined, the solvents were removed by vacuum rotary evaporation at 40 °C, and the resulting extract was kept in a desiccator (B1, *η* = 47.1 mg, 93.6% w/w). B1 (*m* = 46.1 mg) was dissolved in a mixture of sodium hydroxide solution (c = 1 mol/L, *V* = 15 mL) and 30% hydrogen peroxide solution (*V* = 0.3 mL) and heated to 60 °C for 3 h. Monitoring the presence of compound **8** (*R*_f_ = 0.75) was conducted by TLC (SiO_2_, toluene/methanol/ethyl acetate/formic acid = 94:2.5:2.5:1). Then, the mixture was acidified with acetic acid, diluted with water (*V* $\sim$ 50 mL), and extracted with diethyl ether (*V* = 100 mL, *n* = 2). The diethyl ether fraction was dried via vacuum rotary evaporation at 40 °C and kept in a desiccator (B1.1, *η* = 54.9 mg).

The fraction B1.1 (*m* = 54.9 mg) was subjected to dry column vacuum chromatography ($\varnothing$ = 5.5 cm, *l* = 3.5 cm). Isocratic elution was performed under reduced pressure with silica gel 60 (0.040–0.063 mm) purchased from Merck as a stationary phase. First, petroleum ether was used as a mobile phase for defatting purposes, followed by the solvent mixture toluene/methanol/ethyl acetate/formic acid = 94:2.5:2.5:1. When the yellow-stained sample band had passed approx. 80% of the separation distance, 50 fractions of 10 ml each (B1.1.1–B1.1.50) were collected. After analyzing the fractions via TLC (SiO_2_, toluene/methanol/ethyl acetate = formic acid = 94:2.5:2.5:1), fraction B1.1.1 to B1.1.14 were combined and the solvents were removed by vacuum rotary evaporation at 40 °C (B2, *η* = 8.8 mg). Fraction B2 (*m* = 8.8 mg) was suspended in petroleum ether (*V* = 2 mL) and filtrated through cotton wool. The filtrate cake was dissolved in dichloromethane and the solvent was removed under an air stream at room temperature to yield 2.4 mg (4.8% w/w) of compound **8**.

#### Isolation of compound 8 from *Cortinarius holoxanthus*

An aliquot of the *C.* *holoxanthus* dichloromethane extract (*m* = 8.4 mg) was treated as described in SI chapter 4.1.4. The first step involving acetic acid and hydrochloric acid yielded fraction C1 (*η* = 7.7 mg, 91.7% w/w). A part of C1 (*m* = 7.5 mg) was used up in the second step (NaOH / H_2_O_2_), resulting in 7.2 mg (85.7% w/w) of fraction C1.1.

4.0 mg of the fraction C1.1 were submitted to preparative TLC (pre-coated TLC sheets, 10 × 20 cm, silica gel 60 F254 0.20 mm layer) with toluene/ethyl acetate/formic acid/acetic acid (60:30:5:5) as mobile phase. The aliquot was dissolved in 1 mL of a mixture of chloroform, acetone, and methanol (1:1:1) and loaded onto four TLC plates. Every plate was developed once, keeping the separation distance between 8 and 9 cm. The area corresponding to compound **8** was removed (*R*_f_ = 0.9), extracted with chloroform via ultrasonication, and the extract was filtrated through cotton wool. Thereby, 0.6 mg (7.1% w/w) of **8** were obtained.

#### Preparation of a DCM extract from pooled *Cortinarius* biomaterial and in situ generation of compound 8

Fruiting bodies belonging to different dermocyboid *Cortinarius* species (i.e. *C.* *bataillei* (*m* = 655.2 mg), *C.* *pinicola* (*m* = 417.0 mg), *C.* *rubrophyllus* (565.9 mg), *C.* *sanguineus* (*m* = 325.6 mg), and *C.* *semisanguineus* (*m* = 449.4 mg)) were freeze-dried, finely ground with mortar and pestle, and successively extracted with petroleum ether (*V* = 150 ml, n = 2) and dichloromethane (*V* = 200 ml, *n* = 4). The extracts were filtered and dried via vacuum rotary evaporation at 40 °C. The yields were as follows: PE—*η* = 34.1 mg (1.4% w/w) and DCM—*η* = 55.7 mg (2.3% w/w). An aliquot of the DCM extract (*m* = 13.9 mg) was treated as described in chapter 2.9. The first step yielded fraction P1 (*η* = 13.7 mg, 98.6% w/w). 13.4 mg of P1 were used for the second step, resulting in 8.7 mg (62.6% w/w) of fraction P1.1. Aliquots of both fractions were dissolved in DMSO (*c* = 2 mg/mL) and subjected to HPLC–DAD analysis (parameters: Chapter 2.2).

## Results and discussion

This study aimed at establishing a convenient strategy for the isolation of 7,7′-biphyscion (**8**), i.e., the photoactive dimeric anthraquinone (AQ) of *C. uliginosus*. This mushroom species, however, is not ubiquitously distributed in Europe. Thus, an alternative approach starting with widespread species was envisioned. In Tyrol, one of such common species was *C. rubrophyllus*. As this species was not mycochemically investigated, the respective isolation, identification, and characterization processes were performed in the first step. After that, the in situ oxidation of flavomannin-6,6′-dimethyl ether (**6**) was established. Finally, another species (*C. holoxanthus*) and even a mix out of several dermocyboid Cortinarii were utilized to confirm the widespread applicability of in situ generation of 7,7′-biphyscion (**8**).

### Isolation and identification of isolated secondary metabolites

Dried and ground fruiting bodies of *C.* *rubrophyllus* were extracted sequentially with solvents of different polarities (i.e., first petroleum ether, then dichloromethane, and finally methanol) (Fig. 1). Subsequent purification steps of the extracts led to the isolation of two new glycosylated anthraquinones (AQs, see Fig. 1), emodin-1,6-di-*O*-β-D-glucopyranoside (**1**) and dermolutein-6-*O*-β-d-glucopyranoside (**2**), along the known compounds emodin-1-*O*-β-d-glucopyranoside (**3**), dermolutein (**4**), dermorubin (**5**), flavomannin-6,6′-dimethyl ether (**6**), emodin (**7**), and 7,7′-biphyscion (**8**).

**Fig. 2:**
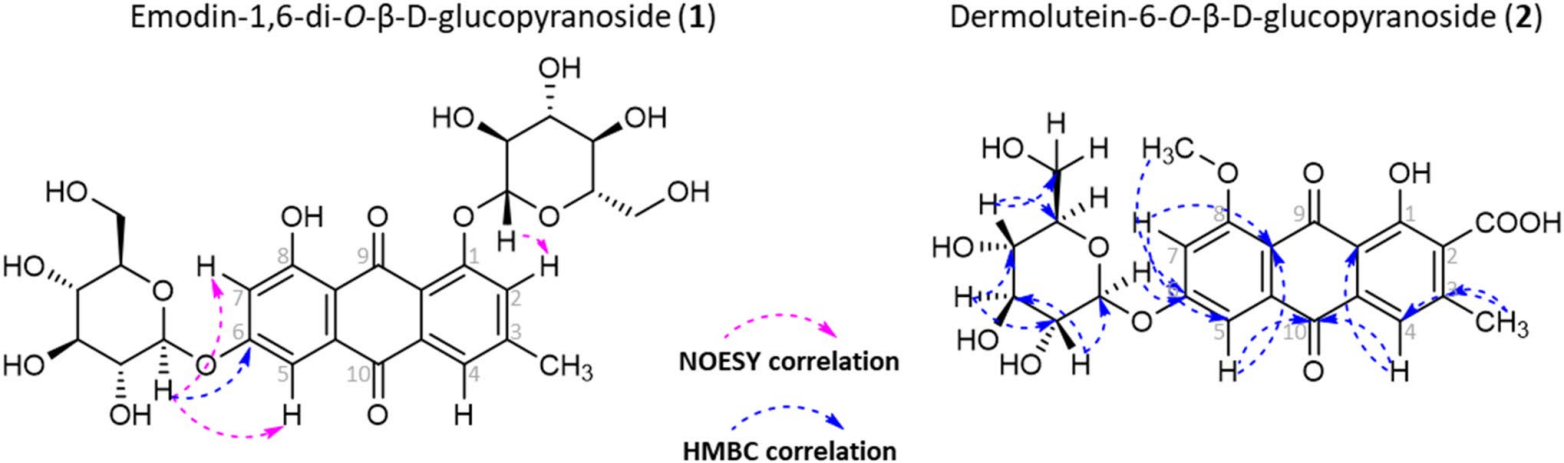
NOESY and HMBC key correlations observed for compounds **1** and **2**

Structure elucidation of **1** and **2** was achieved via 1D- and 2D-NMR experiments as well as HPLC–DAD-MS analysis. ^13^C chemical shift values were based on the HSQC and HMBC experiments, thus not all carbon atoms were assigned. The NMR data in tabular form and figures representing the 2D experiments are provided in the SI (Chapter 2.1.6). Compound **1** was isolated as a yellow solid. The ESI–MS spectrum (negative ionization mode) exhibited molecular ions at m/z 629.0, 656.0, 593.0 ([M-H]^−^), and 431.0 ([M-glucose-H]^−^). Since the ion at m/z 431.0, corresponding to compound **3**, was observed with high intensity and most likely resulted from losing a glucose unit from the ion at m/z 593.0, the molecular formula C_27_H_30_O_15_ was proposed for compound **1**. The UV/Vis spectrum showed maxima typical for AQs at *λ*_max_ = 221, 264, and 414 nm. The ^1^H NMR spectrum (SI: Table S5) exhibited four signals in the aromatic region, where the signals at *δ* = 6.84 and 6.62 ppm were doublets with coupling constants of *J* = 2.4 Hz. Thus, these *meta*-coupled protons were assigned to the aromatic carbons at positions 5 (*δ* = 107.7 ppm) and 7 (*δ* = 110.0 ppm). The signals at *δ* = 7.19 and 7.13 ppm were broad singlets and corresponded to the protons in positions 2 (*δ* = 124.2 ppm) and 4 (*δ* = 123.2 ppm). At *δ* = 2.31 ppm, a singlet was observed corresponding to three protons and thus was assigned to a methyl group. Based on the comparison of the UV/Vis spectra, MS spectra, and the ^1^H NMR signals of compounds **1** and **3**, an emodin moiety was proposed as the core structure of compound **1**. In addition, signals from two anomeric protons at *δ* = 5.14 and 4.99 ppm with coupling constants of *J* = 7.6 and 6.8 Hz, respectively, were present. Furthermore, a complex group of signals at *δ* = 4.08–3.62 ppm was observed, equalling fourteen protons, and thus indicating two *O*-β-glucose substituents. HMBC and NOESY experiments were used to confirm the position of the glucose units at the AQ moiety. In the HMBC experiment, a correlation peak was observed for the anomeric proton at *δ* = 5.14 ppm with the carbon signal at *δ* = 162.1 ppm. The latter was identified as C-6 via NOESY and HSQC correlation of the anomeric proton with the signals at δ = 6.84 and 6.62 ppm (Fig. 2, left). Although the second anomeric proton at *δ* = 4.99 ppm did not show any HMBC correlations with aromatic carbons, its spatial correlation (NOESY) with the signal at *δ* = 7.19 helped to assign the glucose to position 1.

Compound **2** was isolated as an orange solid. The ESI–MS spectrum (negative ionization mode) showed molecular ions at m/z 489.0 ([M-H]^−^) and m/z 327.0. Since the theoretical subtraction of one glucose from this ion gives an ion at m/z 327.0 ([M-glucose-H]^−^), a molecular formula of C_23_H_22_O_12_ corresponding to a glucose-substituted dermolutein (**4**) was proposed. Even though Keller suggested the occurrence of this compound in various *Dermocybe* species in 1982 [15], scientific evidence was lacking. The UV/Vis spectrum of compound **2** (SI: Figure S6) exhibited a pattern with maxima at *λ*_max_ = 226, 271, and 422 nm, similar to that observed for compound **4** (SI: Figure S2). The ^1^H NMR spectrum showed three signals in the aromatic region. The signals at *δ* = 7.38 and 7.06 ppm were doublets with coupling constants of *J* = 2.4 Hz and thus belonged to *meta*-coupled protons (C-5 (*δ* = 107.4 ppm) and C-7 (*δ* = 106.5 ppm)). The singlet at *δ* = 7.58 ppm corresponded to the aromatic proton at position 4 (C-4, *δ* = 121.6 ppm). Two singlets were observed at *δ* = 4.01 and 2.50 ppm, characteristic of a methoxy- (C_ar_-8) and a methyl-group (C_ar_-3), respectively. In addition, a doublet corresponding to an anomeric proton was seen at *δ* = 5.34 ppm with a coupling constant of *J* = 7.7 Hz, indicating substitution with a β-glucose. The HMBC correlations of the singlet at *δ* = 4.01 ppm (OC*H*_3_-8) and the doublet at *δ* = 5.34 ppm (C*H*-1′) with the aromatic carbon at *δ* = 162.5 ppm (C-6) helped to assign the position of the glucose unit (Fig. 2). The complete assignment of the ^1^H and ^13^C NMR spectroscopic signals of the sugar carbons was established on the basis of one-bond and long-range experiments (HSQC, HMBC, and COSY) and is given in the SI (Table S5).

Emodin-1-*O*-β-d-glucopyranoside (**3**) was identified via comparing its physical and spectroscopic data with those reported in the literature [16, 17]. Compounds **1–3** contained all a sugar component. Analysis of coupling constants and chemical shift values of the corresponding sugar protons obtained from the ^1^H NMR and COSY experiments of **1–3** indicated the presence of *O*-β-glucose in all cases. To elucidate the absolute configuration of the glucose moieties, GC–MS analysis was conducted. First, the hydrolysate of the crude methanol extract was analyzed, showing that glucose was detectable as the major sugar unit. To prove its absolute configuration, an analysis of the respective thiazolidine derivative in comparison with the reference compounds D- and L-glucose was performed. Thus, compounds **1–3** were identified as emodin-1,6-di-*O*-β-D-glucopyranoside (**1**), dermolutein-6-*O*-β-D-glucopyranoside (**2**), and emodin-1-*O*-β-D-glucopyranoside (**3**) (SI: Figure S22).

Identification of compounds **4** and **5**, which were obtained as a mixture, was achieved with the help of previously isolated reference compounds [8] (SI: Figure S2). The structures of compounds **6**–**8** were confirmed via comparison with literature data as well as in-house data (**6**: [18], **7**: [19], **8**: [8, 20]).

Phylogenetic studies have shown that *C.* *rubrophyllus* belongs to the *Cortinarius* section *Dermocybe* and is closely related to *C.* *semisanguineus* and *C.* *malicorius* [21]. According to Keller's pigment classification [15], the previously uninvestigated *C. rubrophyllus* can be described as a malicoria-pigmentation-type, characterized by the presence of pre-AQs [e.g., flavomannin-6,6′-dimethyl ether (**6**)], AQ carboxylic acids [e.g., dermolutein (**4**) and dermorubin (**5**)], as well as the monomeric AQ emodin (**7**) and its glucoside (**3**). Looking at the results of our secondary metabolite investigation and thus from a chemotaxonomic point of view, the phylogenetic classification of *C.* *rubrophyllus* can be confirmed, as its pigment profile matches the profiles of both *C.* *semisanguineus* [22–24] and *C. malicorius* [15].

### Photophysical investigation

A preliminary mycochemical and photophyscial investigation of the methanol extract of *C.* *rubrophyllus* showed that the apolar diethyl ether and ethyl acetate fractions were far more photoactive than the hydrophilic ones (i.e., methanol and water) [25]. To identify the photophysical principle behind, the more apolar compounds (i.e., **3**, **6**, and **7**) were submitted to phosphorescence measurement of ^1^O_2_ at 1270 nm after activation with a 450 nm laser. The results are presented in Table 1.

**Table 1:** Singlet oxygen quantum yields of compounds **3–8** in CD_3_OD. Data from our previous study are provided for comparative reasons [8]

	*λ*_abs _^[a]^ [nm] (log ε)	*Φ*_Δ_^[b,c]^
Emodin-1-O-β-d-glucopyranoside (**3**)	428 (3.80)	11%
Dermolutein (**4**)*	427 (3.89), 440 (3.85)	3%
Dermorubin (**5**)*	490 (3.92), 530 (3.58)	8%
Flavomannin-6,6′-dimethyl ether (**6**)	407 (4.29)	2%
Emodin (**7**)	436 (4.03)	28%
7,7′-Biphyscion (**8**)*	440 (3.48)	20%

^a^In MeOH. ^b^In air-saturated CD_3_OD. ^c^Phosphorescence detection (*λ* = 1275 nm) measurement of ^1^O_2_ using Ru(bpy)_3_Cl_2_ as standard with *Φ*_∆, CD3OD_ = 0.73. Laser settings: 450 nm, 15 mW

*Data from [8]

With a quantum yield of 28%, **7** represents the most efficient PS of the isolated metabolites of *C.* *rubrophyllus*. Thus, this compound is far more active than the other monomeric anthraquinones **4** and **5** and even more efficient in generating ^1^O_2_ than compound **8**. The photophysical characteristics of the well-known natural photosensitizer emodin (**7**) have already been investigated by Gollnick et al. in 1992 [26]. In acetonitrile, a photoyield of *Φ*_Δ, acetonitrile_ = 0.82 was detected. In deuterated methanol, we recorded a photoyield of *Φ*_Δ, CD3OD_ = 0.28, which indicates that **7** forms intermolecular bonds with methanol enabling an efficient non-radiant decay of the excited state [27]. Glucosylation of **7** leads to a considerable decrease (*Δ* = 17%) in photoactivity as the quantum yield for **3** was calculated to be *Φ*_Δ, CD3OD_ = 11%. Despite the reduction of activity, **3** still outranks compounds **4–6**, which clearly highlights the neglected potential of glycosylated anthraquinones for various photodynamic applications. Furthermore, this is the first report of a photoactive glycosylated AQ so far. The biosynthetic precursor (i.e., compound **6**) [28] of the highly active dimeric anthraquinone **8** exhibited a minor quantum yield of 2%. Thus, the conversion of **6** into **8** via oxidative processes leads to a dramatic increase in photoactivity (*Φ*_Δ, CD3OD_ = 2 vs. 20%). Since **8** is present only in small amounts in fresh extracts of *C.* *rubrophyllus* (Fig. 1 and SI: Chapter 2.1.1, Table S3), the observed generation of ^1^O_2_ originates mainly from **7** and its glucose-substituted derivative **3**.

### Photobiological evaluation

**Fig. 3:**
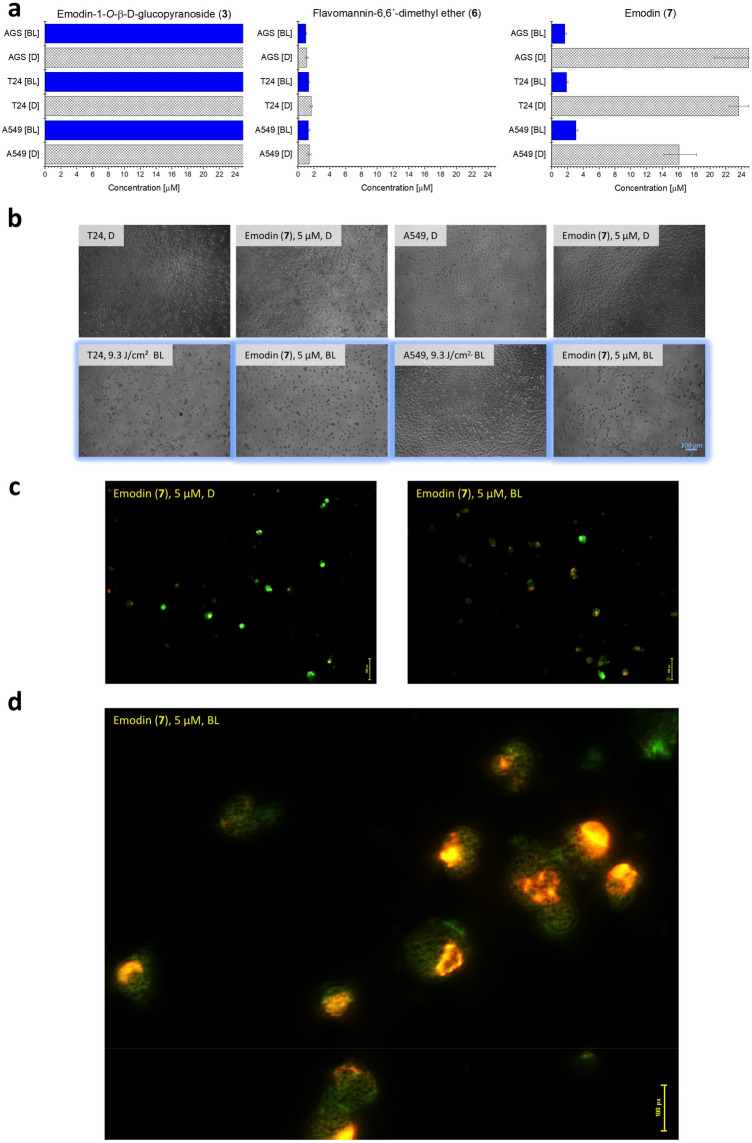
**a** (Photo)cytotoxic activity of the isolated compounds **3**, **6**, and **7** against AGS, T24, and A549 cancer cells in the presence (BL/Blue light, *λ* = 468 ± 27 nm, *H* = 9.3 J cm^−2^) and in the absence of blue light (D/Dark). Bars: EC_50_ value in µM with the respective confidence interval (95%). The open bar indicates that the true cytotoxic values lie above 25 µM, the highest concentration tested. **b** Micrographs (100 × magnification) of cells of the T24 urinary bladder carcinoma cell line as well as of the A549 non-small lung cancer cell line treated (24 h) with emodin (**7**). The upper line of pictures shows treated cells in the dark (D), the lower after irradiation with blue light (BL, *λ* = 468 ± 27 nm, *H* = 9.3 J cm^−2^). **c**, **d** Morphology of T24 cancer cells stained with acridine orange/ethidium bromide after blue light-activated (*λ* = 468 ± 27 nm, *H* = 9.3 J cm^−2^) treatment with emodin (**7**, *c* = 5 µM) visualized using fluorescence microscopy [**c** 20 × objective, **d** 60 × objective]

To investigate the photoactivity of the isolated compounds **3**, **6**, and **7** in vitro, they were subjected to a (photo)cytotoxicity assay (Fig. 3). The toxicity of the compounds was tested in the dark and after blue light irradiation (*λ* = 468 ± 27 nm, *H* = 9.3 J cm^−2^) against cells of the three PDT-relevant cancer cell lines A549 (lung), AGS (stomach), and T24 (bladder). Irradiation alone resulted in a detectable but negligible effect. The mean viability of the untreated, irradiated cells was above 85% for all cell lines compared to the untreated, unirradiated control. Since the standard quality criteria for phototoxicity testing established by the European Agency for the Evaluation of Medicinal Products (i.e., viability must be more than 80%) were met [29, 30], the EC_50_ values determined could be attributed to the isolated photosensitizers. The relatively weak photosensitizer flavomannin-6,6′-dimethyl ether (**6**) showed prominent dark cytotoxicity against all cell lines (EC_50|A549|D_ = 1.41 + 0.23/− 0.19 µM, EC_50|AGS|D_ = 1.09 + 0.18/− 0.15 µM, EC_50|T24|D_ = 1.66 + 0.15/− 0.13 µM). Since photoindices were close to 1 for all cancer cell lines (P.I._A549_ = 1.09, P.I._AGS_ = 1.10, P.I._T24_ = 1.24), no clear phototoxic effect was observed for **6**. Pachón-Peña et al. demonstrated dose-dependent growth inhibition of **6** in Caco-2 tumor cells (IC_50_ = 96 ± 3 µg/mL after 24 h of incubation) in the absence of a genotoxic effect, additionally underlining the potential of **6** as a natural anticancer agent [31]. However, the oxidation of **6**–**8** results in an astonishing increase in photodynamic activity. As shown in our previous work, 7,7′-biphyscion (**8**) showed photocytotoxicity in the nanomolar range [8] (Table S6).

Although emodin (**7**) displayed the highest singlet oxygen quantum yield of all pigments isolated from *C.* *rubrophyllus*, its photoactivity was considerably lower than the one reported for **8**, which was active against various cancer cells in the nanomolar range after blue light irradiation (*λ* = 468 ± 27 nm, 9.3 J cm^−2^) and could induce dose-dependent apoptosis [8]. Nevertheless, **7** was active in the low micromolar range and showed good photo indices (SI: Chapter 3.1). Furthermore, the cancer cells treated with **7** and irradiated with blue light were shrunk, which could hint towards an apoptotic cell death (Fig. 3 and Figure S23-25) [32]. A dye exclusion assay indicated secondary necrosis (Fig. 3c and d), as the nuclear chromatin was condensed, but the membrane integrity was lost. Its EC_50_-values were calculated to be 3.06 + 0.26/− 0.24 µM, 1.68 + 0.15/− 0.14 µM, and 1.89 + 0.16/− 0.15 µM for A549, AGS, and T24 cells, respectively, when combined with blue light irradiation (*λ* = 468 ± 27 nm, *H* = 9.3 J cm^−2^). The monomeric anthraquinone emodin is known to induce red blood cell lysis and lipid peroxidation after irradiation, with reactions of radical species, reactive oxygen intermediates, and stable photoproducts with cellular components being the most likely mechanisms of action [33]. The compound was also photocytotoxic on Chinese hamster V79 cells by Kersten et al. without being photogenotoxic, as no micronuclei were induced [34]. However, comprehensive EC_50_ values of **7** (Table S6) are reported here for the first time.

Despite demonstrating photophysical activity (Table 1), emodin-1-*O*-β-D-glucopyranoside (**3**) failed to induce photocytotoxicity in the tested concentration range (EC_50_ > 25 µM). The reason for that could come from its physicochemical properties, which are significantly altered due to glucosylation at position 1 compared to its aglycone emodin (**7**). Photosensitizers used for the photodynamic treatment of tumors are mostly lipophilic compounds that rapidly diffuse into cancer cells and accumulate in intracellular membrane structures (e.g., mitochondria and the endoplasmatic reticulum (ER)) [35]. In their study on the potential of the natural AQ parietin for the photodynamic treatment of cancer, Mugas et al. showed that the compound is rapidly internalized in K562 cells mainly via passive diffusion [36]. Moreover, the involvement of both passive and carrier-mediated processes [e.g., P-glycoprotein, multidrug-resistant proteins (MRP) and sodium-glucose cotransporter (SGLT1)] in the cellular absorption of emodin and chrysophanol has been demonstrated in human intestinal Caco-2 cells [37]. Thus, passive diffusion seems to be the most probable absorption pathway for AQs. The sugar moiety of **3** strongly increases its hydrophilicity, which is likely to hinder the passive crossing of cell membranes. However, the exact uptake mechanisms of glycosylated AQs as well as their photodynamic potential are still unexplored and therefore require further investigation.

### Towards an optimized 7,7′-biphyscion (8) isolation workflow

Since **8** exhibited promising photoactivated cytotoxicity (SI: Chapter 3.1, Table S6), obtaining larger quantities of the pure compound was of high priority. A standard protocol for the isolation of 7,7′-biphyscion (**8**) has been presented in our recent work on the photoactive secondary metabolites of *C.* *uliginosus* [8]. Due to the low yield of **8** in the biomaterial, several kilograms of fruiting bodies need to be collected and identified, which is time-consuming in itself and often hindered by the sporadic occurrence and unique specific habitats of some species [38]. Nevertheless, its precursor flavomannin-6,6′-dimethyl ether (**6**) occurs in larger quantities and in several species of the subgenus dermocyboid C. [39]. Studying *C. rubrophyllus* extracts for multiple months has shown that **6** gradually decomposes and **8** is formed (data not shown). Thus, the content of **8** strongly depends on the age and oxidation degree of the biomaterial or the extracts used for mycochemical analysis.

To maximize the yield of **8**, a new isolation protocol was designed that takes advantage of the oxidation sensitivity of **6**. In a first step, the dichloromethane extract was refluxed with a mixture of acetic acid and hydrochloric acid to dehydrate **6** to its anhydrous derivatives. Subsequently, the dried extract was warmed with sodium hydroxide solution in the presence of hydrogen peroxide to generate **8** by oxidative processes. After acidification of the reaction solution with acetic acid and extraction with diethyl ether, an extract rich in **8** was obtained without its precursor compound **6**. Then, dry column vacuum chromatography was used for the isolation of **8**. The application of the novel workflow results in a high yield of **8** per mass of starting crude extract (*η* = 4.8%) even when working with a freshly prepared extract (SI: Figure S26).

To validate our new isolation strategy, we submitted another dermocyboid C., i.e. the yellow *C.* *holoxanthus*, to the workflow. A mycochemical analysis of *C.* *holoxanthus* based on HPLC–MS is given in the electronic supplementary material (SI: Chapter 4.1). First, extracts of different polarities (i.e., petroleum ether, dichloromethane, and methanol) were prepared from the dried and ground fruiting bodies (Figure S27). HPLC–DAD-MS analysis and subsequent metabolite annotation via dereplication (SI: Chapter 4.1.3, Figure S28, Table S8) (i.e., comparison of spectral data – MS and UV/Vis) confirmed the presence of **6** in *C.* *holoxanthus*, which was the basic requirement. Thus, the FDM-rich dichloromethane extract of *C.* *holoxanthus* (SI: Figure S29) was treated as described above to enrich **8**. Then, **8** was isolated by preparative thin-layer chromatography (TLC) and identified via HPLC–DAD-MS analysis and ^1^H NMR spectroscopy. In comparison to dry vacuum column chromatography, preparative TLC proved to be an equally useful and simple approach. It is particularly advantageous when small quantities of extracts/fractions are separated. In sum, following this procedure, the yield of **8** was increased by 100% as compared to the maximal theoretical content of **8** in the unoxidized DCM extract.

**Fig. 4:**
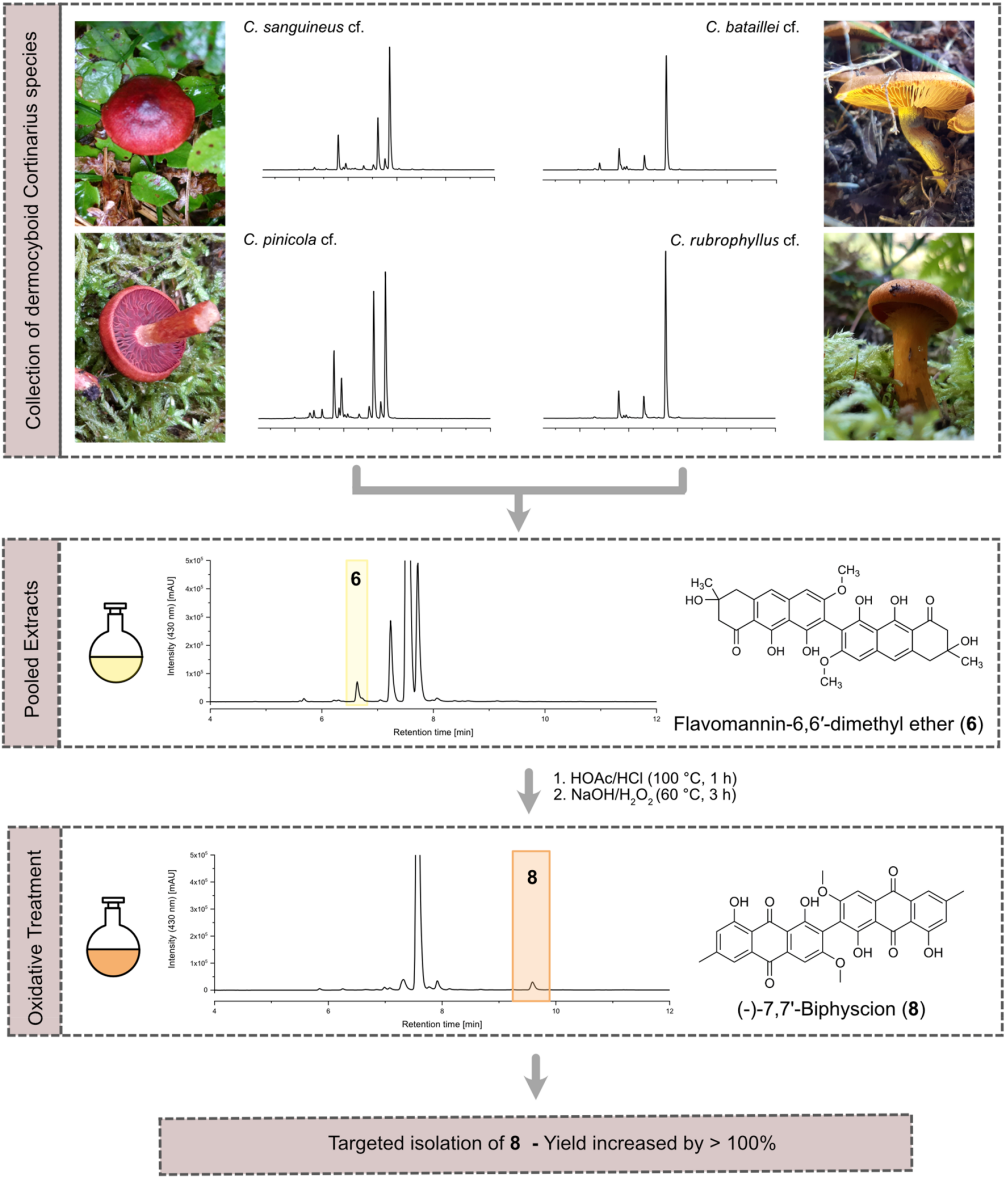
Schematic workflow of the optimized isolation procedure of **8** from different species of colourful dermocyboid C. After pooling and oxidative treatment of the extracts, **8** is significantly enriched and the matrix complexity reduced

In a next step, we wanted to test our hypothesis that **8** can be conveniently isolated from a mixture of different *dermocyboid Cortinarius* species. While the identification at the species level is arduous and not easily accomplished without ITS sequencing, the general identification of dermocyboid C. is straightforward due to their brightly colored lamellae (see Fig. 4). Thus, we started with five preliminarily identified dermocyboid C. (i.e., *Cortinarius pinicola* cf., *C. sanguineus* cf., *C. semisanguineus* cf., *C. bataillei* cf., and *C. rubrophyllus* cf.), pooled their extracts, and submitted them to the dehydration and oxidation step. As depicted in Fig. 4, the extract was significantly enriched with **8**, and the complexity of the matrix was reduced due to the instability of monomeric AQs in the acidic and oxidative environment [40]. As the last step, dry column vacuum chromatography and or preparative TLC resulted in quantitative amounts of **8**.

In sum, starting from three different biological sources, we proved that **8** can be enriched in the crude extract and thus the isolation yield can be greatly increased. While using a mélange of various colorful dermocyboid fruiting bodies for separation, we showed that the limitations of complicated fungal identification and restricted amounts of available biomaterial of single species can be circumvented. The rapid isolation of **8** from different colorful ECM fungi such as *Phlegmacium*, *Dermocybe*, and *Tricholoma* species [28] together could thus be achieved productively and efficiently as long as they contain at least **6**.

## Conclusion

In the presented study, seven AQs (**1–5** and **7–8**) and one pre-AQ (**6**) were isolated from the fruiting bodies of *Cortinarius rubrophyllus* (Sect. *Dermocybe*). Two of them, **1** (emodin-1,6-di-*O*-β-D-glucopyranoside) and **2** (dermolutein-6-*O*-β-D-glucopyranoside), were identified as new natural products. Furthermore, compounds **3**, **6**, and **7** were photophysically characterized, and their (photo)cytotoxicity was evaluated. Emodin (**7**) demonstrated EC_50_-values in the low micromolar range against three cancer cell lines when combined with blue light irradiation. In addition, a method to enrich 7,7′-biphyscion (**8**) in crude extracts of FDM-containing species (e.g., *Cortinarius* ssp. and *Tricholoma* ssp.) was presented. This protocol allows to circumvent the problem of limited amounts of biomaterial and thus paves the way towards extended photopharmaceutical studies.

## Supplementary Information

Below is the link to the electronic supplementary material.

Supplementary file1 (DOCX 4535 kb)

## Abbreviations

AQ: Anthraquinone
ECM: Ectomycorrhizal
EC50: Half maximal effective concentration
Dermocyboid C.: Dermocyboid Cortinarii
PDT: Photodynamic therapy
SI: Supplementary information
TLC: Thin layer chromatography

## References

[1] Garnica, S., Weiss, M., Oertel, B., & Oberwinkler, F. (2005). A framework for a phylogenetic classification in the genus Cortinarius (Basidiomycota, Agaricales) derived from morphological and molecular data. *Canadian Journal of Botany*, 83(11), 1457–1477. Doi: 10.1139/b05-107.

[2] Taylor AFS, Alexander IAN (2005). The ectomycorrhizal symbiosis: Life in the real world. Mycologist.

[3] Dominguez-Nunez, J. A., & Albanesi, A. S. 2021. Ectomycorrhizal fungi as biofertilizers in forestry. In, 2020 (pp. 1–18) American Chemical Society (ACS), IntechOpen Ltd. Doi:10.5772/intechopen.88585

[4] Splivallo R, Fischer U, Göbel C, Feussner I, Karlovsky P (2009). Truffles regulate plant root morphogenesis via the production of auxin and ethylene. Plant Physiology.

[5] Spiteller P (2015). Chemical ecology of fungi. Natural Product Reports.

[6] Siewert B, Vrabl P, Hammerle F, Bingger I, Stuppner H (2019). A convenient workflow to spot photosensitizers revealed photo-activity in basidiomycetes. RSC Advances.

[7] Hammerle F, Quirós-Guerrero L, Wolfender J-L, Peintner U, Siewert B (2021). Feature-based molecular networking—an exciting tool to spot hidden photosensitizers in the genus cortinarius?. Photochemical and Photobiological Sciences.

[8] Fabian, H., Isabella, B., Andrea, P., Alexander, M., Ronald, G., & Adriano, R., et al. (2021). Targeted Isolation of Photoactive Pigments from Mushrooms Yielded a Highly Potent New Photosensitizer: 7,7’-Biphyscion 10.1038/s41598-022-04975-9 PMC8782903 35064132

[9] Fiala J, Schöbel H, Vrabl P, Dietrich D, Hammerle F, Artmann DJ (2021). A new high-throughput-screening-assay for photoantimicrobials based on EUCAST revealed unknown photoantimicrobials in cortinariaceae. Frontiers in Microbiology.

[10] Hopkins SL, Siewert B, Askes SHC, Veldhuizen P, Zwier R, Heger M (2016). An in vitro cell irradiation protocol for testing photopharmaceuticals and the effect of blue, green, and red light on human cancer cell lines. Photochemical and Photobiological Sciences.

[11] Zhou X-Q, Busemann A, Meijer MS, Siegler MA, Bonnet S (2019). The two isomers of a cyclometallated palladium sensitizer show different photodynamic properties in cancer cells. Chemical Communications.

[12] DeRosa MC, Crutchley RJ (2002). Photosensitized singlet oxygen and its applications. Coordination Chemistry Reviews.

[13] Barqawi KR, Akasheh TS, Beaumont PC, Parsons BJ, Phillips GO (1988). The luminescent charge-transfer state of ruthenium-bipyrazine complexes. The Journal of Physical Chemistry.

[14] McGahon AJ, Martin SJ, Bissonnette RP, Mahboubi A, Shi Y, Mogil RJ, Schwartz LM, Osborne BA (1995). Chapter 9 The end of the (cell) line: methods for the study of apoptosis in vitro. Methods in cell biology.

[15] Keller, G. (1982). Pigmentationsuntersuchungen bei europäischen Arten aus der Gattung Dermocybe (FR.) WÜNSCHE, pp. 110–126

[16] Lösel, W. (1968). Untersuchungen über die anthrachinonpigmente von dermocybe sanguinea (wulf. ex fr.) wünsche und verwandter arten. Technische Hochschule München

[17] Uddin Z, Song YH, Curtis-Long MJ, Kim JY, Yuk HJ, Park KH (2016). Potent bacterial neuraminidase inhibitors, anthraquinone glucosides from Polygonum cuspidatum and their inhibitory mechanism. Journal of Ethnopharmacology.

[18] Oertel, B. (1984). *Untersuchungen zur konstitution von dihydroanthracenonen und angaben zu ihrer verbreitung in pilzen*. Friedrich-Wilhelms-Universität Bonn

[19] Danielsen K, Aksnes DW, Francis GW (1992). NMR study of some anthraquinones from rhubarb. Magnetic Resonance in Chemistry.

[20] da Silva Brandão M, Silva Abreu L, Geris R (2019). Phialomyces macrosporus: chemical constituents, antimicrobial activity and complete NMR assignments for the 7,7′-biphyscion. Chemistry and Biodiversity.

[21] Soop K, Dima B, Cooper JA, Park D, Oertel B (2019). A phylogenetic approach to a global supraspecific taxonomy of Cortinarius (Agaricales) with an emphasis on the southern mycota. Persoonia Molecular Phylogeny and Evolution of Fungi.

[22] Räisänen R (2019). Fungal colorants in applications focus on *Cortinarius species*. Coloration Technology.

[23] Räisänen R, Primetta A, Nikunen S, Honkalampi U, Nygren H, Pihlava J-M (2020). Examining safety of biocolourants from fungal and plant sources-examples from cortinarius and tapinella, salix and *Tanacetum* spp and Dyed Woollen Fabrics. Antibiotics.

[24] Steglich, W., Lösel, W., & Austel, V. (1969). Pilzpigmente, IV. Anthrachinon-pigmente aus dermocybe sanguinea (Wulf. ex Fr.) Wünsche und D. semisanguinea (Fr.). *Chemische Berichte, 102*(12), 4104–4118. Doi:10.1002/cber.19691021217.

[25] Siewert, B., Curak, G., Hammerle, F., Huymann, L., Fiala, J., & Peintner, U. (2021). The Photocytotoxicity of the fungus *Cortinarius rubrophyllus* is concentrated in its gills. *Journal of Photochemistry and Photobiology B: Biology*10.1016/j.jphotobiol.2022.112390 35123160

[26] Gollnick K, Held S, Mártire DO, Braslavsky SE (1992). Hydroxyanthraquinones as sensitizers of singlet oxygen reactions: Quantum yields of triplet formation and singlet oxygen generation in acetonitrile. Journal of Photochemistry and Photobiology A Chemistry.

[27] Bellissima S, De Panfilis S, Bafile U, Cunsolo A, González MA, Guarini E (2016). The hydrogen-bond collective dynamics in liquid methanol. Scientific Reports.

[28] Gill M, Steglich W (1987). Pigments of fungi (Macromycetes). Fortschritte der Chemie Organischer Naturstoffe.

[29] CPMP (2002). Note for guidance on photosafety testing. In: EMEA (Ed.), (Vol. CPMP/SWP/398/01). London

[30] OECD (2004). OECD Guideline for testing of Chemicals. In: vitro 3T3 NRU phototoxicity test. In OCDE (Ed.), 432

[31] Pachon-Pena G, Reyes-Zurita FJ, Deffieux G, Azqueta A, de Centelles JJ (2009). Antiproliferative effect of flavomannin-6,6'-dimethylether from Tricholoma equestre on Caco-2 cells. Toxicology.

[32] Saraste A, Pulkki K (2000). Morphologic and biochemical hallmarks of apoptosis. Cardiovascular Research.

[33] Vargas F, Fraile G, Velásquez M, Correia H, Fonseca G, Marín M (2002). Studies on the photostability and phototoxicity of aloe-emodin, emodin and rhein. Die Pharmazie.

[34] Kersten B, Zhang J, Brendler-Schwaab SY, Kasper P, Müller L (1999). The application of the micronucleus test in Chinese hamster V79 cells to detect drug-induced photogenotoxicity. Mutation Research/Genetic Toxicology and Environmental Mutagenesis.

[35] Abrahamse H, Hamblin MR (2016). New photosensitizers for photodynamic therapy. The Biochemical Journal.

[36] Mugas ML, Calvo G, Marioni J, Cespedes M, Martinez F, Saenz D (2021). Photodynamic therapy of tumour cells mediated by the natural anthraquinone parietin and blue light. Journal of Photochemistry and Photobiology B.

[37] Teng Z-H, Zhou S-Y, Ran Y-H, Liu X-Y, Yang R-T, Yang X (2007). Cellular absorption of anthraquinones emodin and chrysophanol in human intestinal Caco-2 cells. Bioscience Biotechnology and Biochemistry.

[38] Garnica S, Schoen ME, Abarenkov K, Riess K, Liimatainen K, Niskanen T (2016). Determining threshold values for barcoding fungi: lessons from Cortinarius (Basidiomycota), a highly diverse and widespread ectomycorrhizal genus. FEMS Microbiology Ecology.

[39] Steglich W, Toepfer-Petersen E, Reininger W, Gluchoff K, Arpin N (1972). Chemotaxonomic studies on mushrooms. XX. Pigments of Fungi. VIII. Isolation of flavomannin-6,6'-dimethyl ether and one of its racemates from higher Fungi. Phytochemistry.

[40] Narayanan S, Jadhav AP, Kadam VJ (2015). Forced degradation studies of aloe emodin and emodin by HPTLC. Indian Journal of Pharmaceutical Sciences.

