# Supplementary material for: Optimized isolation of 7,7′-biphyscion starting from Cortinarius rubrophyllus, a chemically unexplored fungal species rich in photosensitizers
Source: Photochem Photobiol Sci. 2021 Dec 31;21(2):221–34. doi: 10.1007/s43630-021-00159-y (PMC8863709; doi:10.1007/s43630-021-00159-y)
Supplement: Supplementary file 1 — Supplementary file1 (DOCX 4535 kb) [file 43630_2021_159_MOESM1_ESM.docx]

Optimized Isolation of 7,7’-Biphyscion Starting from *Cortinarius rubrophyllus*, a Chemically Unexplored Fungal Species Rich in Photosensitizers

Fabian Hammerle^a^, Lisa-Maria Steger^a^, Xuequan Zhou^b^, Sylvestre Bonnet^b^, Lesley Huymann^c^, Ursula Peintner^c^, and Bianka Siewert^a^*

*a. Mag.pharm. F. J. Hammerle, Mag.pharm. L.-M. Steger, Dr. B. Siewert, University of Innsbruck, Pharmacology and Pharmacognosy, Center for Molecular Biosciences Innsbruck,* *Innrain 80/82, 6020 Innsbruck (Austria).
E-mail address:* [*bianka.siewert@uibk.ac.at*](mailto:bianka.siewert@uibk.ac.at)

*b. M.Sc. X. Zhou, Prof. S. Bonnet, Leiden Institute of Chemistry, Leiden University, Gorlaeus Laboratories, P.O Box 9502, 2300 RA Leiden (The Netherlands).*

*c. L. Huymann, B.Sc., Prof. Dr. U. Peintner, University of Innsbruck, Microbiology, Technikerstraße 25, 6020 Innsbruck (Austria).*

* *Corresponding author.*
† Electronic Supplementary Information (SI) available: Mycochemical investigation, spectroscopic data, and (photo)cytotoxicity evaluation.

**Abstract:** Mushrooms such as the dermocyboid *Cortinarius rubrophyllus* are characterized by strikingly colourful fruiting bodies. Recently, the molecular dyes responsible for such colours experienced a comeback as photoactive compounds with remarkable photophysical and photobiological properties. One of them –7,7’-biphyscion– is a dimeric anthraquinone that showed promising anticancer effects in the low nanomolar range under blue-light irradiation. Compared to acidic anthraquinones, 7,7’-biphyscion was more efficiently taken up by cells and induced apoptosis after photoactivation. However, seasonal collection of mushrooms producing this compound, low extraction yields, and tricky fungal identification hamper further developments to the clinics. To bypass these limitations, we demonstrate here an alternative approach utilizing a precursor of 7,7’-biphyscion, i.e. the pre-anthraquinone flavomannin-6,6’-dimethyl ether, which is abundant in many species of the subgenus *Dermocybe.* A controlled oxidation of the crude extract significantly increased the yield of 7,7´-biphyscion by 100%, which eased the isolation process. We also present the mycochemical and photobiological characterization of the yet chemically undescribed species, i.e. *C. rubrophyllus*. In total, eight pigments (**1**-**8**) were isolated, including two new glycosylated anthraquinones (**1** and **2**). Light-dependent generation of singlet oxygen was detected for the first time for emodin-1-*O*-β-D-glucopyranoside (**3**) (photophysical measurement: Φ_Δ_ = 0.11 (CD_3_OD)). Furthermore, emodin (**7**) was characterized as promising compound in the photocytotoxicity assay with EC_50_-values in the low micromolar range under irradiation against cells of the cancer cell lines AGS, A549, and T24.

**Electronic supplementary information (SI)**

Table of contents

[1 Fungal biomaterial: origin and identification 3](#_Toc88598911)

[1.1 DNA-extraction and ITS-sequencing 3](#_Toc88598912)

[1.2 Yields (extracts, fractions) 3](#_Toc88598913)

[2 Mycochemistry 4](#_Toc88598914)

[2.1 Pigment profile of *Cortinarius rubrophyllus* 4](#_Toc88598915)

[2.1.1 HPLC-DAD-MS analysis 4](#_Toc88598916)

[2.1.2 Isolation of secondary metabolites (compounds **1-7**) 4](#_Toc88598917)

[2.1.3 Chemical characterization of isolated secondary metabolites (compounds **1-8**) 6](#_Toc88598918)

[2.1.4 Spectral data: IR 10](#_Toc88598919)

[2.1.5 Spectral data: UV/Vis 11](#_Toc88598920)

[2.1.6 Spectral data: NMR 14](#_Toc88598921)

[2.2 GC-MS analysis of the *C. rubrophyllus* methanol extract 20](#_Toc88598922)

[3 Biological evaluation – (Photo)cytotoxicity assay 21](#_Toc88598923)

[3.1 EC_50_-values 21](#_Toc88598924)

[3.2 Micrographs 22](#_Toc88598925)

[4 On the isolation of compound **8** 23](#_Toc88598926)

[4.1 Mycochemical analysis of *Cortinarius holoxanthus* 23](#_Toc88598927)

[4.1.1 Extract preparation 23](#_Toc88598928)

[4.1.2 HPLC-DAD analysis 24](#_Toc88598929)

[4.1.3 Secondary metabolite annotation 25](#_Toc88598930)

[4.1.4 *Cortinarius holoxanthus* – Isolation of 7,7´-biphyscion (**8**) 26](#_Toc88598931)

[4.1.5 HPLC-DAD-MS analysis 27](#_Toc88598932)

[5 References 29](#_Toc88598933)

# Fungal biomaterial: origin and identification

**Table S1.** Cortinarius collections used in this study with respective voucher numbers and collection data. The material is deposited in the the mycological collection of the Tiroler Landesmuseen (IBF). (* … identification based solely on macroscopic characteristics)

| Cortinarius | Authority | Subgenus / Section | Voucher/  GenBank | Collection date | Origin |
| --- | --- | --- | --- | --- | --- |
| *C. rubrophyllus* | (Moënne-Locc.) Liimat., Niskanen, Ammirati & Dima | Dermocybe / Sect. Dermocybe | IBF20190002a  GenBank Acc.  MZ357345 | August-September 2019 | Austria, Tyrol, Innsbruck |
| *C. holoxanthus* | (M.M. Moser & I. Gruber) Nezdojm., Novosti Sistematiki Nizshikh Rastenii |  | IBF20200030  GenBank Acc.  MW880252 | 08.09.2020 | Austria, Tyrol, Lans |
| *C. bataillei** |  |  | - | 14.09.2019 | Austria, Tyrol, Lans |
| *C. pinicola* |  |  | IBF20190004 | 22.08.2019 | Austria, Tyrol, Mutters |
| *C. rubrophyllus* |  |  | IBF20190002 | 05.09.2019 | Austria, Tyrol, Lans |
| *C. sanguineus** |  |  | - | 28.08.2019 | Austria, Tyrol, Mutters |
| *C. semisanguineus** |  |  | - | 05.09.2019 | Austria, Tyrol, Lans |

*Assigned only based on macroscopic characteristics.

IBF is the official herbarium code (<http://sweetgum.nybg.org/science/ih/herbarium-details/?irn=126158>, accessed 24.11.2021).

## DNA-extraction and ITS-sequencing

Taxonomic classification by ITS-sequence analysis was performed as published elsewhere [1]. Briefly, after DNA-extraction, the ITS-region was amplified by polymerase chain reaction (PCR). The amplified and purified PCR products were sent to Microsynth AG® (Balgach, Switzerland), where the samples got sequenced by sanger sequencing.

## Yields (extracts, fractions)

**Table S2.** Cortinarius rubrophyllus – Extract yields as well as the fraction yields, which resulted from the liquid-liquid fractionation of the methanolic extract.

| **Ultra-sonication** | **Mass biomaterial [g]** | **Solvent** | **Yield [mg (%dw)]** |
| --- | --- | --- | --- |
| *Cortinarius rubrophyllus* | 42.0 | Petroleum ether | 500.0 (1.19%) |
|  |  | Dichloromethane | 724.2 (1.72%) |
|  |  | Methanol | 9082.8 (21.63%) |
| **Liquid-liquid fractionation** | **Mass methanolic extract [mg]** | **Solvent** | **Yield [mg (%dw)]** |
| MeOH Extract *C. rubrophyllus* | 2012.7 | Diethyl ether | 749.9 (37.3%) |
|  |  | Ethyl acetate | 231.3 (8.5%) |
|  |  | Water | 1031.5 (51.2%) |

# Mycochemistry

## Pigment profile of *Cortinarius rubrophyllus*

The pigment profile of *Cortinarius rubrophyllus* was investigated using standard mychochemical techniques such as high-performance liquid chromatography in combination with various detection methods (i.e., DAD and MS) and thin-layer chromatography.

### HPLC-DAD-MS analysis

**Table S3.** List of minor and major compounds with their respective retention times and molecular masses (HPLC-DAD-MS experiment: negative ionisation mode).

| **Name** | **Compound #** | **Retention time [min]** | **[M-H]^-^** | **Extract** | **Occurrence [minor … m, major … M]** |
| --- | --- | --- | --- | --- | --- |
| Emodin-1,6-di-*O*-β-D-glucopyranoside | **1** | 3.82 | 593.0 | MeOH | m |
| Dermolutein-6-*O*-β-D-glucopyranoside | **2** | 4.25 | 489.0 | MeOH | m |
| Emodin-1-*O*-β-D-glucopyranoside | **3** | 4.69 | 431.0 | MeOH | M |
| Dermolutein | **4** | 5.49 | 326.9 | MeOH | m |
| Dermorubin | **5** | 5.69 | 342.9 | MeOH | m |
| Flavomannin-6,6´-dimethyl ether | **6** | 6.46 | 573.0 | MeOH, DCM | m, M |
| Emodin | **7** | 7.30 | 268.9 | MeOH, DCM, PE | m, M, M |
| 7,7´-Biphyscion | **8** | 9.30 | 565.2 | MeOH, DCM, PE | m, m, m |

### Isolation of secondary metabolites (compounds **1-7**)

#### Isolation of compounds 1 and 2

To enrich compounds **1** and **2** in the water fraction (L3), it was submitted to RP-18 solid-phase extraction (stationary phase: LiChroprep® RP-18 (0.040-0.063 mm), $\emptyset$ = 15 mm, l = 25 mm). After activation of the column with methanol, it was equilibrated with water and loaded with the dissolved water fraction (approximately 200 mg in 2 mL water). The loaded column was washed with 10 mL of water and then elution was performed with MeOH-H_2_O (9:1 v/v). The resulting fraction was dried under an air-stream at room temperature. Three aliquots of the water fraction were weighed in (m_1_ = 288.1 mg, m_2_ = 218.0 mg, and m_3_ = 226.1 mg) and separately submitted to RP-18 SPE, yielding 51.0 mg of combined enriched fraction (H1). The entirety of the resulting fraction was subjected to Sephadex LH-20 column chromatography ($\emptyset$ = 2.5 cm, l = 18 cm) using isocratic elution with methanol to afford 24 fractions (V = 5 mL each). After monitoring the separation by TLC (mobile phase: toluene/ethyl acetate/formic acid/acetic acid = 60:30:5:5), fractions 9-22 were combined (H1.1 / η = 29.6 mg). Faction H1.1 was further separated via liquid-liquid fractionation. It was dissolved in water (V = 30 mL) and transferred into a separating funnel. After extracting the solution with ethyl acetate (V = 30 mL, n = 6, H1.1.1), the water fraction was acidified with approx. 4 mL of acetic acid, and again extracted with ethyl acetate (V = 30 mL, n = 2, H1.1.2). The ethyl acetate fractions were evaporated to dryness and yielded 3.0 mg for H1.1.1 and 6.6 mg for H1.1.2. The remaining acidified water phase was submitted to RP-18 SPE as described above, yielding 16.6 mg of dried fraction (H1.1.3). H1.1.3 (m = 16.6 mg) was purified by preparative TLC (pre-coated TLC sheets, 10 x 20 cm, silica gel 60 F254 0.20 mm layer) with toluene/acetone/formic acid/acetic acid (35:40:12.5:12.5) as mobile phase. For this purpose, H1.1.3 was dissolved in methanol (V $\sim$ 3 ml) and loaded onto 12 TLC plates. Every plate was developed once (separation distance $\sim$ 8 cm). The corresponding areas (**1**: R_f_ = 0.2, **2**: R_f_ = 0.5) were removed, suspended via sonication in water, and purified via RP-18 SPE as described above, whereby elution of compound **1** (2.2 mg) was achieved with acetonitrile and elution of compound **2** (1.5 mg) with acetone. Chromatograms (λ = 430 nm) of the HPLD-DAD-MS analysis of **1** and **2** are shown in Figure S1.

**Figure S1.** Results of the HPLC-DAD-MS analysis of compounds **1** and **2**, which were isolated from the C. rubrophyllus methanol extract via preparative TLC. The chromatograms of **1**, **2**, and the extract were recorded at λ = 430 nm. All samples were dissolved in DMSO prior to analysis. Stationary Phase: Phenomenex Synergi MAX-RP, Mobile Phase: H2O/ACN + 0.1% FA, ratio of solvent B (% ACN + 0.1% FA) is displayed in the top chromatogram by the blue line.

#### Isolation of compounds **3**, **4** and **5**

An aliquot (m = 20.5 mg) of the ethyl acetate fraction (L2) was subjected to acetylated polyamide column chromatography ($\emptyset$ = 1 cm, l = 18 cm). First, isocratic elution was performed with dichloromethane/acetone (66:33), followed by isocratic elution with ethanol 96%. The latter elution step yielded 28 fractions (V = 5 ml each), which were monitored via TLC (mobile phase: toluene/ethyl acetate/formic acid/acetic acid = 60:30:5:5). Thus, fractions 1-7 (E1: η = 14.9 mg) and 8-28 (E2: η = 4.4 mg) were combined. Fraction E1 was further purified by repeatedly suspending it in ethyl acetate, filtrating the suspension through cotton wool, and dissolving the filtrate cake in methanol. Repeated purification of E1 yielded 12.0 mg of compound **3**. HPLC-DAD-MS and TLC analyses of E2 showed that the fraction was a mixture of compounds **4** and **5**.

#### Isolation of compounds **6** and **7**

An aliquot (m = 50.0 mg) of the dichloromethane extract was separated by acetylated polyamide column chromatography ($\emptyset$ = 1 cm, l = 18 cm) using gradient elution with toluene/chloroform (100:0 – fractions 1-10, 50:50 – fractions 11-31, 0:100 – fractions 32-50). Thus, 50 fractions of approx. 5 ml each were obtained. The fractions were monitored by TLC (stationary phase: SiO_2_, mobile phase: toluene/ethyl acetate/formic acid/acetic acid = 60:30:5:5) and combined to afford 8 fractions (D1-D8). The fractions D1-D8 were submitted to HPLC-DAD-MS analysis, of which D5 and D7 were used for further purification steps. Fraction D5 (m = 9.8 mg) was subjected to Sephadex LH-20 column chromatography ($\emptyset$ = 2.5 cm, l = 18 cm) employing isocratic elution with dichloromethane/acetone (85:15) to afford 12 subfractions (D5.1-D5.12) of approx. 5 ml each. Subfraction D5.5 was subsequently separated by acetylated polyamide column chromatography ($\emptyset$ = 1 cm, l = 18 cm) with toluene/chloroform (80:20 – fractions 1-13, 0:100 – fractions 14-17) as mobile phase. Thus, 17 subfractions of approx. 5 ml each were obtained, of which fractions 3-7 were combined to yield 7.4 mg of compound **6**. Fraction D7 (m = 5.8 mg) was subjected to Sephadex LH-20 column chromatography ($\emptyset$ = 2.5 cm, l = 18 cm) employing isocratic elution with dichloromethane/acetone (85:15) to afford 9 subfractions (D7.1-D7.9). Subfractions D7.7 and D7.8 were combined and the solvents were removed under reduced pressure at 40 °C to yield 2.6 mg of compound **7**.

### Chemical characterization of isolated secondary metabolites (compounds **1-8**)

#### Emodin-1,6-di-*O*-β-D-glucopyranoside (**1**)

Emodin-1,6-di-*O*-β-D-glucopyranoside (**1**) was obtained as a yellow solid (η = 2.2 mg, 0.005%) from the water fraction of the methanol extract via RP-18 SPE and preparative TLC. The isolation protocol is given in detail in chapter 2.1.2.1.

M.p.: no clear m.p. observed (decomposition > 250 °C); ${[\alpha]}_{D}^{25}$= -56 (c = 0.10 mg/mL, MeOH); R_f_ = 0.20 (stationary phase: SiO_2_, mobile phase: toluene/acetone/formic acid/acetic acid = 35:40:12.5:12.5); ^1^H NMR (600 MHz, D_2_O, 25 °C) δ = 7.19 (s, 1H, C_ar_*H*-2), 7.13 (s, 1H, C_ar_*H*-4), 6.84 (d, *J* = 2.4 Hz, 1H, C_ar_*H*-5), 6.62 (d, *J* = 2.4 Hz, 1H, C_ar_*H*-7), 5.14 (d, *J* = 7.6 Hz, 1H, C*H*-1´´), 4.99 (d, *J* = 6.8 Hz, 1H, C*H*-1´), 4.12-4.04 (m, 2H, C*H*_a_-6’ + C*H*_a_-6’’), 3.97 – 3.88 (m, 2H, C*H*_b_-6’ + C*H*_b_-6’’), 3.82 – 3.62 (m, 8H, glycosidic H), 2.31 (s, 3H, C*H*_3_-3) ppm; IR (ART): $\boldsymbol{\nu}^{\sim}$ = 3344 (w), 2924 (w), 1629 (w), 1262 (w), 1068 (w) cm^-1^; MS (ESI, negative mode 4.5 kV) m/z (%) 629.0 (100), 656.0 (85), 431.0 (86) [M_compound_ **_3_**-H]^-^, 593.0 (66) [M-H]^-^; UV-Vis (MeOH): $\boldsymbol{\lambda}_{\boldsymbol{max}}$ (ε) = 221 (4398), 264 (3551), 414 nm (1328 mol^-1^ dm^3^ cm^-1^).

#### Dermolutein-6-*O*-β-D-glucopyranoside (**2**)

Dermolutein-6-*O*-β-D-glucopyranoside (**2**) was isolated as an orange solid (η = 1.5 mg, 0.004%) from the water fraction of the methanol extract as described in chapter 2.1.2.1.

M.p.: no clear m.p. observed (decomposition at 250-300 °C); ${[\alpha]}_{D}^{25}$= -29 (c = 0.12 mg/mL, MeOH); R_f_ = 0.50 (stationary phase: SiO_2_, mobile phase: toluene/acetone/formic acid/acetic acid = 35:40:12.5:12.5); ^1^H NMR (600 MHz, D_2_O, 25 °C) δ = 8.53 (s, 1H, O*H*-1), 7.57 (s, 1H, C_ar_*H*-4), 7.38 (d, *J* = 2.4 Hz, 1H, C_ar_*H*-5), 7.06 (d, *J* = 2.4 Hz, 1H, C_ar_*H*-7), 5.34 (d, *J* = 7.7 Hz, 1H, C*H*-1´), 4.09 (dd, *J* = 12.0 ,1.6 Hz, 1H, C*H*-6_a_´), 4.01 (s, 3H, OC*H*_3_-8), 3.91 – 3.84 (m, 2H, C*H*-5´ & C*H*-6_b_´), 3.78 (dd, *J* = 9.2, 9.2 Hz, C*H*-3´), 3.72 (dd, *J* = 9.4, 7.6 Hz, 1H, C*H*-2´), 3.62 (dd, *J* = 9.2, 9.2 Hz, 1H, C*H*-4´), 2.50 (s, 3H, C*H*_3_-3) ppm; IR (ART): $\boldsymbol{\nu}^{\sim}$ = 3331 (w), 2925 (w), 1676 (w), 1597 (w), 1363 (w), 1258 (w), 1140 (w), 1073 (w) cm^-1^; MS (ESI, negative mode 4.5 kV) m/z (%) 489.0 (100) [M-H]^-^; UV-Vis (MeOH): $\boldsymbol{\lambda}_{\boldsymbol{max}}$ (ε) = 226 (4766), 271 (3101), 422 (1195 mol^-1^ dm^3^ cm^-1^).

#### Emodin-1-*O*-β-D-glucopyranoside (**3**) [CAS: 38840-23-2]

Emodin-1-*O*-β-D-glucopyranoside (**3**) was obtained as an orange solid (η = 12.0 mg, 58.5% w/w_used_ _ethyl acetate fraction_, 0.029% w/w) from the ethyl acetate fraction, which resulted from the liquid-liquid fractionation of the *C. rubrophyllus* methanol extract, via acetylated polyamide column chromatography. The detailed isolation procedure is given in chapter 2.1.2.2.

M.p.: 220-228 °C (193 °C [2], 210-211 °C [3]); R_f_ = 0.04 (stationary phase: SiO_2_, mobile phase: toluene/ethyl acetate/acetic acid/formic acid = 60:30:5:5); ^1^H NMR (600 MHz, CD_3_OD, 25 °C) δ = 8.51 (s, 1H, C_ar_O*H*-6), 7.82 (s, 1H, C_ar_*H*-4), 7.63 (s, 1H, C_ar_*H*-2), 7.14 (s, 1H, C_ar_*H*-5), 6.55 (s, 1H, C_ar_*H*-7), 5.02 (d, *J* = 7.6 Hz, 1H, C*H*-1´), 3.97 (dd, *J* = 12.1, 2.3 Hz, 1H, C*H*_a_-6´), 3.74 (dd, *J* = 12.1, 6.3 Hz, 1H, C*H*_b_-6´), 3.70 – 3.64 (dd, *J* = 9.3, 7.6 Hz, 1H, C*H*-2´), 3.58 – 3.52 (m, 2H, C*H*-3´ & C*H*-5´), 3.46 – 3.42 (dd, *J* = 9.3, 9.3 Hz, 1H, C*H*-4´), 2.50 (s, 3H, C*H*_3_-3) ppm; IR (ART) = $\boldsymbol{\nu}^{\sim}$ = 3215 (w), 2921 (w), 1619 (w), 1594 (w), 1254 (w), 1176 (w), 1057 (w), 1023 (m), 882 (m), 719 (w), 521 (w), 420 (w) cm^-1^; MS (ESI, negative mode 4.5 kV) m/z (%) 431.7 (100) [M-H]^-^; UV-Vis (MeOH): $\boldsymbol{\lambda}_{\boldsymbol{max}}$ (ε) = 212 (25970), 252 (19466), 287 (17096), 428 nm (6239 mol^-1^ dm^3^ cm^-1^).

#### Dermolutein (**4**) and Dermorubin (**5**)

A mixture of dermolutein (**4**) and dermorubin (**5**) (η = 4.4 mg, 1.9% w/w_used ethyl acetate fraction_, 0.010% w/w) was obtained from the ethyl acetate fraction by acetylated polyamide column chromatography as described in chapter 2.1.2.2. Identification of compounds **4** and **5** was achieved via HPLC-DAD-MS analysis and validated with authentic reference samples. Since we had previously isolated compounds **4** and **5** and conducted a photobiological/-chemical evaluation, we refrained from performing any further purification steps. A thorough description of the isolation/separation procedure of **4** and **5** is depicted in our recent work on the secondary metabolites of *C. uliginosus* [4].

##### Dermolutein (**4**) and Dermorubin (**5**): HPLC-DAD-MS analysis


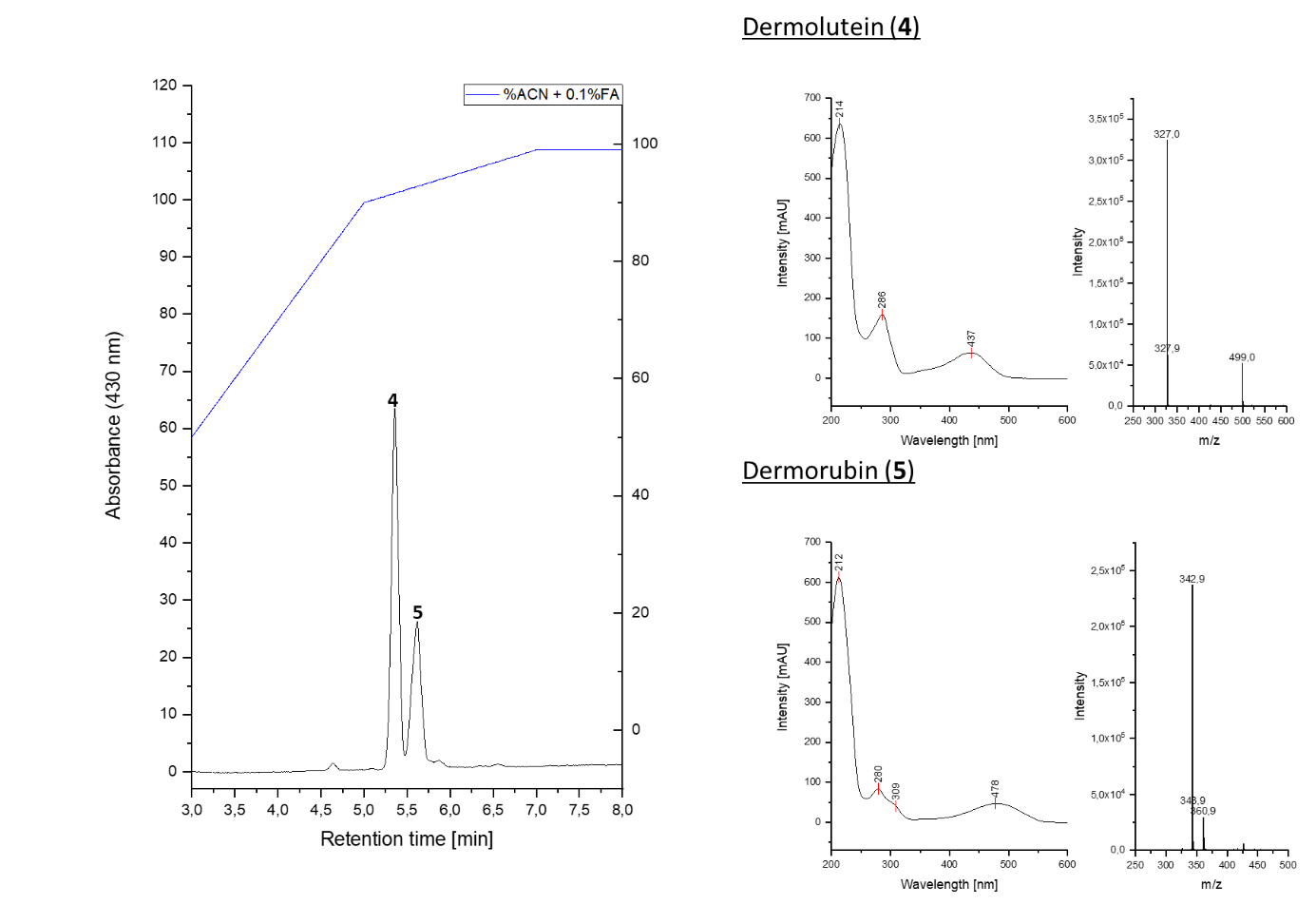


**Figure S2.** HPLC-DAD-MS analysis of the mixture containing dermolutein (4) and dermorubin (5) isolated from ethyl acetate fraction of the C. rubrophyllus methanolic extract. The mixture was dissolved in DMSO (c = 0.1 mg/mL) prior to analysis. Stationary Phase: Phenomenex Synergi MAX-RP, Mobile Phase: H_2_O (A) /ACN +0.1% FA (B), ratio of solvent B (% ACN) is displayed in the chromatogram (left side) by the blue line. The chromatogram was recorded at λ = 468 nm. On the right side, UV/Vis-spectra (200-600 nm, A/B-solvent ratio as depicted by the blue line) and mass spectra of **4** and **5** are shown.

**Table S4**. Results of the HPLC-DAD-MS analysis of the mixture containing compounds **4** and **5**, which allowed their identification as dermolutein (**4**) and dermorubin (**5**), in tabular form.

| **Name** | **Compound #** | **Retention time [min]** | **m/z** | **UV/Vis: λ_max_ in H_2_O/ACN + 0.1% FA [nm]** |
| --- | --- | --- | --- | --- |
| Dermolutein | **4** | 5.49 | 327.0 [M-H]^-^ | 214, 286, 437 |
| Dermorubin | **5** | 5.69 | 342.9 [M-H]^-^ | 212, 280, 309, 478 |

#### Flavomannin-6,6´-O-dimethyl ether (FDM) (**6**) [CAS Registry Number: 39772-00-4]

FDM (**6**) was obtained as a yellow solid (η = 7.4 mg, 14.8% w_FDM_/w_used_ _DCM extract_, 0.018% w/w) from the dichloromethane extract of *C. rubrophyllus* via acetylated polyamide column chromatography. The isolation procedure is given in chapter 2.1.2.3.

M.p.: 230-240 °C (245-250 °C [5]); R_f_ = 0.33 (stationary phase: SiO_2_, mobile phase: toluene/ethyl acetate/acetic acid/formic acid = 60:30:5:5); ^1^H NMR (600.19 MHz, CDCl_3_, 25 °C) δ = 9.99 (s, 2H, O*H*), 6.95 (s, 2H, C*H*-5 or O*H*), 6.68 (s, 2H, C*H*-5 or O*H*), 3.84 (s, 6H, O-C*H*_3_), 3.11 (d, *J* = 16.0 Hz, 2H, C*H*_a_-2), 3.05 (d, *J* = 15.9 Hz, 2H, C*H*_b_-2), 2.85 (brs, 4H, C*H*_2_-4), 1.46 (s, 6H, C*H*_3_) ppm; ^13^C (150.91 MHz, CDCl_3_, 25 °C) δ = 201.6 (C=O, C-1), 166.2 (C_ar_OH, C-9), 162.1 (C_ar_OMe), 156.7 (C_ar_OH, C-8), 140.6 (C_quart_, C-11), 117.8 (C_ar_H, C-10), 108.2 (C_quart_, C-7), 107.8 (C_quart_, C-13), 98.3 (C_ar_H, C-5), 71.1 (C_ar_OHOMe, C-3), 56.0 (C-O, OCH_­3_), 51.1 (C_ar_H, C-2), 43.4 (C_ar_H, C-4), 28.9 (CH_3_) ppm (in accordance with literature data [6, 7]); IR (ART): $\boldsymbol{\nu}^{\sim}$ = 3356 (w), 3344 (w), 2931 (w), 1618 (w), 1508 (w), 1458 (w), 1330 (w), 1094 (m), 762 (w), 532 (m) cm^-1^; MS (ESI, negative mode 4.5 kV) m/z (%) 573.0 (100) [M-H]^-^; UV-Vis (MeOH): $\boldsymbol{\lambda}_{\boldsymbol{max}}$ (ε) = 233 (29903), 279 (64669), 320 (12729), 407 nm (19296 mol^-1^ dm^3^ cm^-1^).

#### Emodin (**7**) [CAS Registry Number: 518-82-1]

Emodin (**7**) was isolated as an orange solid (η = 2.6 mg, 5.2% w/w_used_ _DCM extract_, 0.006% w/w) from the dichloromethane extract of *C. rubrophyllus* via acetylated polyamide and Sephadex LH-20 column chromatography. Please refer to chapter 2.1.2.3 for the isolation procedure.

M.p.: 260 °C (253-254 °C lit.); R_f_ = 0.64 (stationary phase: SiO_2_; mobile phase: toluene/ethyl acetate/formic acid/acetic acid = 60:30:5:5); ^1^H NMR (600.19 MHz, (CD_3_)_2_SO, 25 °C) δ = 12.09 (s, 1H, C_ar_-O*H*-8), 12.02 (s, 1H, C_ar_-O*H*-1), 7.51 (d, *J* = 1.6 Hz, 1H, C_ar_*H*-4), 7.18 (d, *J* = 1.8 Hz, 1H, C_ar_*H*-2), 7.13 (d, *J* = 2.4 Hz, 1H, C_ar_*H*-5), 6.60 (d, *J* = 2.4 Hz, 1H, C_ar_*H*-7), 2.47 ppm (s, 3H, C*H*_3_-3) (in accordance with literature data [8]); MS (ESI, negative mode 4.5 kV) m/z (%) 269.0 (100) [M-H]^-^; UV-Vis (MeOH): $\boldsymbol{\lambda}_{\boldsymbol{max}}$ (ε) = 226 (16806), 287 (17167), 436 nm (10724 mol^-1^ dm^3^ cm^-1^).

#### 7,7´-Biphyscion (**8**) [CAS Registry Number: 39772-01-5]

The orange solid (η = 2.4 mg, 4.8% w_7,7´-biphyscion_/w_pre-treated DCM extract_, 0.006% w/w) was obtained dichloromethane extract of *C. rubrophyllus* via an optimized isolation protocol.

M.p.: decomposition above 350 °C (> 350 °C lit.); R_f_ = 0.75 (stationary phase: SiO_2_, mobile phase: toluene/methanol/ethyl acetate/formic acid = 94:2.5:2.5:1); ^1^H NMR (400 MHz, CDCl_3_, 25 °C) δ = 12.45 (s, 2H, O*H*), 12.09 (s, 2H, O*H*), 7.67 (dd, *J* = 1.6, 0.6 Hz, 2H, C*H*-4), 7.55 (brs, 2H, C*H*-5), 7.10 (dd, *J* = 1.8, 0.9 Hz, 2H, C*H*-2), 3.97 (s, 6H, O-C*H*_3_), 2.48 (dd, *J* = 0.7, 0.7 Hz, 6H, C*H*_3_) ppm; IR (ART): $\boldsymbol{\nu}^{\sim}$= 1673 (w) (C=O), 1619 (m) (C=C), 1598 (m) (C=C), 1558 (m) (C=C) cm^-1^; MS (ESI, negative mode 4.5 kV) m/z (%): 565.2 (100) [M-H]^-^; UV-Vis (MeOH): $\boldsymbol{\lambda}_{\boldsymbol{max}}$ (ε) = 223 (6615), 279 (5095), 439 nm (2662 mol^-1^ dm^3^ cm^-1^).

### Spectral data: IR

**Figure S3.** IR-spectrum of emodin-1,6-di-O-β-D-glucopyranoside (**1**).

**Figure S4.** IR-spectrum of dermolutein-6-O-β-D-glucopyranoside (**2**).

### Spectral data: UV/Vis


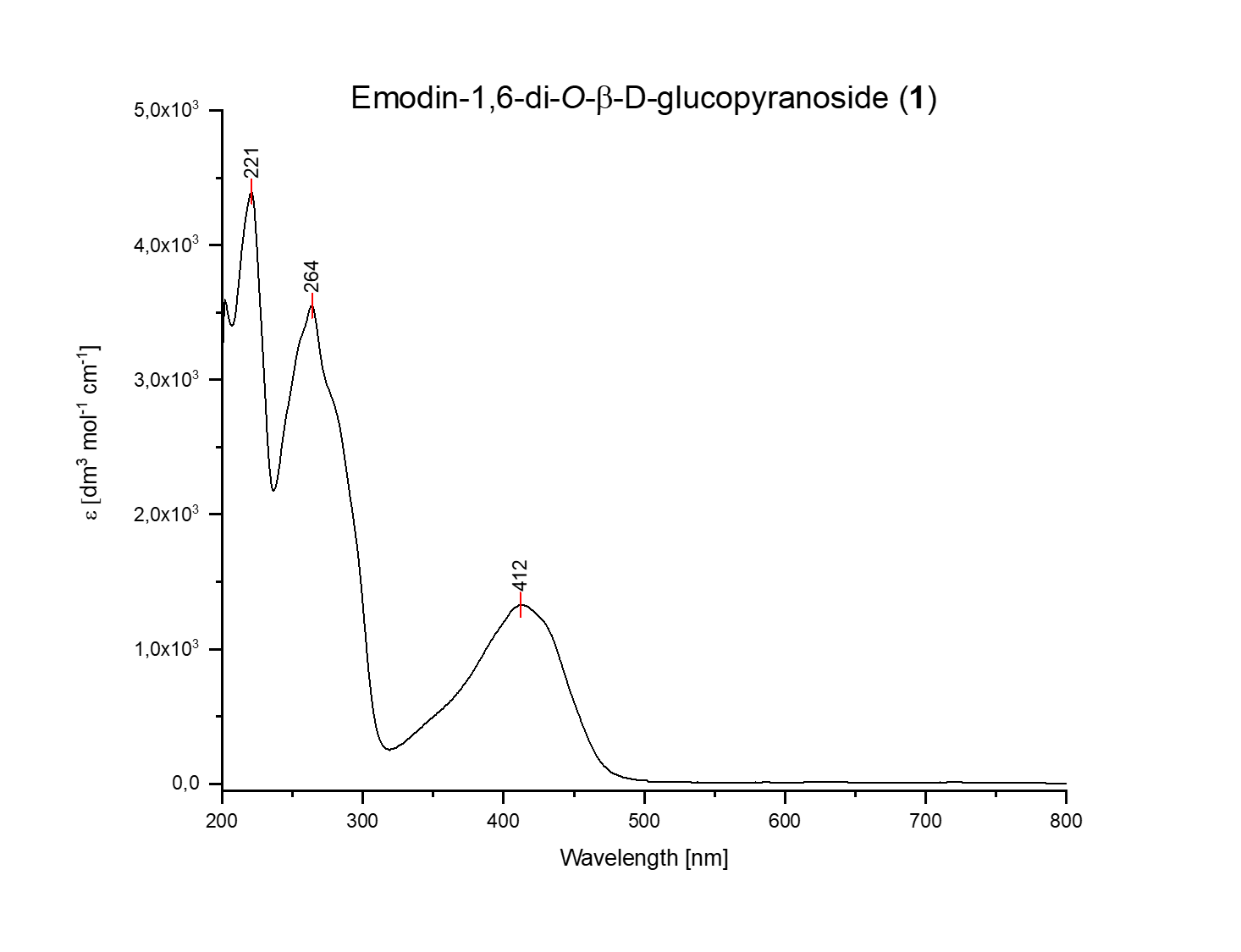


**Figure S5.** UV/Vis-spectrum of emodin-1,6-di-O-β-D-glucopyranoside (**1**) in MeOH.


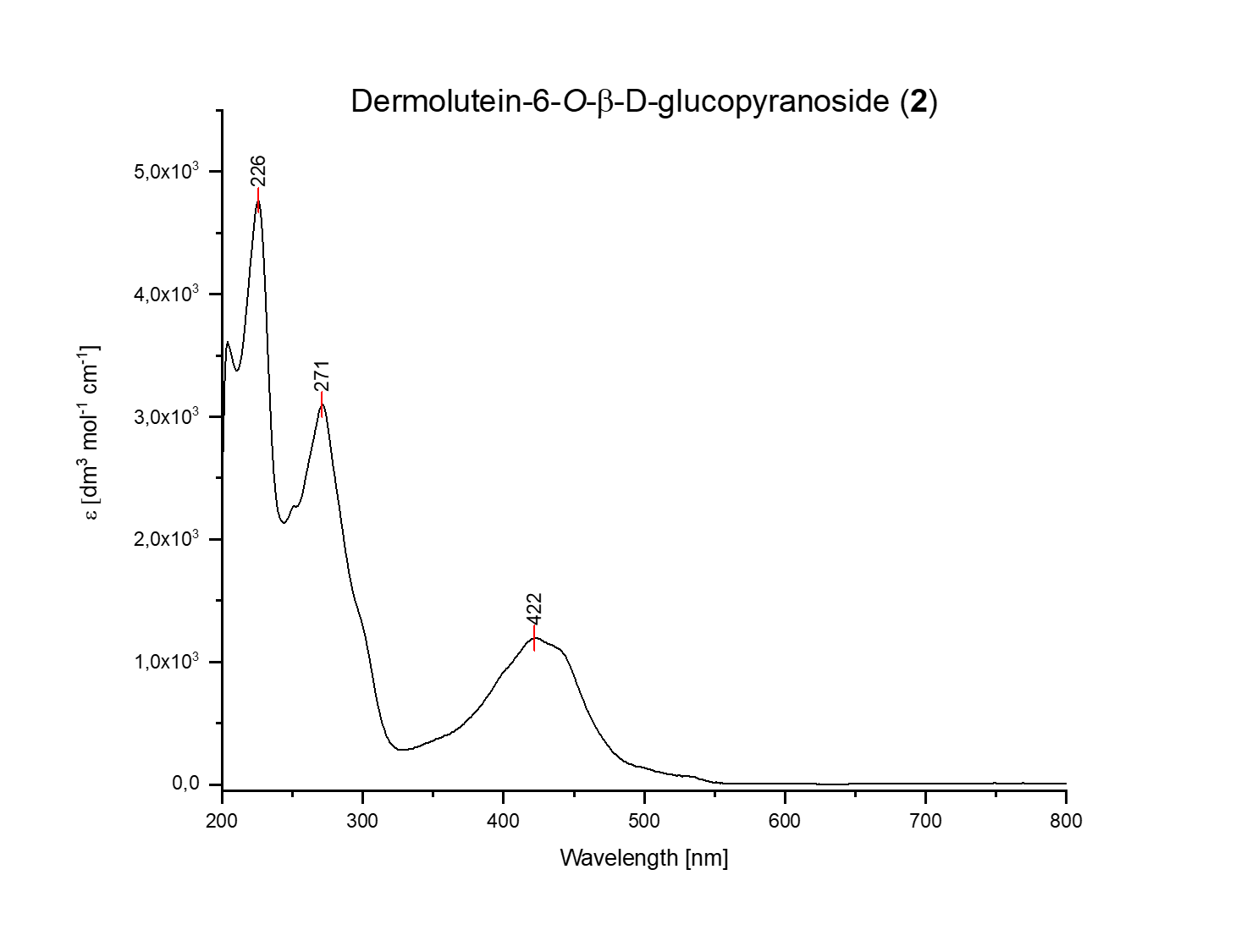


**Figure S6.** UV/Vis-spectrum of dermolutein-6-O-β -D-glucopyranoside (**2**) in MeOH.


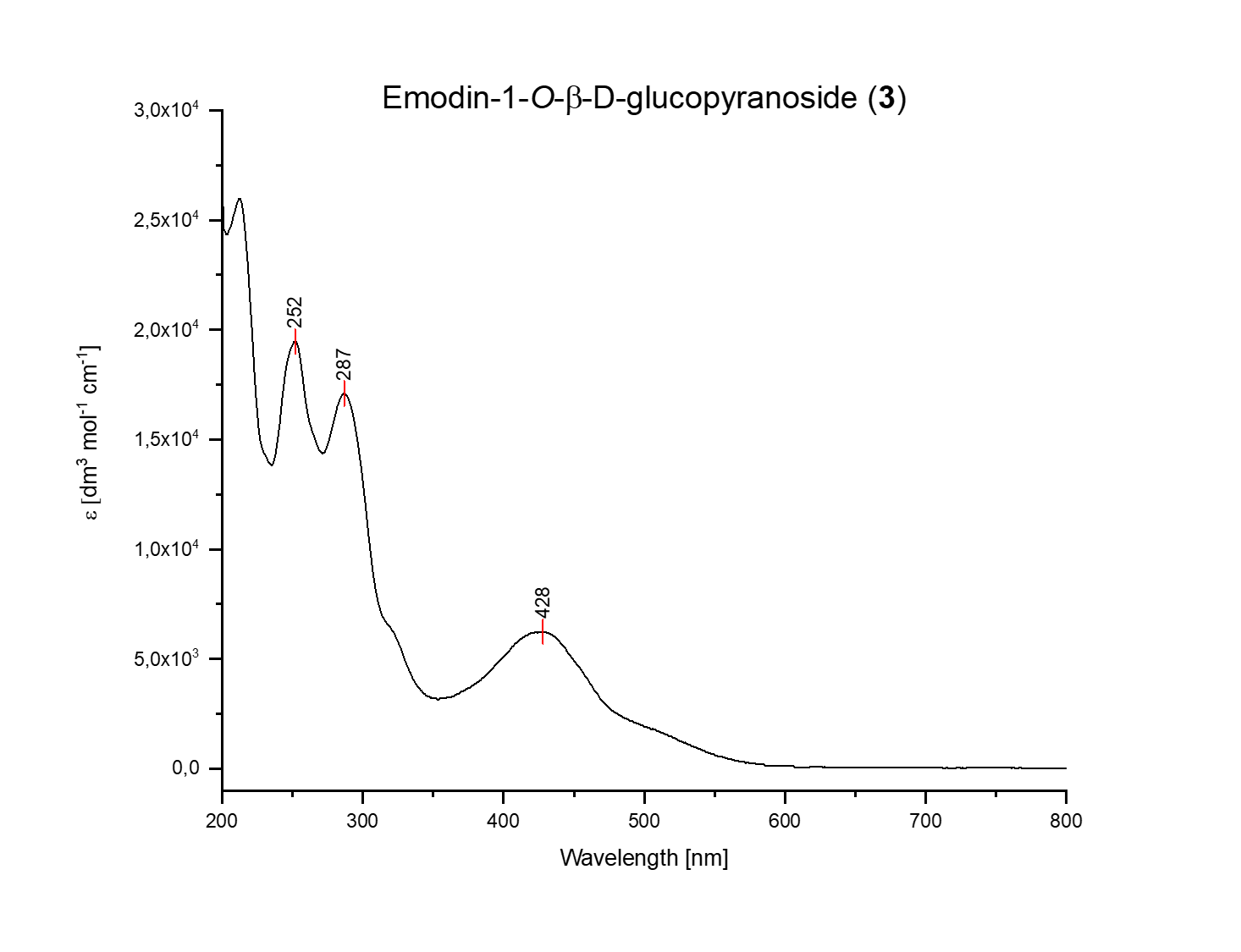


**Figure S7.** UV/Vis-spectrum of emodin-1-O-β-D-glucopyranoside (**3**) in MeOH.


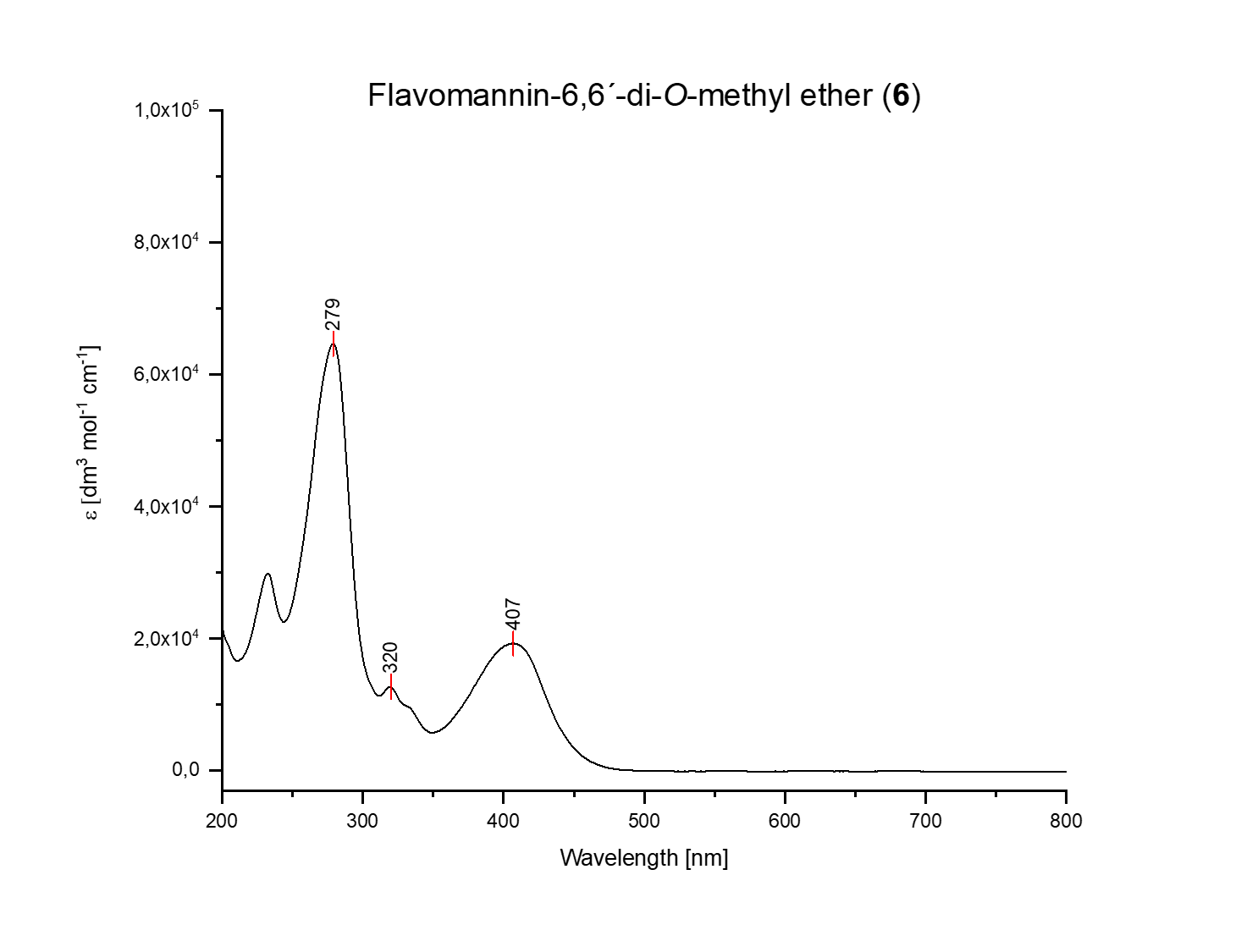


**Figure S8.** UV/Vis-spectrum of flavomannin-6,6´-di-O-methyl ether (**6**) in MeOH.


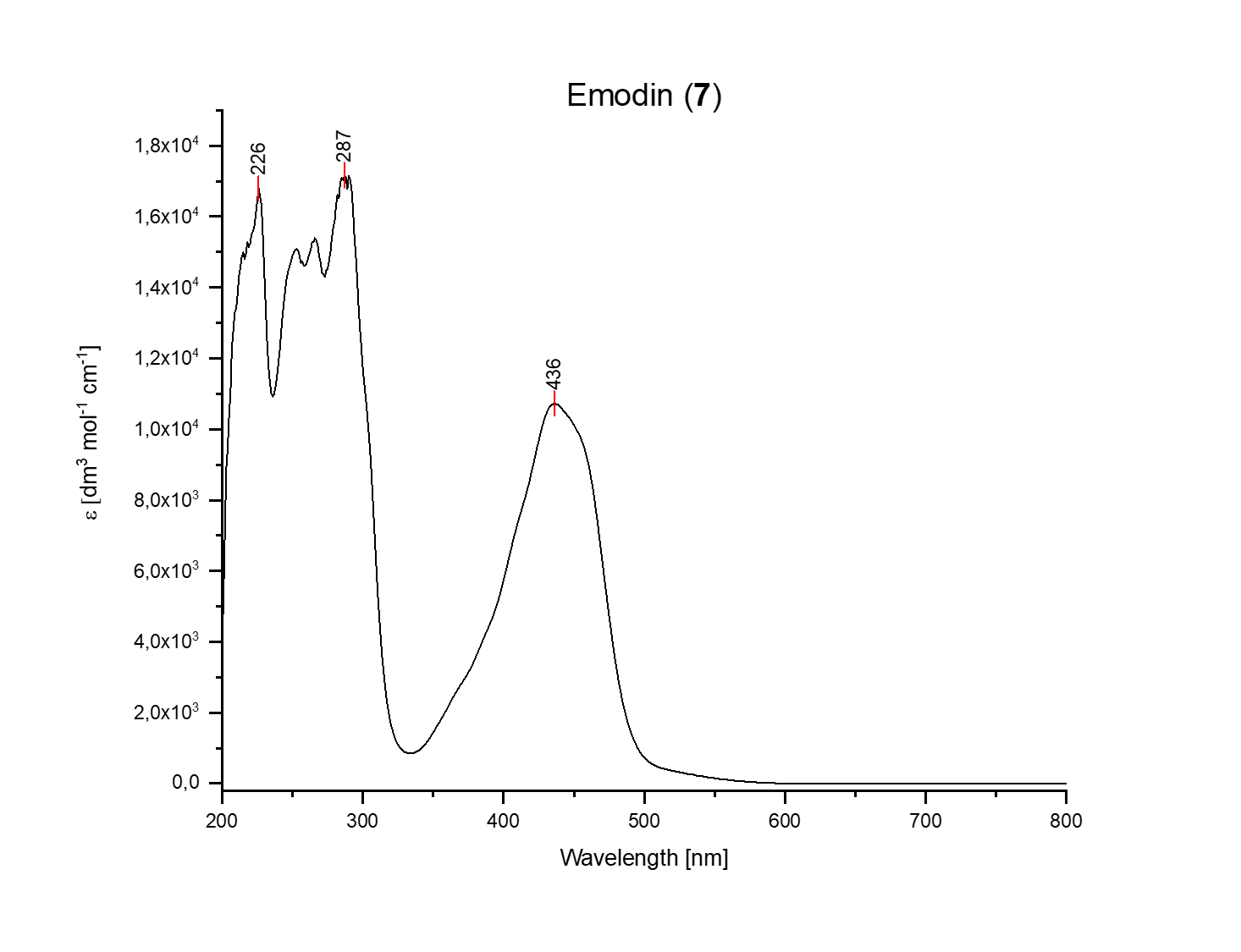


**Figure S9.** UV/Vis-spectrum of emodin (**7**) in MeOH.


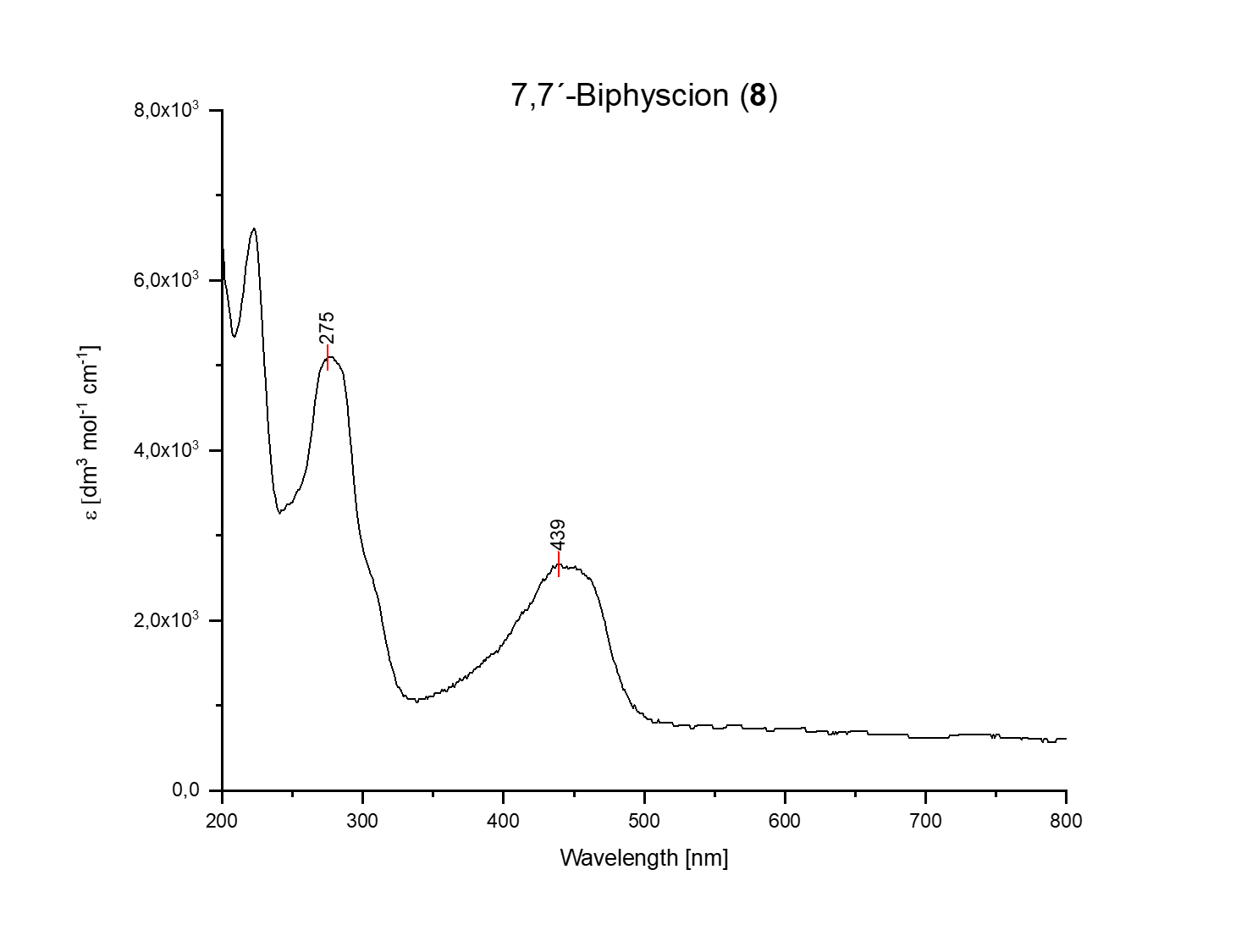


**Figure S10.** UV/Vis-spectrum of 7,7´-biphyscion (**8**) in MeOH.

### Spectral data: NMR


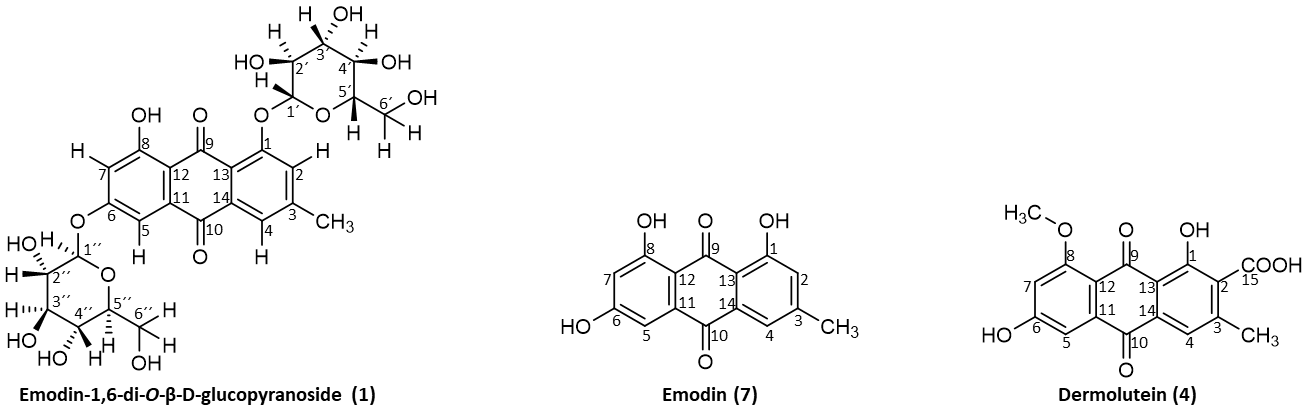


**Figure S11.** Numbering used for the isolated AQs (adopted from [8])

#### Compounds **1-3** NMR spectral data (tabular form)

**Table S5.** ^13^C NMR (150 MHz) and ^1^H NMR (600 MHz) chemical shifts for compounds **1** (D_2_O), **2** (D_2_O), and **3** (CD_3_OD). ^13^C values for **1** and **2** were mainly based on HSQC and HMBC experiments. Abbreviations: n.o. … not observed, - … not present.

|  | Compound **1** | | Compound **2** | | Compound **3** | |
| --- | --- | --- | --- | --- | --- | --- |
| C | δ C (ppm) | δ H (ppm) | δ C (ppm) | δ H (ppm) | δ C (ppm) | δ H (ppm) |
| 1 | n.o. | - | n.o. | 8.53 *s* | 158.8 | - |
| 2 | 124.2 | 7.19 *s* | n.o. | n.o. | 124.6 | 7.63 *s* |
| 3 | 148.5 C_ar_ \| 21.5 CH_3_ | 2.31 *s* | 141.9 C_ar_ \| 19.5 CH_3_ | 2.50 *s* | 147.0 C_ar_ \| 20.6 CH_3_ | 2.50 *s* |
| 4 | 123.2 | 7.13 *s* | 121.6 | 7.57 s | 122.5 | 7.82 *s* |
| 5 | 107.7 | 6.84 *d* | 107.4 | 7.38 *d* | 107.8 | 7.14 *s* |
| 6 | 162.1 | - | 162.5 | - | n.o. | 8.51 *s* |
| 7 | 110.0 | 6.62 *d* | 106.5 | 7.06 *d* | 108.0 | 6.55 *s* |
| 8 | n.o. | n.o. | 56.5 OCH_3_ | 4.01 *s* | n.o. | n.o. |
| 9 | n.o. | - | n.o. | - | n.o. | - |
| 10 | 181.8 | - | 183.6 | - | 182.4 | - |
| 11 | n.o. | - | n.o. | - | n.o. | - |
| 12 | 111.5 | - | 115.2 | - | 110.2 | - |
| 13 | 118.1 | - | 114.7 | - | 119.6 | - |
| 14 | n.o. | - | 136.7 | - | n.o. | - |
| 15 | - | - | n.o. | n.o. | - | - |
| 1´ | 101.3 | 4.99 *d* | 99.7 | 5.34 *d* | 102.7 | 5.02 *d* |
| 2´ | 73.0 | 3.82 – 3.62 *m* | 73.0 | 3.72 *dd* | 73.5 | 3.70 – 3.64 *dd* |
| 3´ | 76.2 |  | 75.6 | 3.78 *dd* | 75.9 | 3.58 – 3.52 *m* |
| 4´ | 69.4 |  | 69.8 | 3.62 *dd* | 70.0 | 3.46 – 3.42 *dd* |
| 5´ | 75.9 |  | 76.5 | 3.91 – 3.84 *m* | 77.2 | 3.58 – 3.52 *m* |
| 6´ | 60.7 | a: 4.12 – 4.04 *m*  b: 3.97 – 3.88 *m* | 61.1 | a: 4.09 *dd*  b: 3.91 – 3.84 *m* | 61.3 | a: 3.97 *dd*  b: 3.74 *dd* |
| 1´´ | 99.4 | 5.14 *d* | - | - | - | - |
| 2´´ | 76.5 | 3.82 – 3.62 *m* | - | - | - | - |
| 3´´ | 76.2 |  | - | - | - | - |
| 4´´ | 69.4 |  | - | - | - | - |
| 5´´ | 75.9 |  | - | - | - | - |
| 6´´ | 60.7 | a: 4.12 – 4.04 *m*  b: 3.97 – 3.88 *m* | - | - | - | - |

#### Emodin-1,6-di-*O*-β-D-glucopyranoside (**1**)


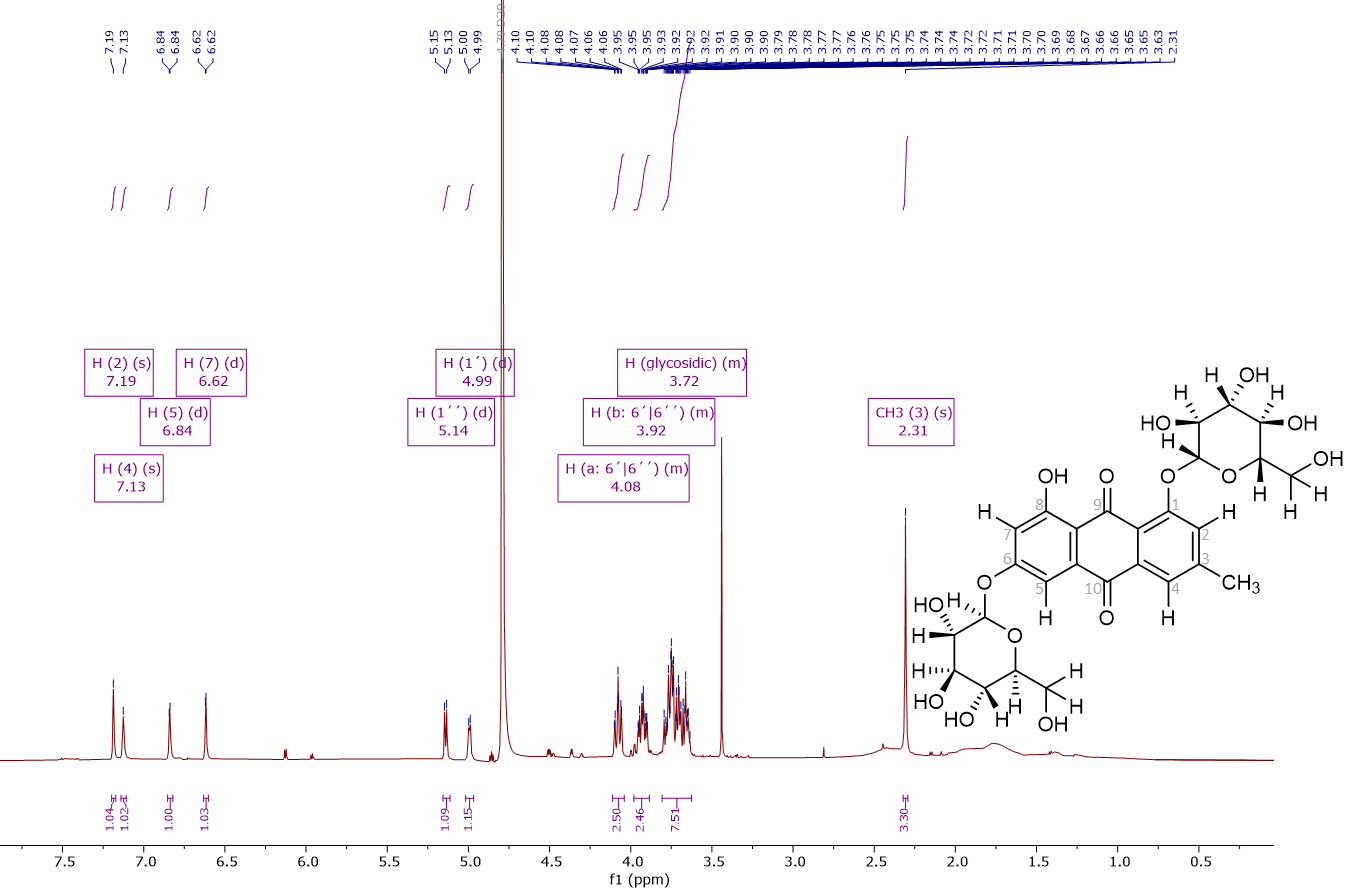


**Figure S12.** ^1^H-NMR (600.19 MHz, D_2_O, 25 °C): Emodin-1,6-di-O-β-D-glucopyranoside (**1**)


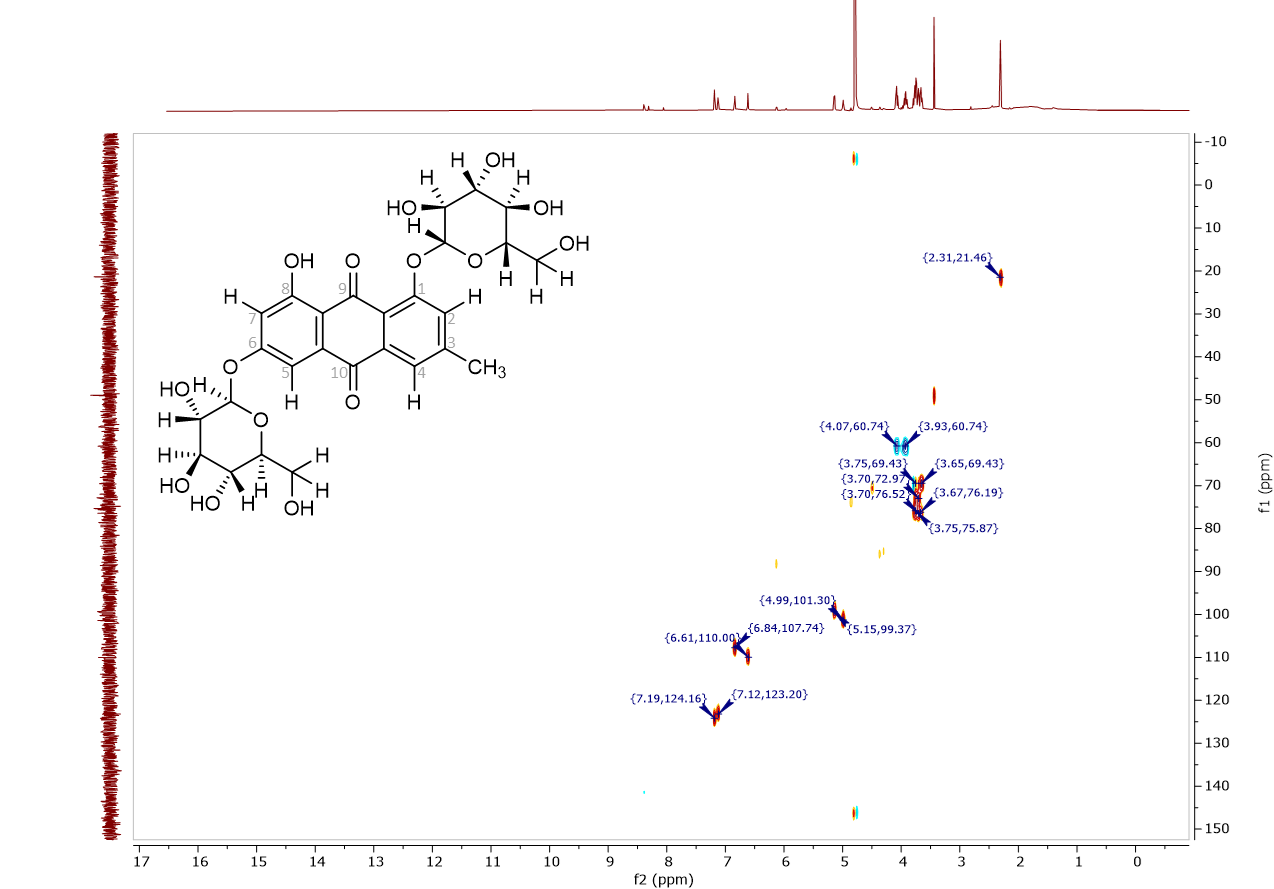


**Figure S13.** HSQC (600/150 MHz, D_2_O, 25 °C): Emodin-1,6-di-O-β-D-glucopyranoside (**1**)


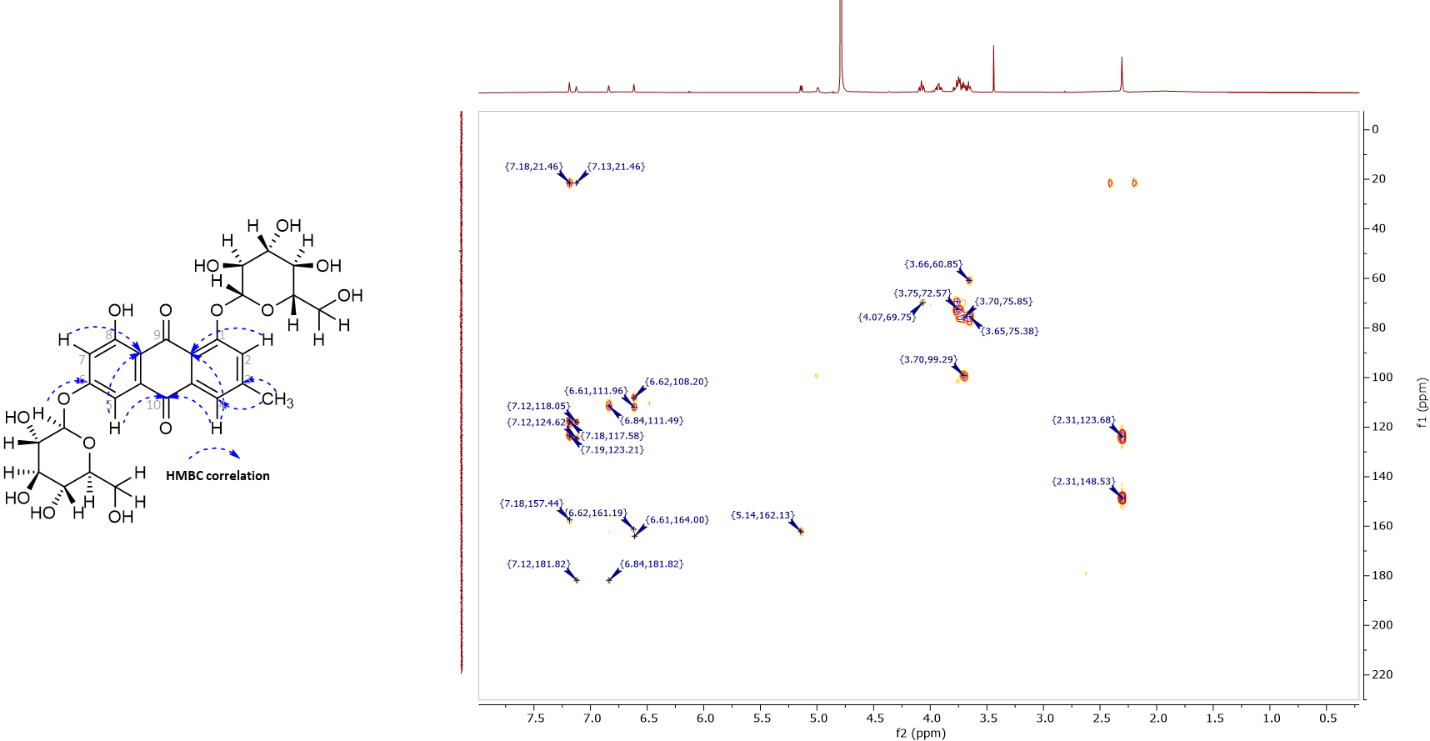


**Figure S14.** HMBC (600/150 MHz, D_2_O, 25 °C): Emodin-1,6-di-O-β-D-glucopyranoside (**1**)


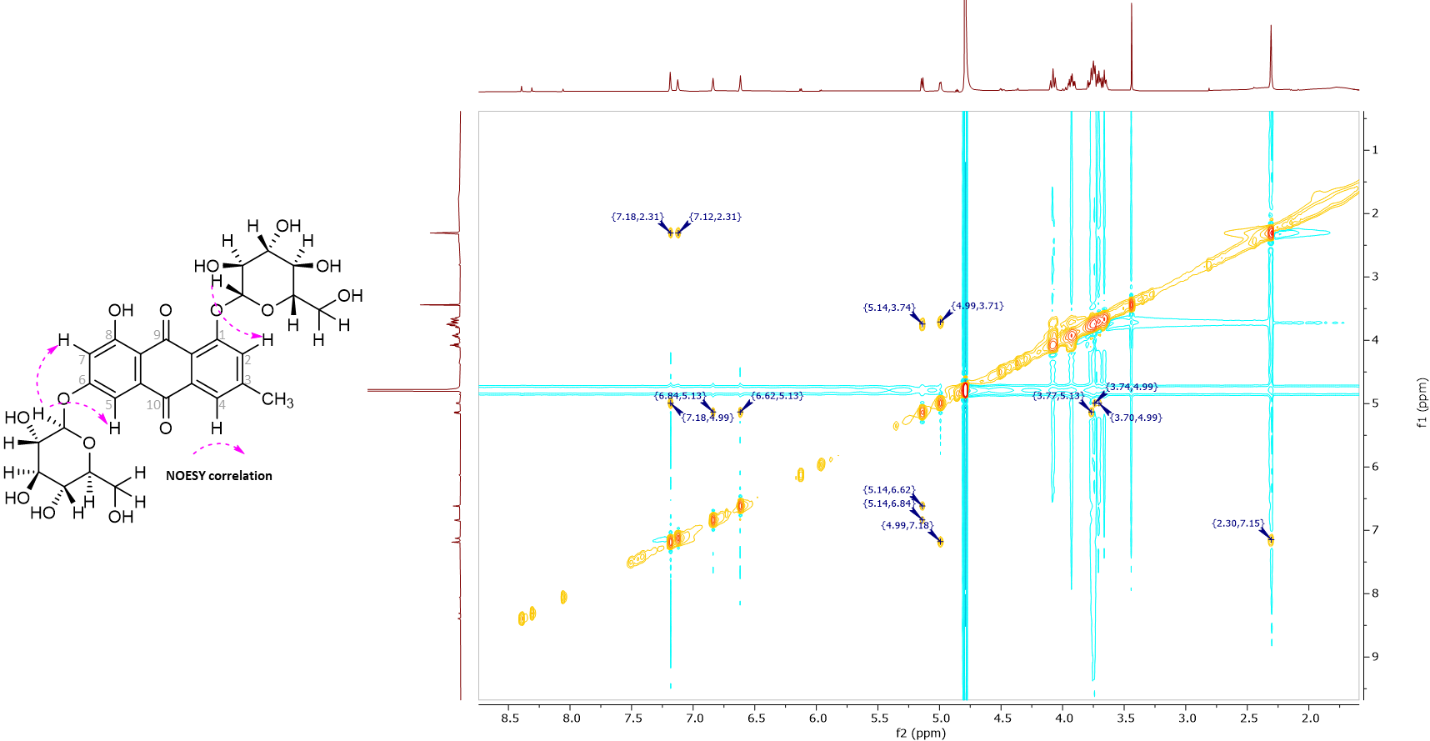


**Figure S15.** NOESY (600.19 MHz, D_2_O, 25 °C): Emodin-1,6-di-O-β-D-glucopyranoside (**1**)

#### Dermolutein-6-*O*-β-D-glucopyranoside (**2**)


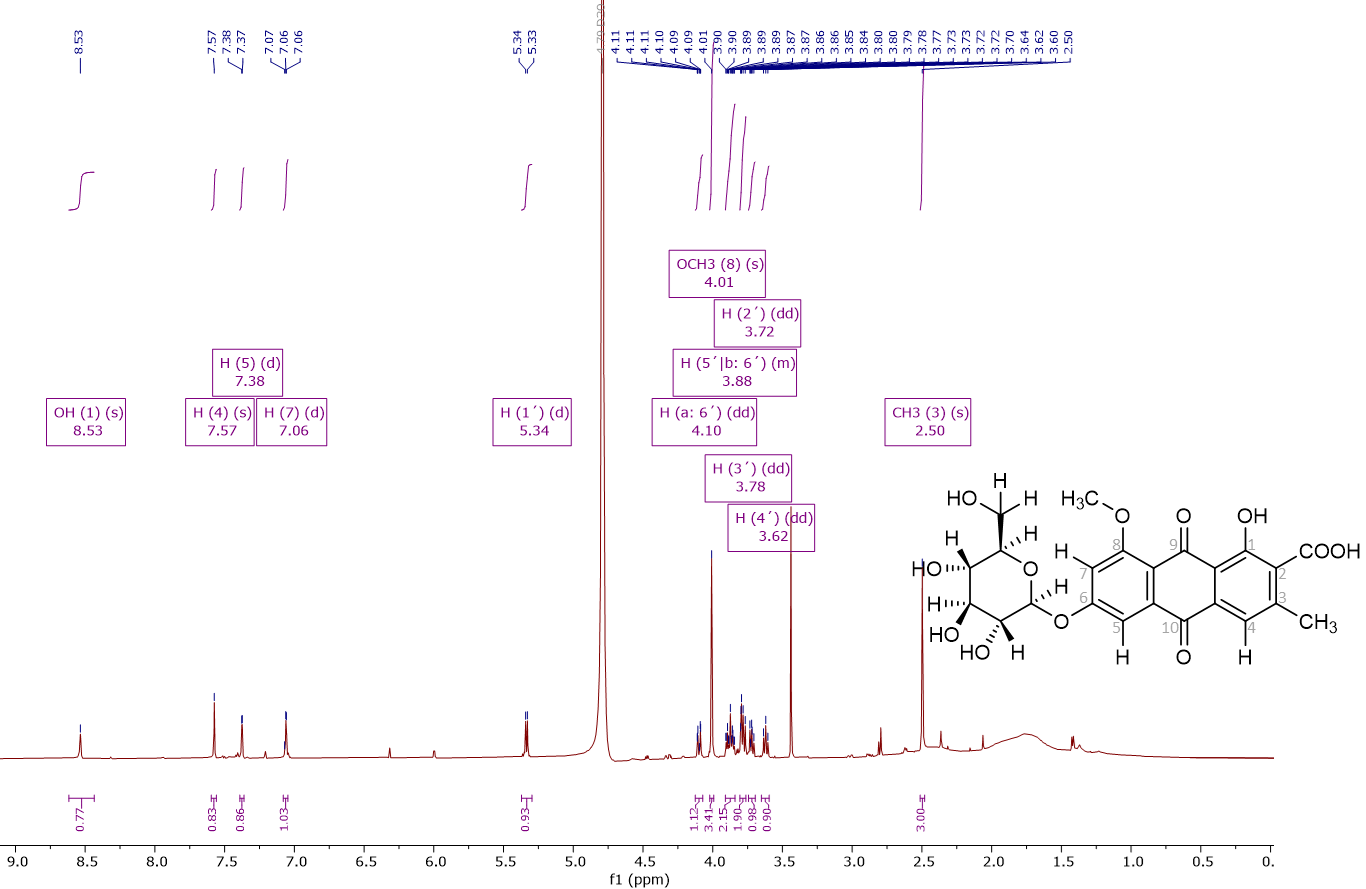


**Figure S16.** ^1^H-NMR (600.19 MHz, D_2_O, 25 °C): Dermolutein-6-O-β-D-glucopyranoside (**2**)


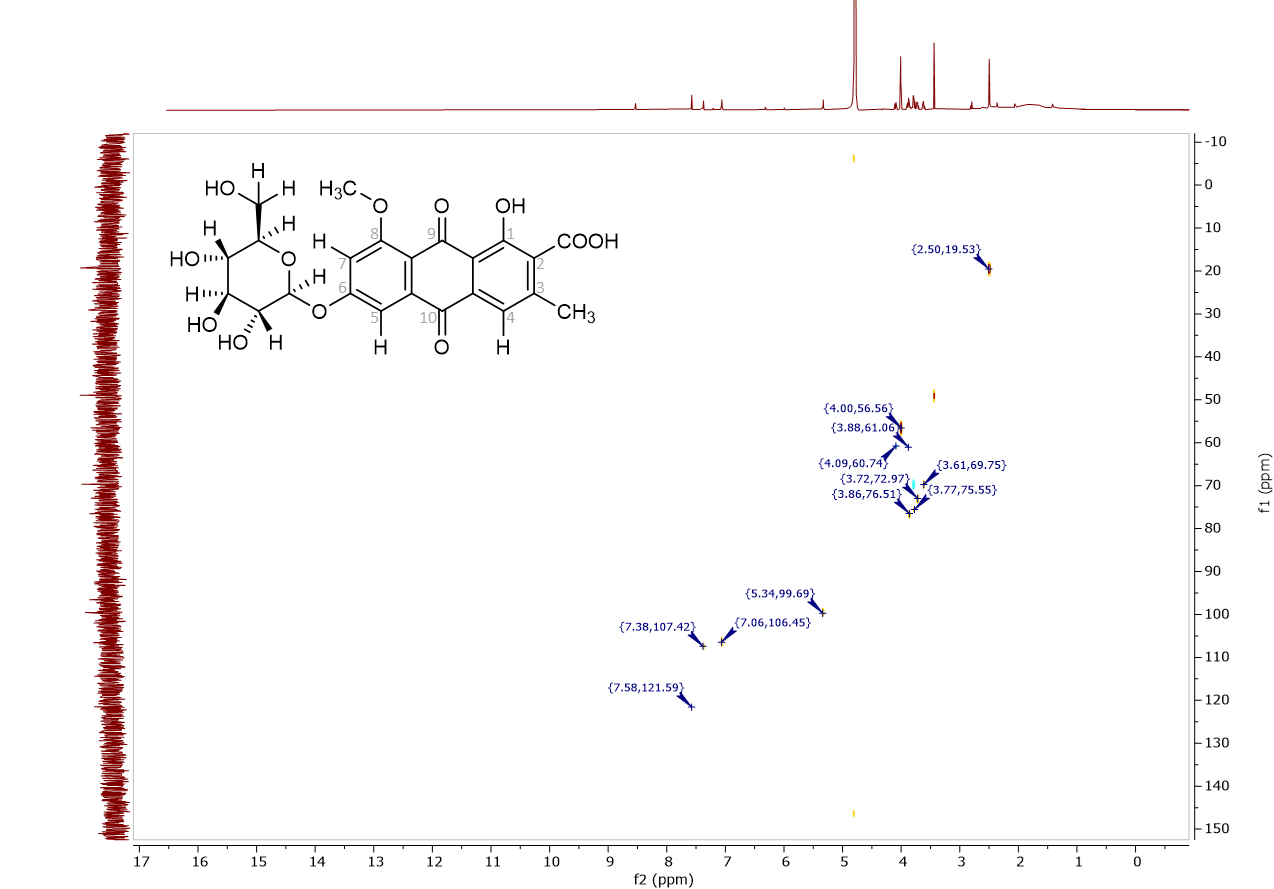


**Figure S17.** HSQC (600/150 MHz, D_2_O, 25 °C): Dermolutein-6-O-β-D-glucopyranoside (**2**)


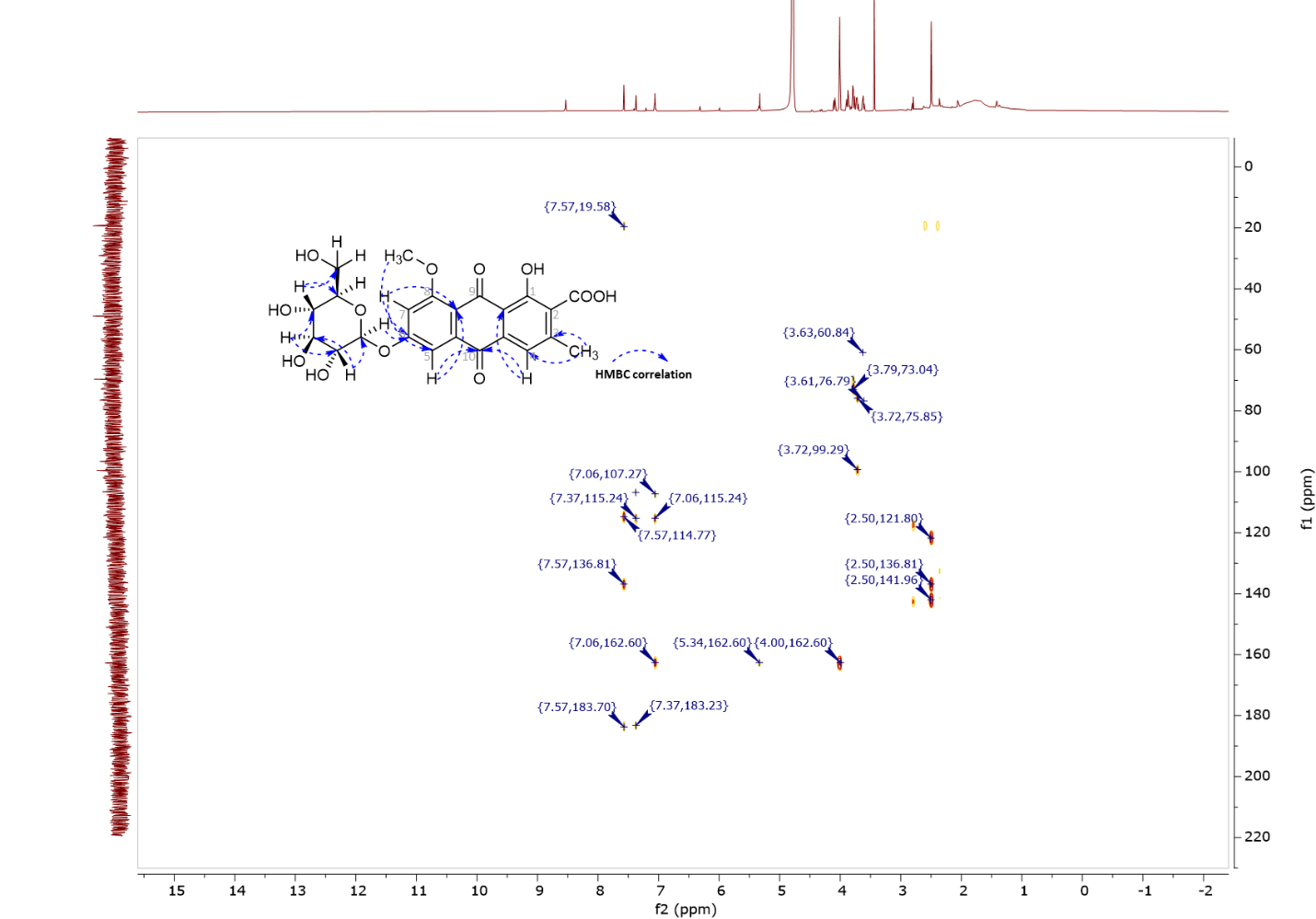


**Figure S18.** HMBC (600/150 MHz, D_2_O, 25 °C): Dermolutein-6-O-β-D-glucopyranoside (**2**)

#### Emodin-1-*O*-β-D-glucopyranoside (**3**)


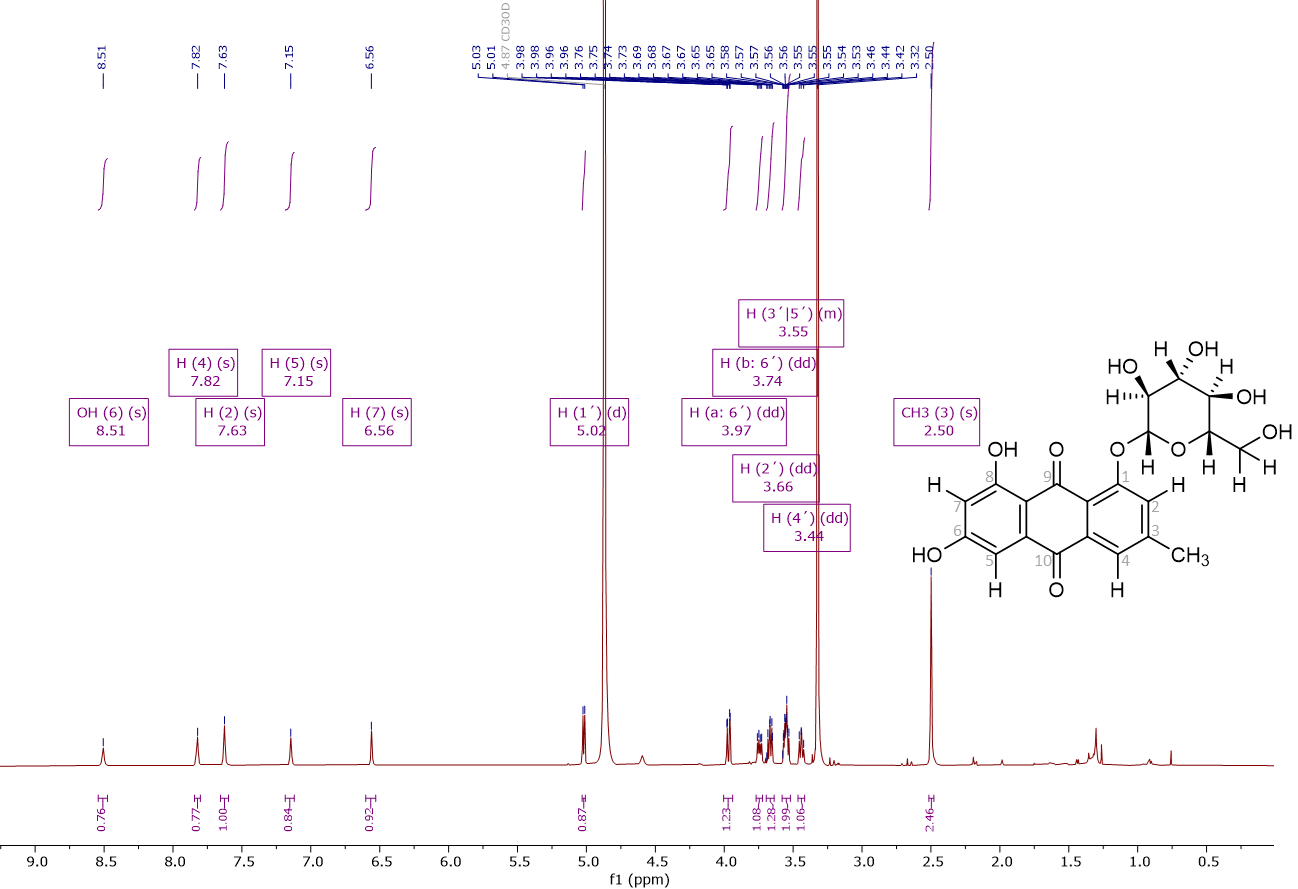


**Figure S19.** ^1^H-NMR (600.19 MHz, CD_3_OD, 25 °C): Emodin-1-O-β-D-glucopyranoside (**3**)


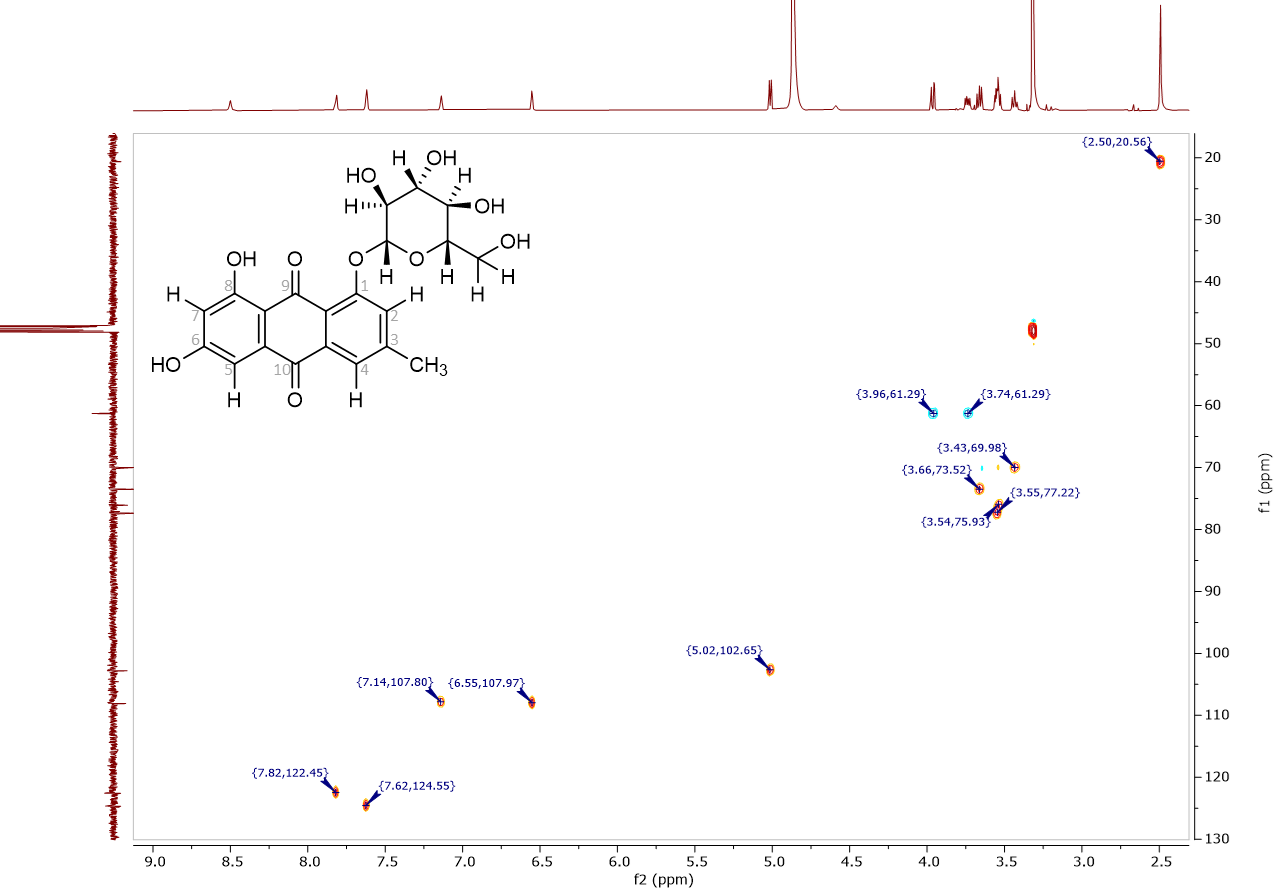


**Figure S20.** HSQC (600/150 MHz, CD_3_OD, 25 °C): Emodin-1-O-β-D-glucopyranoside (**3**)


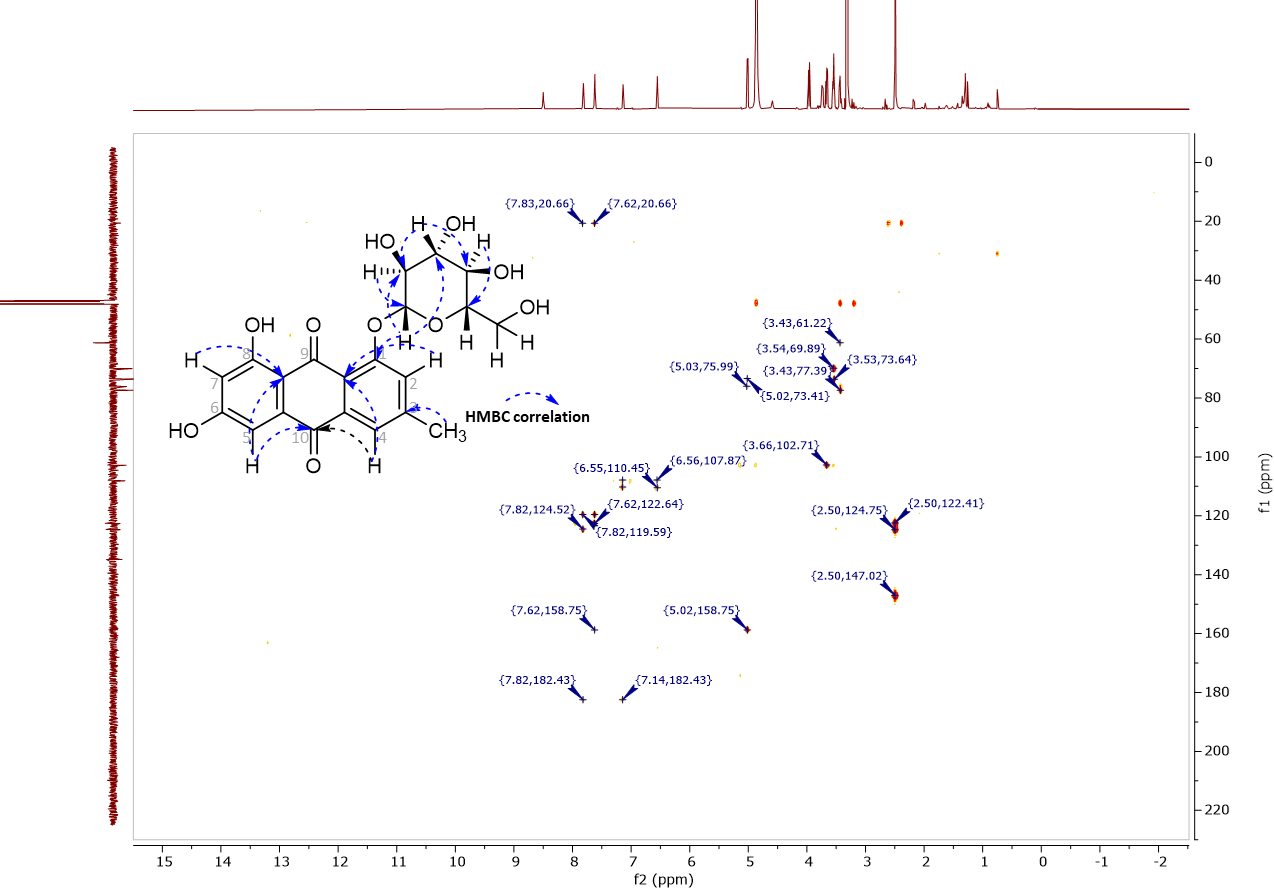


**Figure S21.** HMBC (600/150 MHz, CD_3_OD, 25 °C): Emodin-1-O-β-D-glucopyranoside (**3**)

## GC-MS analysis of the *C. rubrophyllus* methanol extract


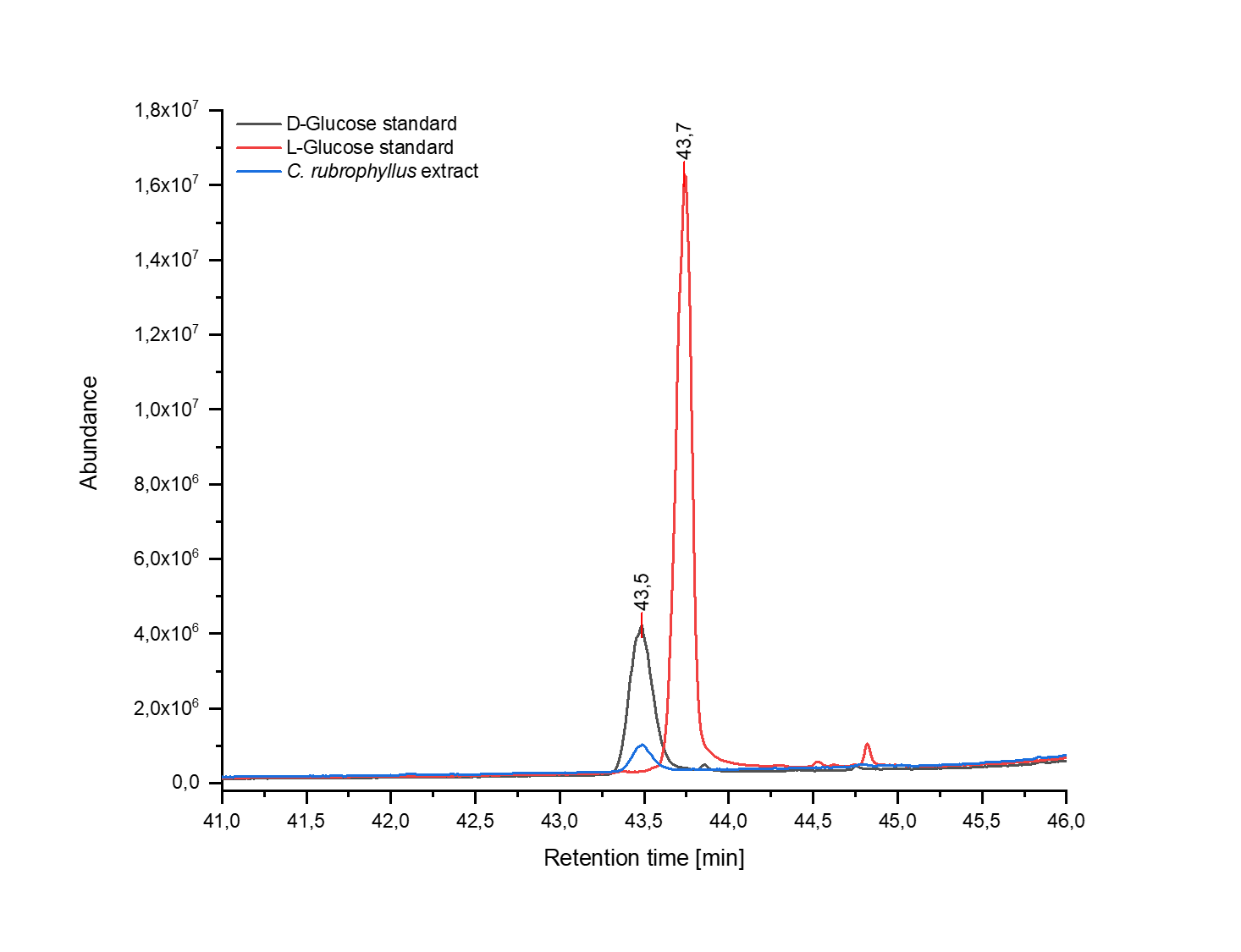


**Figure S22.** Results of the GC-MS analysis of the C. rubrophyllus methanol extract for the determination of the sugar moieties present in compounds **1-3**. The chromatogram depicts the thiazolidine derivatives of the C. rubrophyllus methanol extract´s hydrolysate (blue line), L-glucose (red line), and D-glucose (black line) standards after derivatization with BSTFA. The peaks representing the thiazolidine derivates of D-glucose and L-glucose showed retention times of 43.5 min and 43.7 min, respectively.

# Biological evaluation – (Photo)cytotoxicity assay

## EC_50_-values

The results of the (photo)cytotoxicity experiments are listed in the table below (Table S6). EC_50_ values including their 95% confidence intervals were calculated with GraphPad Prism 5 employing the “log(inhibitor) vs. normalized response”-equation (= relative Hill-Slope equation) with variable slope. Experiments were performed in at least three biological replicates (i.e. 9 technical replicates).

**Table S6.** The results of the (photo)cytotoxicity assay of emodin-1-O-β-D-glucopyranoside (**3**, EmodinGLC), flavomannin-6,6´-dimethyl ether (**6**, FDM), emodin (**7**), and 7,7´-biphyscion (**8**, 7,7´-BP). Berberine was tested as positive control at the following concentrations: 0.01, 0.13, 0.63, 1.25, 2.50, 6.25, and 12.50 µM. The compounds’ dark cytotoxicity as well as the amplification of their cytotoxic behaviour against the three cancer cell lines A549, AGS, and T24 upon blue light irradiation (λ = 468 ± 27 nm, 9.3 J/cm²) was evaluated. EC_50_ values in combination with their 95% confidence intervals are given in µM. The ratio of cells killed in the dark versus cells killed under irradiation (i.e. photoindex) is depicted as well.

| EC_50_ [µM] | A549  (BL, 468 nm) | | A549  (D) | | P.I. | AGS  (BL, 468 nm) | | AGS  (D) | | P.I. | T24  (BL, 468 nm) | | T24 (D) | | P.I. |
| --- | --- | --- | --- | --- | --- | --- | --- | --- | --- | --- | --- | --- | --- | --- | --- |
| EmodinGLC (**3**) | >25 |  | >25 |  |  | >25 |  | >25 |  |  | >25 |  | >25 |  |  |
|  |  |  |  |  |  |  |  |  |  |  |  |  |  |  |  |
| FDM (**6**) | 1.30 | 0.2 | 1.41 | 0.3 | 1.08 | 0.98 | 0.1 | 1.08 | 0.2 | 1.10 | 1.33 | 0.1 | 1.66 | 0.1 | 1.24 |
|  |  | 0.2 |  | 0.2 |  |  | 0.1 |  | 0.2 |  |  | 0.1 |  | 0.1 |  |
| Emodin (**7**) | 3.1 | 0.2 | 16.1 | 2.2 | 5 | 1.68 | 0.1 | 24.9 | 5.2 | 15 | 1.89 | 0.2 | 23.6 | 1.3 | 13 |
|  |  | 0.2 |  | 1.9 |  |  | 0.1 |  | 4.3 |  |  | 0.1 |  | 1.2 |  |
| 7,7´-BP (**8**)* | 0.06 | 0.03 | >2.50 |  | >40 | 0.04 | 0.03 | >2.5 |  | >61 | 0.07 | 0.04 | >2.5 |  | >63 |
| Berberine | 0.96 | 0.2 | >12.5 |  | >13 | 2.60 | 0.86 | >12.5 |  | >4.8 | 2.64 | 0.63 | >12.5 |  | >4.7 |
|  |  | 0.2 |  |  |  |  | 0.65 |  |  |  |  | 0.51 |  |  |  |

* The value was adopted from [4].

## Micrographs


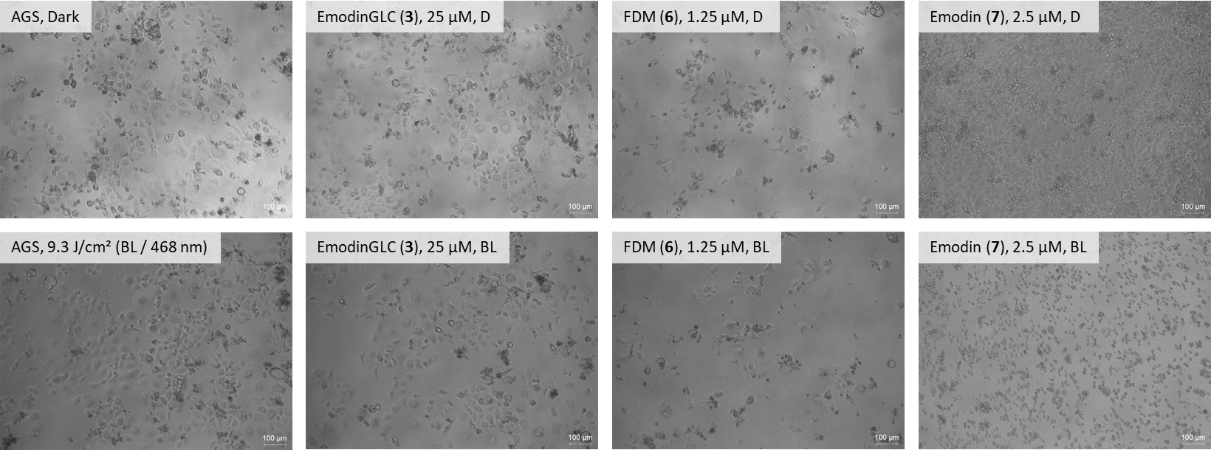


**Figure S23.** Micrographs of cells of the stomach cancer cell line (AGS / human Caucasian gastric adenocarcinoma, 10x objective) treated (24 h) with emodin-1-O-β-D-glucopyranoside (**3**, EmodinGLC, c = 25 µM), flavomannin-6,6´-dimethyl ether (**6**, FDM, c = 1.25 µM), and emodin (**7**, c = 2.5 µM). The upper line of pictures shows treated cells in the dark, the lower after irradiation with blue light (468 nm, 9.3 J/cm²).


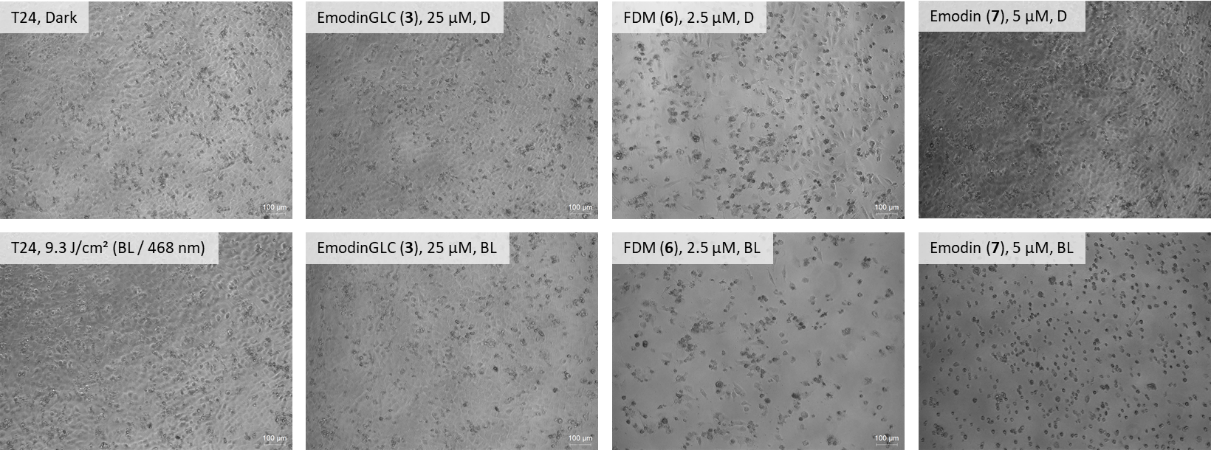


**Figure S24.** Micrographs of cells of the bladder cancer cell line (T24 / human bladder carcinoma, 10x objective) treated (24 h) with emodin-1-O-β-D-glucopyranoside (**3**, EmodinGLC, c = 25 µM), flavomannin-6,6´-dimethyl ether (6, FDM, c = 2.5 µM), and emodin (**7**, c = 5 µM). The upper line of pictures shows treated cells in the dark, the lower after irradiation with blue light (468 nm, 9.3 J/cm²).


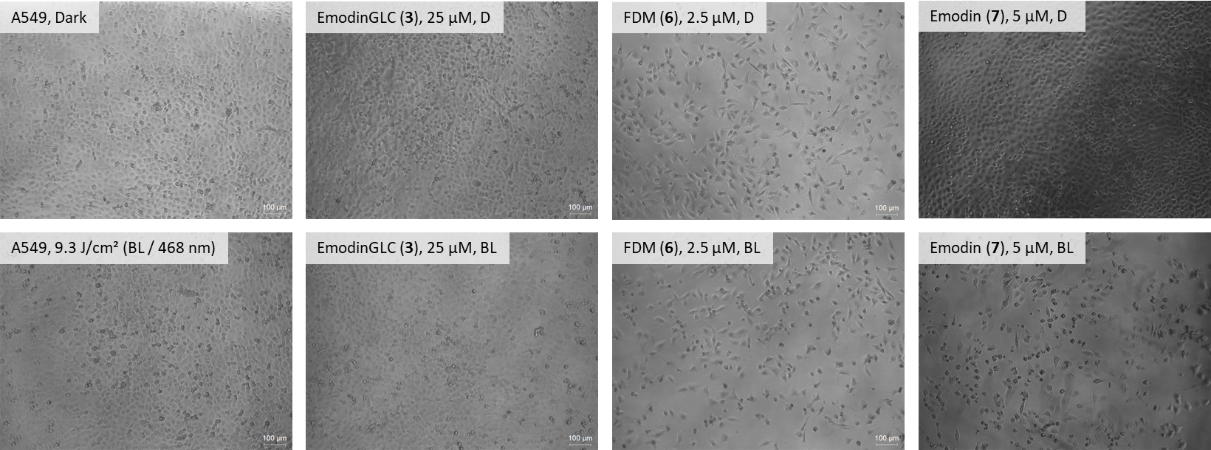


**Figure S25.** Micrographs of cells of the lung cancer cell line (A549 / human Caucasian lung carcinoma, 10x objective) treated (24 h) with emodin-1-O-β-D-glucopyranoside (**3**, EmodinGLC, c = 25 µM), flavomannin-6,6´-dimethyl ether (6, FDM, c = 2.5 µM), and emodin (**7**, c = 5 µM). The upper line of pictures shows treated cells in the dark, the lower after irradiation with blue light (468 nm, 9.3 J/cm²).

# On the isolation of compound **8**

A detailed description and discussion of the optimized isolation protocol of 7,7´-biphyscion (**8**, 7,7´-BP) is provided in the main manuscript. Figure S26 is a visual representation of the workflow yielding a 7,7´-biphyscion-enriched extract without its precursor flavomannin-6,6´-dimethyl ether (**6**, FDM). After the dehydratisation step, only small amounts of **6** remain. Oxidation with NaOH and H_2_O_2_ leads to the complete loss of **6** and the formation of high amounts of **8**, which can be isolated via further chromatographic separation steps (i.e., vacuum column chromatography).


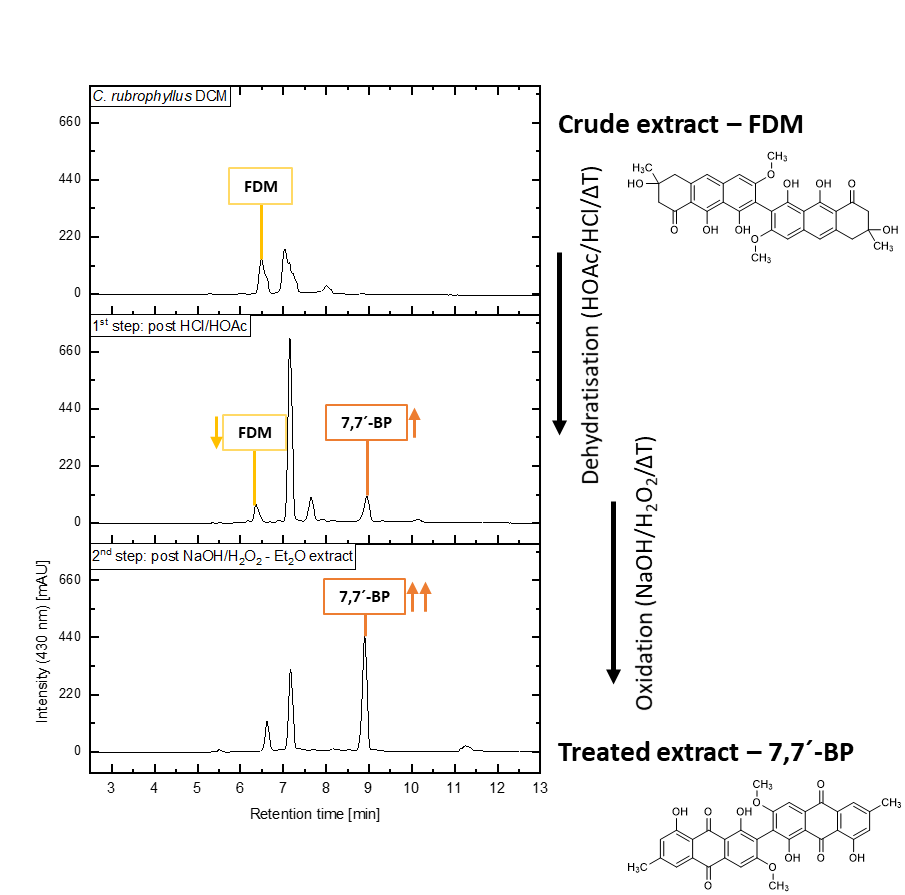


**Figure S26.** Results of the HPLC-DAD-MS analysis (λ = 430 nm) of the crude DCM extract and the extracts resulting from the treatment protocol (middle chromatogram: after the dehydratisation step, bottom chromatogram: after the oxidation step and extraction with diethyl ether). All samples were dissolved in DMSO prior to analysis (c_extract_ = 1 mg/mL, c_step 1&2_ = 2 mg/mL). Stationary Phase: Phenomenex Synergi MAX-RP, Mobile Phase: H_2_O/ACN + 0.1% FA, ratio of solvent B (% ACN + 0.1% FA) is displayed in Figure 1 (main manuscript). Peak annotation – middle chromatogram: RT = 6.5 min 🡪 flavomannin-6,6´-dimethyl ether (**6**), RT = 7.3 min 🡪 emodin (**7**), RT = 7.7 min 🡪 **?**, RT = 9.0 min 🡪 7,7´-biphyscion (**8**), bottom chromatogram: RT = 6.6 min 🡪 **?** (≠ **6**).

## Mycochemical analysis of *Cortinarius holoxanthus*

### Extract preparation

Dried *C. holoxanthus* fruiting bodies were ground with mortar and pestle to yield a fine powder. The powdered biomaterial was successively extracted with solvents of different polarity (petroleum ether: n = 2, V = 20 mL; dichloromethane: n = 3, V = 30 mL; methanol: n = 4, V = 50 mL) via ultrasonication (t = 5 min) followed by filtration. Solvents were removed by vacuum rotary evaporation at 40 °C. The extract yields are given in Table S7.

**Table S7.** Cortinarius holoxanthus **–** Extract yields.

| **Ultra-sonication** | **Mass biomaterial [mg]** | **Solvent** | **Yield [mg (%dw)]** |
| --- | --- | --- | --- |
| *Cortinarius holoxanthus* | 326.4 | Petroleum ether | 6.9 (2.11%) |
|  |  | Dichloromethane | 8.9 (2.73%) |
|  |  | Methanol | 115.7 (35.45%) |

### HPLC-DAD analysis

**Figure S27.** HPLC-DAD analysis of the petroleum ether (PE), dichloromethane (DCM), and methanol (MeOH) extract of C. holoxanthus. All extracts were dissolved in DMSO (c = 2 mg/mL) prior to analysis. Stationary Phase: Phenomenex Synergi MAX-RP, Mobile Phase: H_2_O (A)/ACN +0.1% FA (B), ratio of solvent B is displayed in the top chromatogram by the blue line. Chromatograms were recorded at λ = 430 nm.

### Secondary metabolite annotation


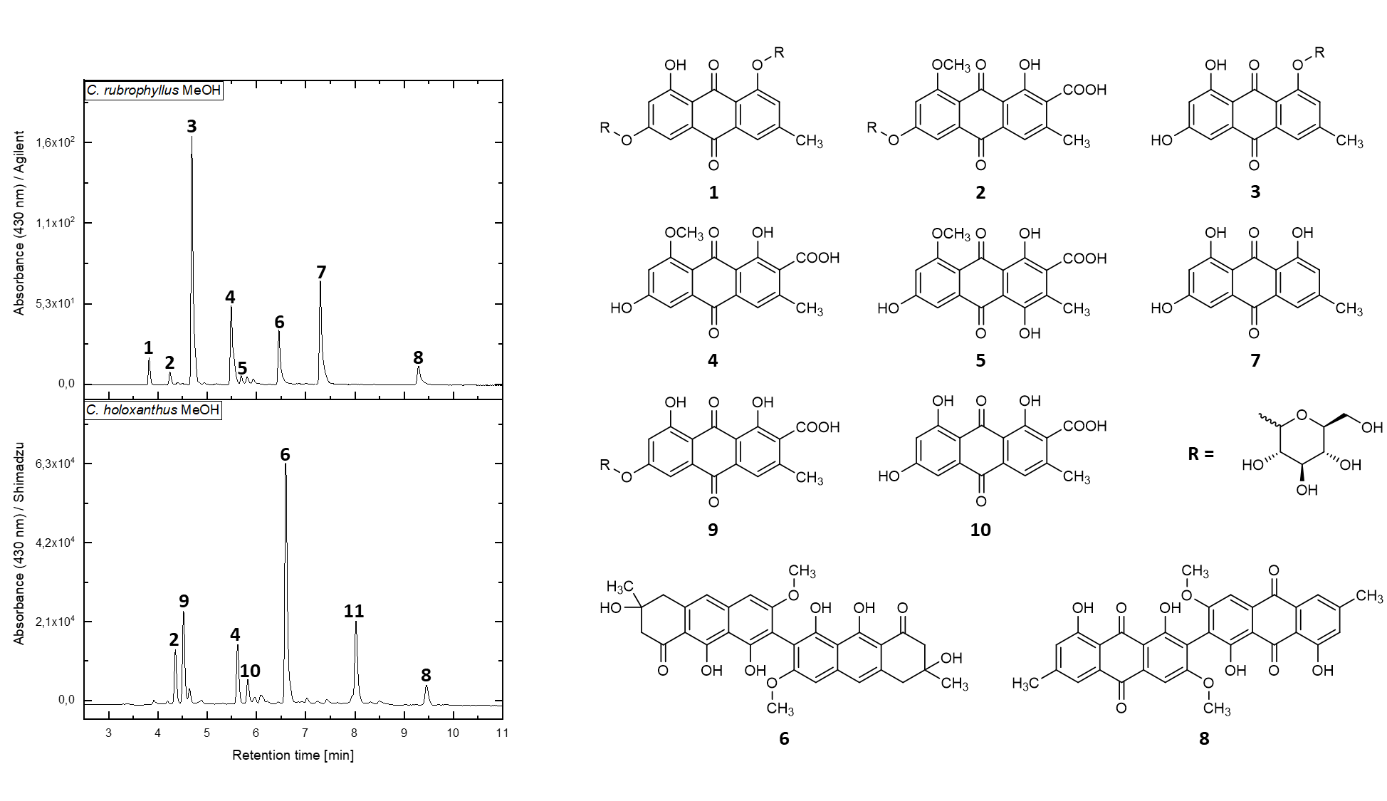


**Figure S28.** Chromatograms (λ = 430 nm) from the HPLC-DAD analysis of the methanolic extracts of C. rubrophyllus (top, instrument = Agilent Technologies 1100) and C. holoxanthus (bottom, instrument = Shimadzu LC-20AD XR). Both extracts were dissolved in DMSO (c = 2 mg/mL) prior to analysis. Stationary Phase: Phenomenex Synergi MAX-RP, Mobile Phase: H_2_O/ACN +0.1% FA. The gradient used for chromatographic separation (%ACN + 0.1% FA) is depicted in Figure S27. Numbered peaks were either annotated via spectral comparison (UV/Vis, mass spectrum) with authentic reference samples (in-house data) or isolated (compound **8**).

The HPLC-DAD-MS analysis of the *C. holoxanthus* methanol extract showed some similarity to the *C. rubrophyllus* extract in terms of their pigment profiles. Based on a comparison of retention times, UV/Vis-spectra, and mass spectra of both extracts, the peaks with retention times of 4.25 min, 5.49 min, 6.46 min, and 9.38 min in the *C. holoxanthus* extract (Figure S28) were annotated as compounds **2**, **4**, **6**, and **8**, respectively. The peaks **9**, **10**, and **11** were not further identified yet.

**Table S8.** Results of the metabolite annotation for the C. holoxanthus methanol extract. Mass peaks and UV/Vis spectral data were gathered from the HPLC-DAD-MS analysis.(n.i. … not identified)

| **Name / Suggested compound** | **Compound #** | **Retention time [min]** | **[M-H]^-^** | **UV/Vis: λ_max_ in H_2_O/ACN + 0.1% FA [nm]** | **In accordance with literature? (yes/no) [9]** |
| --- | --- | --- | --- | --- | --- |
| Dermolutein-6-*O*-β-D-glucopyranoside | **2** | 4.25 | 489.1 | 224, 270, 428 | yes |
| n.i. | **9** | 4.46 | 475.2 | 207, 265, 436 | - |
| Dermolutein | **4** | 5.49 | 326.9 | 214, 286, 437 | yes |
| n.i. | **10** | 5.82 | n.d. | 209, 287, 430 | - |
| Flavomannin-6,6´-dimethyl ether | **6** | 6.46 | 573.3 | 210, 278, 319, 404 | yes |
| n.i. | **11** | 8.01 | n.d. | 211, 274, 413 | - |
| 7,7´-Biphyscion | **8** | 9.38 | 565.2 | 211, 280, 438 | yes |

### *Cortinarius holoxanthus* – Isolation of 7,7´-biphyscion (**8**)

An aliquot of the *C. holoxanthus* dichloromethane extract (m = 8.4 mg) was dissolved in a mixture of acetic acid (V = 5 mL) and concentrated hydrochloric acid (V = 0.2 mL) and refluxed (T_water bath_ = 100 °C) for 1 hour. After cooling the reaction mixture to room temperature and adding 15 mL of water, the solution was extracted with dichloromethane (V = 20 mL, n = 2). The organic phases were combined, the solvents were removed by vacuum rotary evaporation at 40 °C, and the resulting extract was kept in a desiccator (C1, η = 7.7 mg, 91.7% w/w). C1 (m = 7.5 mg) was dissolved in a mixture of sodium hydroxide solution (c = 1 mol/L, V = 15 mL) and 30% hydrogen peroxide solution (V = 0.2 mL) and warmed 3 hours to 60 °C. Then, the reaction mixture was acidified with acetic acid, diluted with water (V $\sim$10 mL), and extracted with dichloromethane (V = 25 mL, n = 2). The combined organic extract was dried via vacuum rotary evaporation at 40 °C and kept in a desiccator (C1.1, η = 7.2 mg, 85.7% w/w).
An aliquot of the fraction C1.1 (m = 4.0 mg) was dissolved in 1 mL of a mixture of chloroform, acetone, and methanol (1:1:1) and loaded onto 4 TLC plates for the purpose of preparative TLC (pre-coated TLC sheets, 10 x 20 cm, silica gel 60 F254 0.20 mm layer). Toluene/ethyl acetate/formic acid/acetic acid (60:30:5:5) was chosen as mobile phase. Every plate was developed over a separation distance of approximately 8 cm. In total, three zones were scraped out (Z1/R_f_ = 0.55, Z2/R_f_ = 0.68, Z3/R_f_ = 0.90), pooled and extracted (Z1 & Z2: acetone, Z3: chloroform). The extracts were filtrated through cotton wool and dried under an air stream. The purification of C1.1. by preparative TLC yielded 2.9 mg of Z1, 1.0 mg of Z2, and 0.6 mg of Z3. Z1 and Z2 showed signs of SiO_2_-impurities, being responsible for the comparatively high yields. However, they were not further purified, because Z3 (7,7´-biphyscion, **8**) was the target of this isolation procedure.

### HPLC-DAD-MS analysis


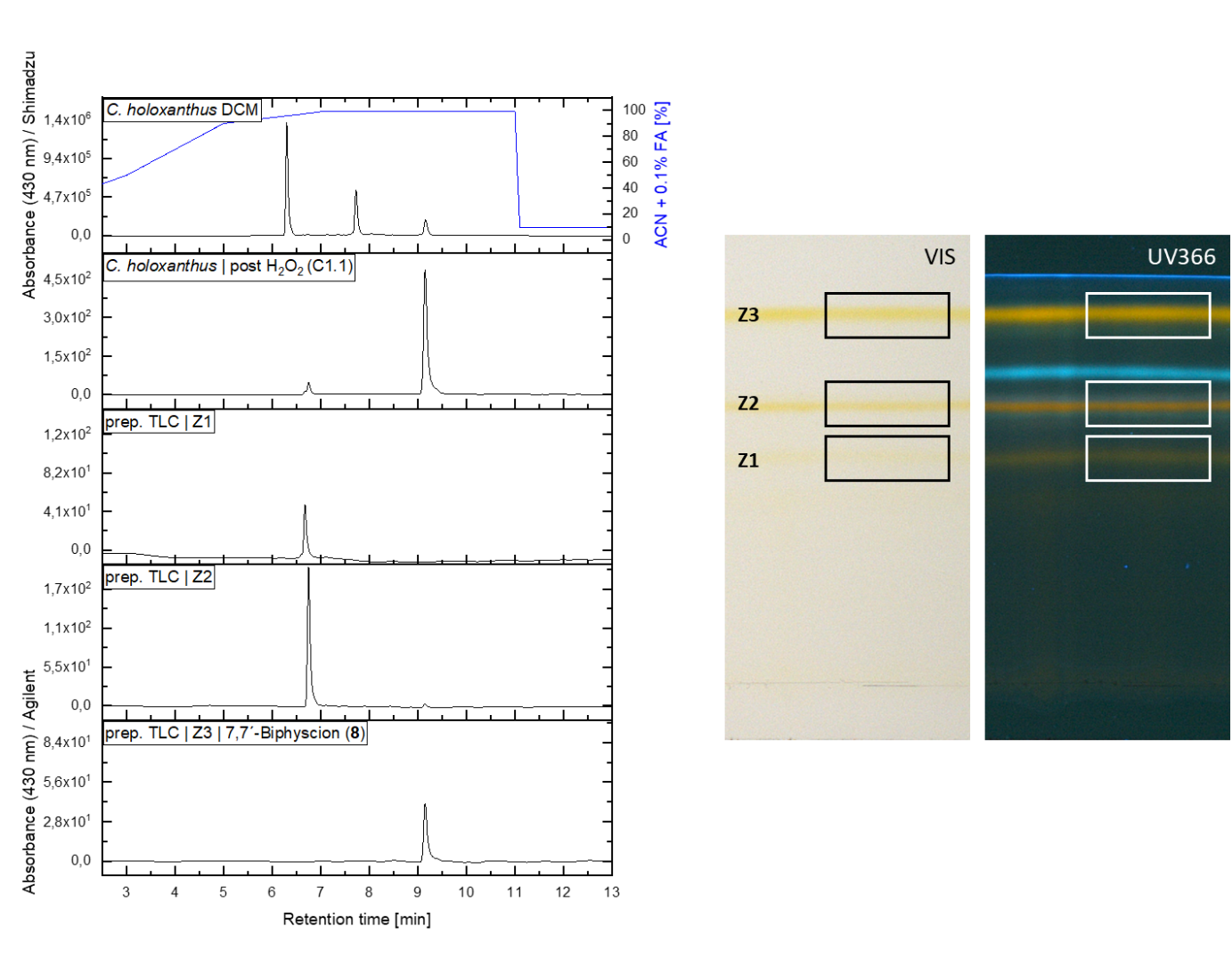


**Figure S29.** Results of the HPLC-DAD-MS analysis (left) conducted in the framework of isolating **8** from the C. holoxanthus dichloromethane extract as well as pictures of a developed TLC plate (right, VIS and UV 366 nm) used in the preparative separation process (stationary phase: SiO_2_, mobile phase: toluene/ethyl acetate/formic acid/acetic acid = 60:30:5:5) of fraction C1.1. Chromatograms of the crude extract, the fraction C1.1 (post NaOH/H_2_O_2_ treatment), as well as the fractions Z1-3, which were obtained by scraping out respective zones on TLC plates (Z1: R_f_ = 0.55, Z2: R_f_ = 0.68, Z3: R_f_ = 0.90), were recorded at λ = 430 nm. Parameters of the HPLC experiment were as described in Figure S27.

#### Identification of fractions Z1-Z3

Dereplication based on spectral comparison (UV/Vis-spectra and MS-spectra) failed to give definitive structural suggestions for fractions Z1-2. However, it can be anticipated that Z1 and Z2 are oxidized flavomannin-type compounds (similar to 7,7´-biphyscion), due to their yellow coloration (see UV/Vis-spectra of Z1-3: Figure S30) and their potential shared precursor compound (flavomannin-6,6´-dimethylether). Fraction Z3 was identified as 7,7´-biphyscion (**8**) by comparing its UV/Vis-spectrum and retention time with an authentic reference compound. In addition, its identity was further verified via ^1^H-NMR spectroscopy. Spectral data acquired by the HPLC-DAD-MS analysis of the fractions Z1-3 is depicted in Table S9.

**Table S9.** Retention times and spectral data (MS, UV/Vis, and NMR) of the fractions Z1-3. (n.d. … not determined)

|  | **Suggested compound** | **Retention time [min]** | **m/z** | **UV/Vis: λ_max_ in H_2_O/ACN + 0.1% FA [nm]** | **^1^H-NMR (400 MHz)** |
| --- | --- | --- | --- | --- | --- |
| Z1 | Ox. flavomannin-type compound | 6.68 | MS1: 636.3284, 732.5572, 847.5070, 974.0383  MS2: 387.88, 546.97, 590.99 | 211, 268, 437 | n.d. |
| Z2 | Ox. flavomannin-type compound | 6.75 | MS1: 326.7087, 376.9838, 657.4854  MS2: 282.94, 325.04 | 210, 268, 436 | n.d. |
| Z3 | 7,7´-Biphyscion | 9.30 | MS1: 565.2 [M-H]^-^ | 214, 286, 437 | CDCl_3_; δ = 12.45 (s, 2H, O*H*), 12.09 (s, 2H, O*H*), 7.67 (d, 2H, C_ar_-*H*), 7.55 (s, 2H, C_ar_-H), 7.10 (d, 2H, C_ar_-H), 3.97 (s, 6H, OCH_3_), 2.48 (s, 6H, CH_3_) |


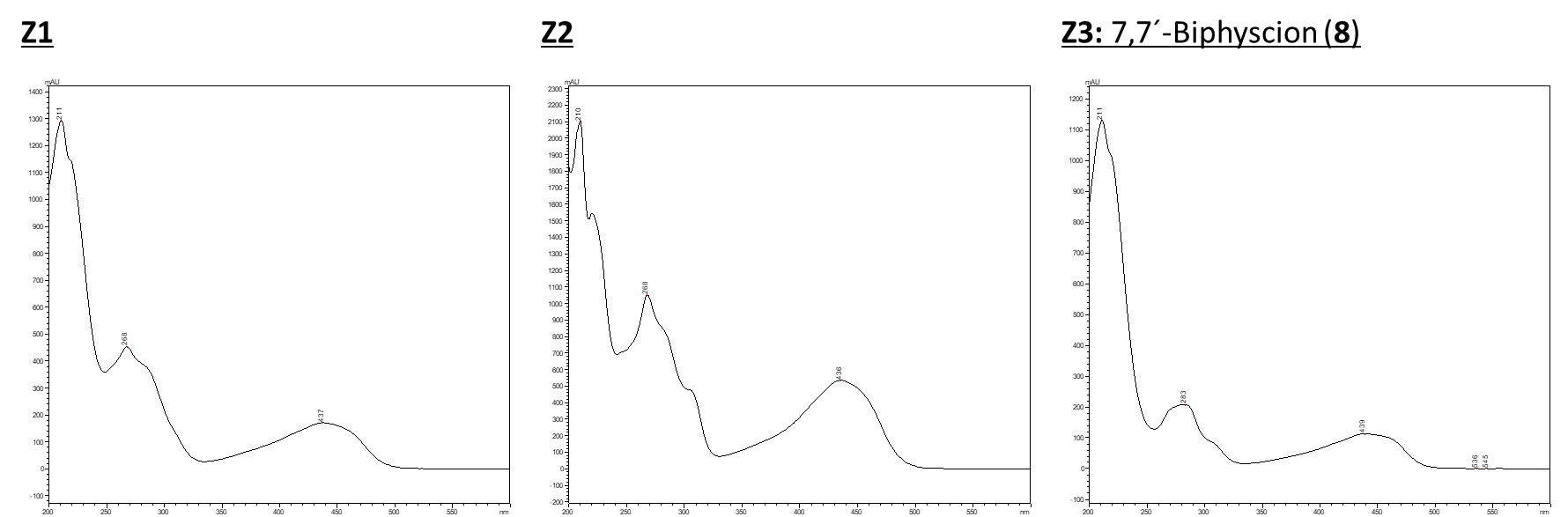


**Figure S30.** UV/Vis-spectra of Z1-3 in H_2_O/ACN + 0.1% FA (extracted from the HPLC-DAD analysis).

# References

1. Siewert, B., et al., *The Photocytotoxicity of the fungus Cortinarius rubrophyllus is concentrated in its gills.* Journal of Photochemistry and Photobiology B: Biology, 2021., submitted 09.04.2021

2. Inoue, M., et al., *Allelochemicals fromPolygonum sachalinense Fr. Schm. (Polygonaceae).* Journal of Chemical Ecology, 1992. **18**(10): p. 1833-1840.

3. Lösel, W., *UNTERSUCHUNGEN ÜBER DIE ANTHRACHINONPIGMENTE VON DERMOCYBE SANGUINEA (WULF. EX FR.) WÜNSCHE UND VERWANDTER ARTEN*, in *Fakultät für Allgemeine Wissenschaften*. 1968, Technische Hochschule München. p. 73.

4. Fabian, H., et al., *Targeted Isolation of Photoactive Pigments from Mushrooms Yielded a Highly Potent New Photosensitizer: 7,7’-Biphyscion*. 2021.

5. Steglich, W., et al., *Isolation of flavomannin-6,6′-dimethyl ether and one of its racemates from higher fungi.* Phytochemistry, 1972. **11**(11): p. 3299-3304.

6. Pachon-Pena, G., et al., *Antiproliferative effect of flavomannin-6,6'-dimethylether from Tricholoma equestre on Caco-2 cells.* Toxicology, 2009. **264**(3): p. 192-7.

7. Oertel, B., *UNTERSUCHUNGEN ZUR KONSTITUTION VON DIHYDROANTHRACENONEN UND ANGABEN ZU IHRER VERBREITUNG IN PILZEN*, in *Mathematisch-Naturwissenschaftliche Fakultät*. 1984, Friedrich-Wilhelms-Universität Bonn. p. 249.

8. Danielsen, K., D.W. Aksnes, and G.W. Francis, *NMR study of some anthraquinones from rhubarb.* Magnetic Resonance in Chemistry, 1992. **30**(4): p. 359-360.

9. Keller, G., *Pigmentationsuntersuchungen bei europäischen Arten aus der Gattung Dermocybe (FR.) WÜNSCHE.* 1982: p. 110-126.
